# Protein homeostasis maintained by HOOK1 levels promotes the tumorigenic and stemness properties of ovarian cancer cells through reticulum stress and autophagy

**DOI:** 10.1186/s13046-024-03071-2

**Published:** 2024-05-29

**Authors:** Elisa Suárez-Martínez, Sander R. Piersma, Thang V. Pham, Irene V. Bijnsdorp, Connie R. Jimenez, Amancio Carnero

**Affiliations:** 1 Instituto de Biomedicina de Sevilla (IBIS), HUVR/CSIC/Universidad de Sevilla, Avda. Manuel Siurot S/N; Campus HUVR, Ed. IBIS,, Seville, 41013 Spain; 2 CIBER de Cancer (CIBERONC), Instituto de Salud Carlos III, Madrid, Spain; 3 OncoProteomics Laboratory, VUmc-Cancer Center Amsterdam, VU University Medical Center, CCA 1-60, De Boelelaan 1117, 1081HV, Amsterdam, The Netherlands

**Keywords:** Ovarian cancer, HOOK1, Stemness, ER stress

## Abstract

**Background:**

Ovarian cancer has a high mortality rate mainly due to its resistance to currently used therapies. This resistance has been associated with the presence of cancer stem cells (CSCs), interactions with the microenvironment, and intratumoral heterogeneity. Therefore, the search for new therapeutic targets, particularly those targeting CSCs, is important for improving patient prognosis. HOOK1 has been found to be transcriptionally altered in a substantial percentage of ovarian tumors, but its role in tumor initiation and development is still not fully understood.

**Methods:**

The downregulation of HOOK1 was performed in ovarian cancer cell lines using CRISPR/Cas9 technology, followed by growth in vitro and in vivo assays. Subsequently, migration (Boyden chamber), cell death (Western-Blot and flow cytometry) and stemness properties (clonal heterogeneity analysis, tumorspheres assay and flow cytometry) of the downregulated cell lines were analysed. To gain insights into the specific mechanisms of action of HOOK1 in ovarian cancer, a proteomic analysis was performed, followed by Western-blot and cytotoxicity assays to confirm the results found within the mass spectrometry. Immunofluorescence staining, Western-blotting and flow cytometry were also employed to finish uncovering the role of HOOK1 in ovarian cancer.

**Results:**

In this study, we observed that reducing the levels of HOOK1 in ovarian cancer cells reduced in vitro growth and migration and prevented tumor formation in vivo. Furthermore, HOOK1 reduction led to a decrease in stem-like capabilities in these cells, which, however, did not seem related to the expression of genes traditionally associated with this phenotype. A proteome study, along with other analysis, showed that the downregulation of HOOK1 also induced an increase in endoplasmic reticulum stress levels in these cells. Finally, the decrease in stem-like properties observed in cells with downregulated HOOK1 could be explained by an increase in cell death in the CSC population within the culture due to endoplasmic reticulum stress by the unfolded protein response.

**Conclusion:**

HOOK1 contributes to maintaining the tumorigenic and stemness properties of ovarian cancer cells by preserving protein homeostasis and could be considered an alternative therapeutic target, especially in combination with inducers of endoplasmic reticulum or proteotoxic stress such as proteasome inhibitors.

**Supplementary Information:**

The online version contains supplementary material available at 10.1186/s13046-024-03071-2.

## Introduction

Ovarian cancer is the most lethal of all gynecological tumors, ranking as the seventh most common cancer and the fifth leading cause of cancer-related death in women worldwide [1]. Its high mortality rate is largely due to late detection and resistance to therapies [2]. The standard treatment involves total tumor resection combined with chemotherapy and, in recent years, targeted therapies have emerged, showing benefits in ovarian cancer treatment [3, 4]. However, despite these treatments, the majority of patients experience recurrence, develop chemoresistance, and respond only moderately to second-line therapy [5].Numerous studies support the existence of cancer stem cells (CSCs) in ovarian tumors [6–9], proposing that this CSCs have acquired the capacity of indefinite self-renewal and can give rise to intermediate progenitors and differentiated cells, contributing to tumor heterogeneity. CSCs also have a greater ability to resist intrinsic and extrinsic damage and exhibit reduced sensitivity to radiotherapy, cytotoxic agents, and targeted therapy [10]. Therefore, it is crucial to discover new therapeutic approaches that specifically target CSCs within a tumor.

By studying ovarian cancer databases, we found that various proteins from the HOOK family were overexpressed and amplified in ovarian tumors, prompting exploration of their potential association with resistance to conventional therapies. The evolutionarily conserved HOOK protein family in humans comprises three proteins (HOOK1, HOOK2, and HOOK3), each characterized by three regions. Their N-terminal domain is capable of indirectly binding to the cell’s microtubules through dynein-dynactin and kinesin-3 motors, which are involved in cellular transport [11]. Their central supercoiled helix domain allows them to form homodimers and heterodimers [12]. Lastly, their C-terminal domain is involved in the binding of each HOOK protein to different types of organelles due to its greater evolutionary divergence. While different HOOK paralogs exhibit distinct subcellular localizations and functions, they can also form a complex with other proteins, known as the FHF complex [13, 14]. HOOK1 is primarily associated with the regulation of clathrin-independent endocytosis (CIE) [15–17], a crucial process for immune surveillance, cell migration, metastasis, and cell signaling [17, 18]. HOOK1 can bind to some proteins internalized by CIE through its C-terminal domain and facilitate their recycling back to the plasma membrane, thereby increasing their lifespan [15, 19].

The role of HOOK1 in cancer has not been explored in depth. However, a few studies have linked this protein to different types of tumors. For instance, a decrease in HOOK1 has been observed in hepatocellular carcinoma, where its downregulation has been associated with increased malignancy [20]. Furthermore, HOOK1 has been proposed as a biomarker in papillary mucinous neoplasm following a proteomic screening [21]. Nonetheless, the mechanism by which HOOK1 may be involved in cancer remains unclear. The most studied hypothesis in this regard is the association between HOOK1 and the phosphatase SHP2, implicated in tumorigenesis and metastasis in different types of tumors [22–24]. However, the function of SHP2 as an oncogene or tumor suppressor gene is controversial and apparently depends on the tumor type. It has been described that HOOK1 is capable of binding to SHP2, and it has been suggested that it acts as an inhibitor of its phosphatase activity. In lung cancer cells, it was observed that while SHP2 positively regulates TEM, HOOK1 has the opposite effect [24]. Nonetheless, other studies have linked HOOK1 to the progression of different types of tumors independently to its proposed interaction with SHP2 [20, 25, 26]. Hence, the precise role of HOOK1 in cancer and its underlying mechanism remain unclear.

In this study, we characterized the importance of HOOK1 for the aggressive phenotype of ovarian cancer. For this purpose, we decreased HOOK1 levels in ovarian cancer cell lines and observed that this reduction affects their proliferative and survival capabilities. Additionally, this effect appears to decrease the stem cell-like properties of the cells. We also found that HOOK1 downregulation causes an increase in endoplasmic reticulum (ER) stress and changes in autophagic flux, which could explain the previously described phenotype. Therefore, HOOK1 seems to be important for the maintenance of ovarian tumors, and its inhibition could be a promising therapeutic strategy.

## Results

### HOOK1 affects the tumorigenic properties of ovarian cancer cells in vitro and impairs their ability to form tumors in vivo

**Fig. 1:**
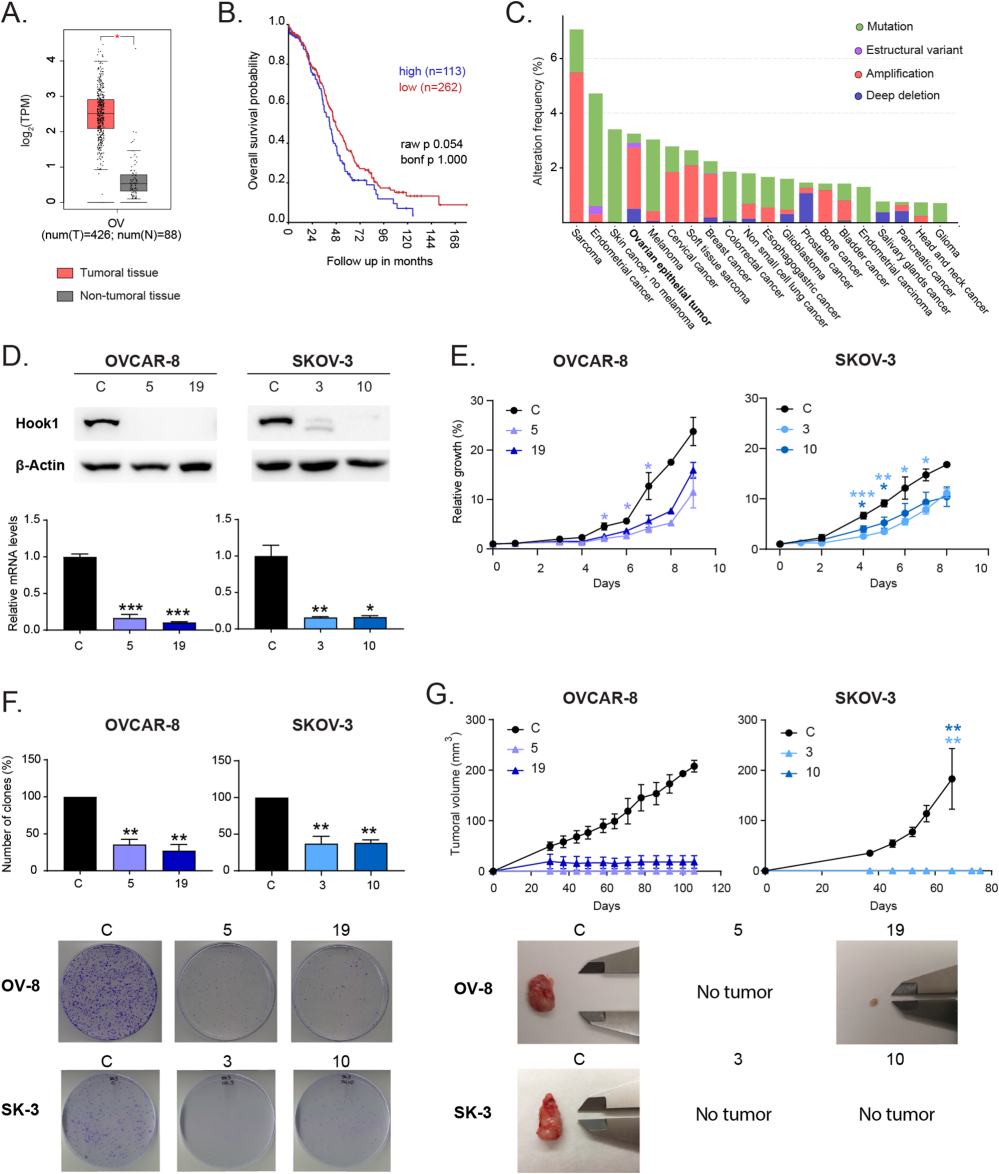
Hook1 is overexpressed in ovarian cancer patients, and its downregulation impairs the growth of ovarian cancer cells and abolishes the generation of tumors in vivo. (**A**) Comparison of the expression of Hook1 in ovarian tumors vs. normal tissue. (**B**) Overall survival of patients with high vs. low expression of HOOK1. (**C**) Analysis of Hook1 alterations in various human tumors. (**D**) Validation of Hook1 downregulation in ovarian cancer cells by WB and RT‒qPCR analyses. (**E**) Colony formation ability and (**F**) growth curve analysis of ovarian cancer cells with Hook1 downregulation. The mean of 3 independent experiments ± SEM is presented. (**G**) Evaluation of tumor growth of xenografts generated from Hook1-downregulated cells in comparison to parental cell lines. Graphs represent the tumor size (mean ± SEM). Representative images of the tumors are shown. Statistical analysis was performed with Student’s t test (*p < 0.05; **p < 0.01; ***p < 0.001). The absence of an asterisk means that the data are not statistically significant

In previous studies, HOOK1 has been suggested to be linked to platinum resistance in ovarian tumors [27]. To undercover its significance in this type of cancer, firstly, we studied it in multiple databases. We observed that HOOK1 expression was significantly higher in ovarian tumors than in normal tissue (Fig. 1A) and that the overall survival tends to be worse in tumors where this gene is highly expressed (Fig. 1B). In addition, we found that there was a high percentage of ovarian tumors with amplifications in this gene (Fig. 1C). Altogether, these data show that there is a high percentage of ovarian tumors with HOOK1 alterations and expression changes and that these modifications may be associated with a worse prognosis in patients.

Consequently, we decided to analyze the impact of decreasing the levels of this gene on the tumorigenic properties of the ovarian cancer cell lines OVCAR-8 and SKOV-3. To generate cells with reduced levels of HOOK1, we used the CRISPR‒Cas9 technique. The clones used for this study were selected among many by Western Blot (WB) analysis and sequenced to confirm the mutation of the gene (Supp. Figure 1). In the OVCAR-8 cell line, a complete elimination of the protein was achieved, while in the SKOV-3 cell line, a very significant decrease in protein levels was observed. Additionally, in both cell lines, we observed very low residual mRNA levels compared to those of the parental line (Fig. 1D). We observed that the cells with downregulated HOOK1 had a significant reduction in growth (Fig. 1E) and the ability to form colonies (Fig. 1F) in the two studied cell lines. Furthermore, we investigated whether these alterations were reproducible in vivo by generating xenotransplants in immunodeficient mice. We observed that, in all cases, cells with reduced levels of HOOK1 were unable to form tumors, while parental cells formed tumors normally (Fig. 1G). These data suggest that HOOK1 is necessary for the growth of ovarian cancer cells both in vitro and in vivo.

**Fig. 2:**
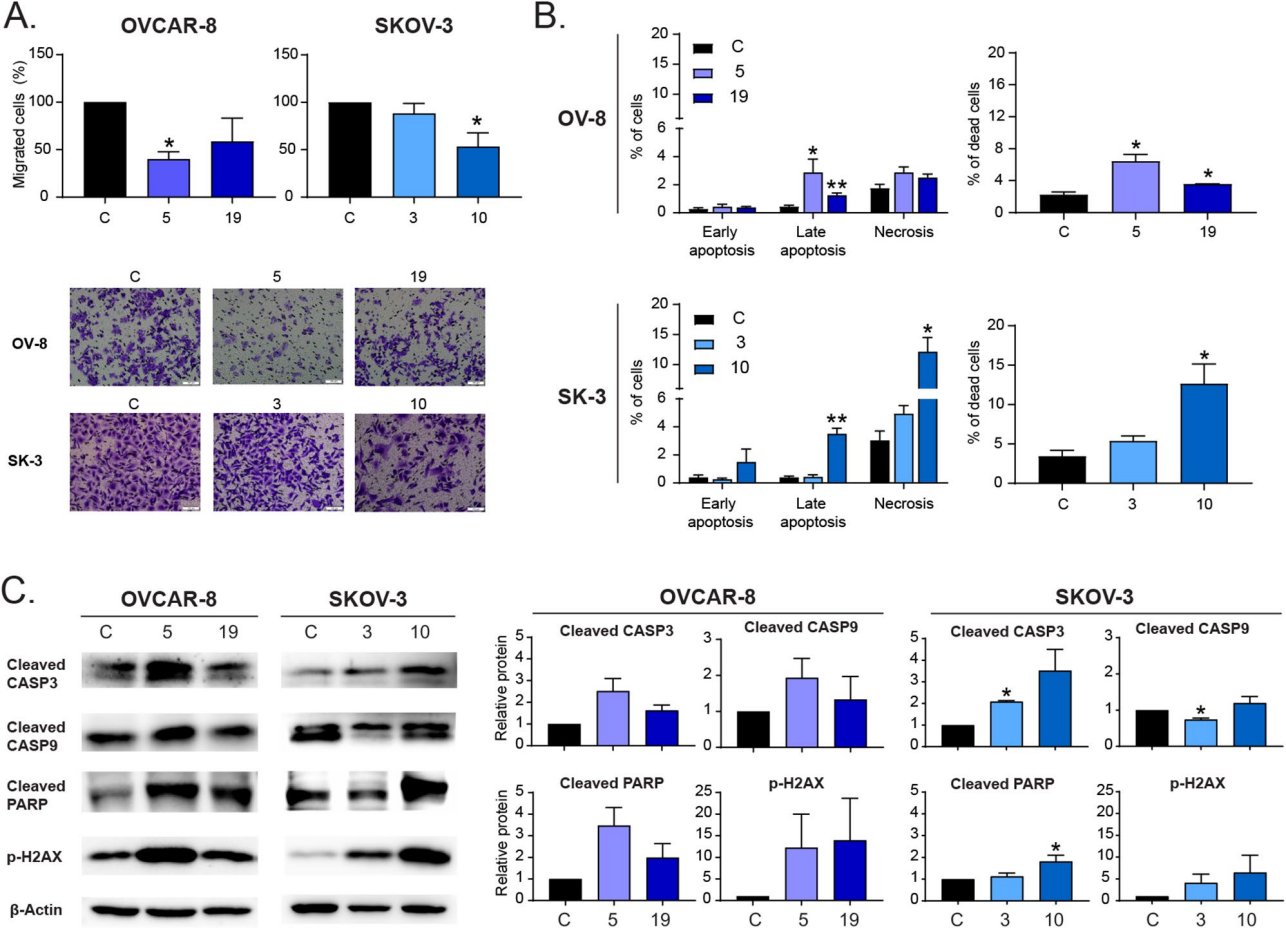
Hook1 downregulation impairs the migration of ovarian cancer cells and increases cell death. (**A**) Boyden assay of ovarian cancer cells with Hook1 downregulation. Representative microscopic images are shown (20x). (**B**) Quantification by flow cytometry of the percentage of apoptotic and necrotic cells, as well as total cell death, when Hook1 is downregulated. (**C**) Protein levels of markers associated with apoptotic cell death upon Hook1 downregulation. The mean of 3 independent experiments ± SEM is represented. Statistical analysis was performed with Student’s t test (*p < 0.05; **p < 0.01; ***p < 0.001). The absence of an asterisk means that the data are not statistically significant

Other crucial hallmarks of tumoral cells are their capability to migrate and develop metastases and their ability to escape programmed cell death. Using a Boyden assay, we observed a decrease in the migratory capacity of all the cells with reduced levels of HOOK1 (Fig. 2A). Additionally, we studied whether HOOK1 could affect the survival of ovarian cancer cells. For this purpose, we performed a flow cytometry assay using Annexin-V and a DNA intercalating agent. We observed that cells with reduced levels of HOOK1 showed an increase in the percentage of dead cells, both through apoptotic and nonapoptotic cell death (Fig. 2B, Supp. Figure 2A, Supp. Figure 11). Then, we investigated the alteration of proteins related to apoptotic pathways, such as cleaved CASP3, CASP9, PARP, and p-H2AX. Through WB analysis, we found an increase in the quantity of all the studied markers (Fig. 2C). Thus, it appears that the reduction in HOOK1 increases the activation of the apoptotic pathway in ovarian cancer cells and leads to an increase in cell death, both through apoptosis-dependent and apoptosis-independent mechanisms.

In summary, HOOK1 is involved in the maintenance of ovarian cancer cell growth and migration, and its downregulation negatively impacts these cells, causing a significant increase in cell death and preventing the formation of tumors in vivo.

### Hook1 downregulation reduces CSC-associated properties in ovarian cancer cells

**Fig. 3:**
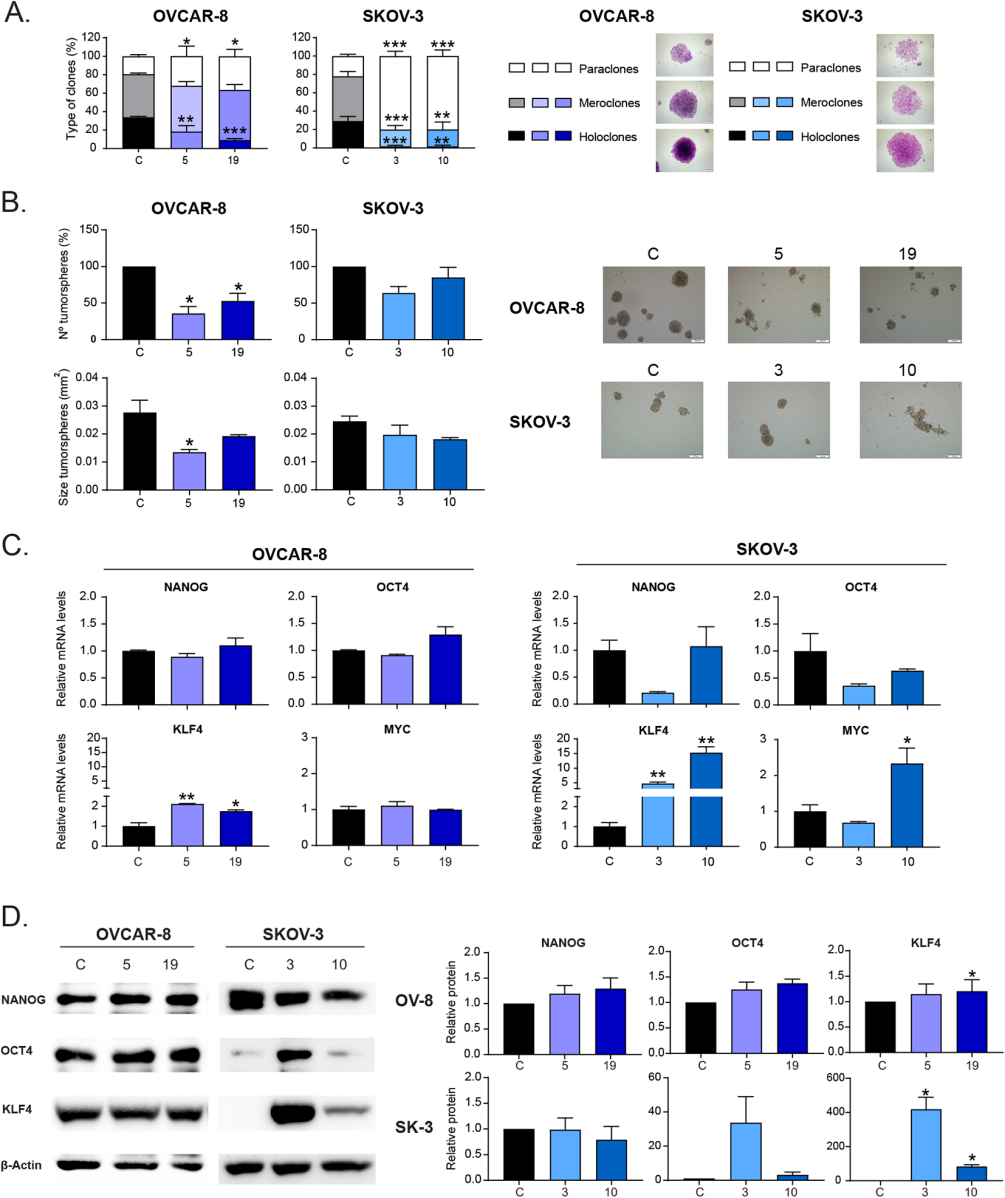
Hook1 downregulation causes a decrease in CSC-associated properties independent of the expression of genes traditionally linked to the CSC phenotype. (**A**) Analysis of the type of clones originated from a clonability assay of ovarian cancer cell lines with downregulated Hook1. The percentage of holoclones, meroclones and paraclones was assessed. Representative images of each type of clone are shown. (**B**) Analysis of the number and size of tumorspheres of ovarian cancer cells with downregulated Hook1. (**C**) Relative mRNA levels and (**D**) relative protein levels of factors traditionally associated with stemness in ovarian cancer cell lines with downregulated Hook1. The mean of 3 independent experiments ± SEM is represented. Statistical analysis was performed with Student’s t test (*p < 0.05; **p < 0.01; ***p < 0.001). The absence of an asterisk means that the data are not statistically significant

Due to the previously found physiological alterations, we wondered whether HOOK1 might also play a role in the generation or maintenance of CSCs in ovarian tumors. For that purpose, we analyzed the proportion of different types of clones formed after seeding the cells at low density [28–30] and performed a tumorsphere assay. In the clonability assay, we observed a significant decrease in the number of holoclones (clones enriched in CSCs) and an increase in the number of paraclones (clones enriched in differentiated non-stem cells) under all conditions with reduced levels of HOOK1 (Fig. 3A). Additionally, in the tumorsphere assay, we observed that cells with reduced levels of HOOK1 had a diminished capacity to form these structures and that there was a reduction in the size of tumorspheres in the OVCAR-8 cell line (Fig. 3B). These results suggest that downregulation of HOOK1 specifically affects CSCs and causes a decrease in the stem-associated phenotype.

Next, we analyzed the mRNA levels of genes commonly involved in the generation of CSCs due to their involvement in the dedifferentiation process, such as the Yamanaka factors (*OCT4*, *KLF4*, and *c-MYC*) and the transcription factor *NANOG*. Surprisingly, we did not find a significant decrease in these genes when HOOK1 levels were reduced. In fact, in some cases, there was an increase, such as in *KLF4*, which significantly increased in all clones with downregulated HOOK1 in both cell lines (Fig. 3C). To confirm the results obtained by q-PCR, we performed WB analysis for some of the studied genes. We observed that there was an increase in the amount of OCT4 and KLF4 proteins in the SKOV-3 cell line. However, we did not observe significant changes in any of these proteins in the OVCAR-8 line (Fig. 3D). Therefore, the involvement of HOOK1 in diminishing stem properties in ovarian cancer cells seems to be independent of the expression of these genes conventionally linked to stemness, suggesting the presence of an alternative mechanism to account for such alterations.

### Hook1 downregulation induces UPR activation and sensitizes cells to drugs that increase proteotoxic stress

**Fig. 4:**
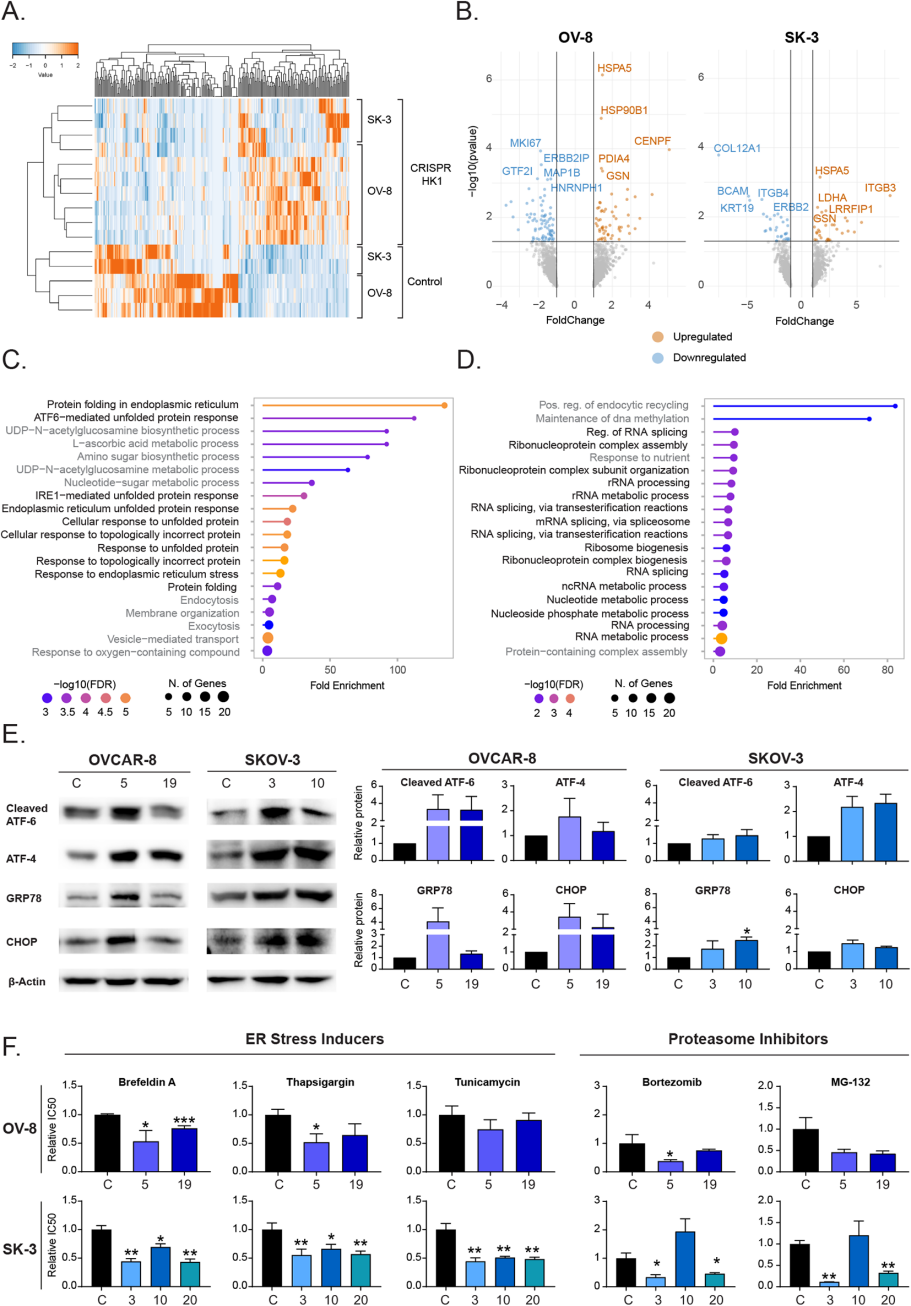
Increased ER stress and sensitivity to proteotoxic stress-inducing drugs upon Hook1 downregulation. (**A**) Supervised clustering of proteins upregulated and downregulated when Hook1 levels are reduced. (**B**) Volcano plot displaying upregulated (FC > 1) and downregulated (FC < -1) proteins resulting from Hook1 downregulation (p value < 0.05). The names of the top 10 most significantly upregulated or downregulated proteins are showed. (**C**) Gene Ontology analysis of proteins upregulated and (**D**) downregulated upon Hook1 downregulation in OVCAR-8 cell line. (**E**) Protein levels of UPR-associated proteins in Hook1-downregulated cells. (**F**) IC50 values of drugs that induce ER stress or inhibit proteasome function in Hook1-downregulated cells. The mean of 3 independent experiments ± SEM is represented. Statistical analysis was performed with Student’s t test (*p < 0.05; **p < 0.01; ***p < 0.001). The absence of an asterisk means that the data are not statistically significant

To investigate the mechanism of action of HOOK1 in cancer, we conducted a study of the proteome of cells with downregulated HOOK1. This analysis identified 6352 proteins, of which approximately 100 proteins were significantly altered in the comparison of parental and CRISPR down-modulated cells in each cell line. In the supervised clustering of the altered proteins, we observed a substantial number of proteins whose levels changed according to the levels of HOOK1 (Fig. 4A). To associate these to biological processes and functions, we selected significant proteins with increased or decreased expression (Fig. 4B), as input for Gene Ontology analysis. Among the upregulated proteins in the OVCAR-8 cell line, we found an increase in numerous terms related to misfolded proteins and protein folding in the endoplasmic reticulum (ER). We also found several terms associated with metabolic processes, vesicle transport and cytoskeleton organization (Fig. 4C, Supp. Figure 3A, Supp. Figure 4A). For downregulated proteins, we found terms mainly related to RNA processing or translational regulation (Fig. 4D, Supp. Figure 3B, Supp. Figure 5A). In the case of the SKOV-3 cell line, we also observed an increase in proteins related to ER protein folding, along with terms associated with cytoskeleton organization and regulation of cell death. We also found terms related to vesicle transport and mesenchymal migration (Supp. Figure 3A, Supp. Figure 4B, Supp. Figure 6A). A substantial number of downregulated proteins were also associated with RNA processing and regulation of translation, as in the OVCAR-8 cell line (Supp. Figure 3B, Supp. Figure 5B, Supp. Figure 6B).

**Fig. 5:**
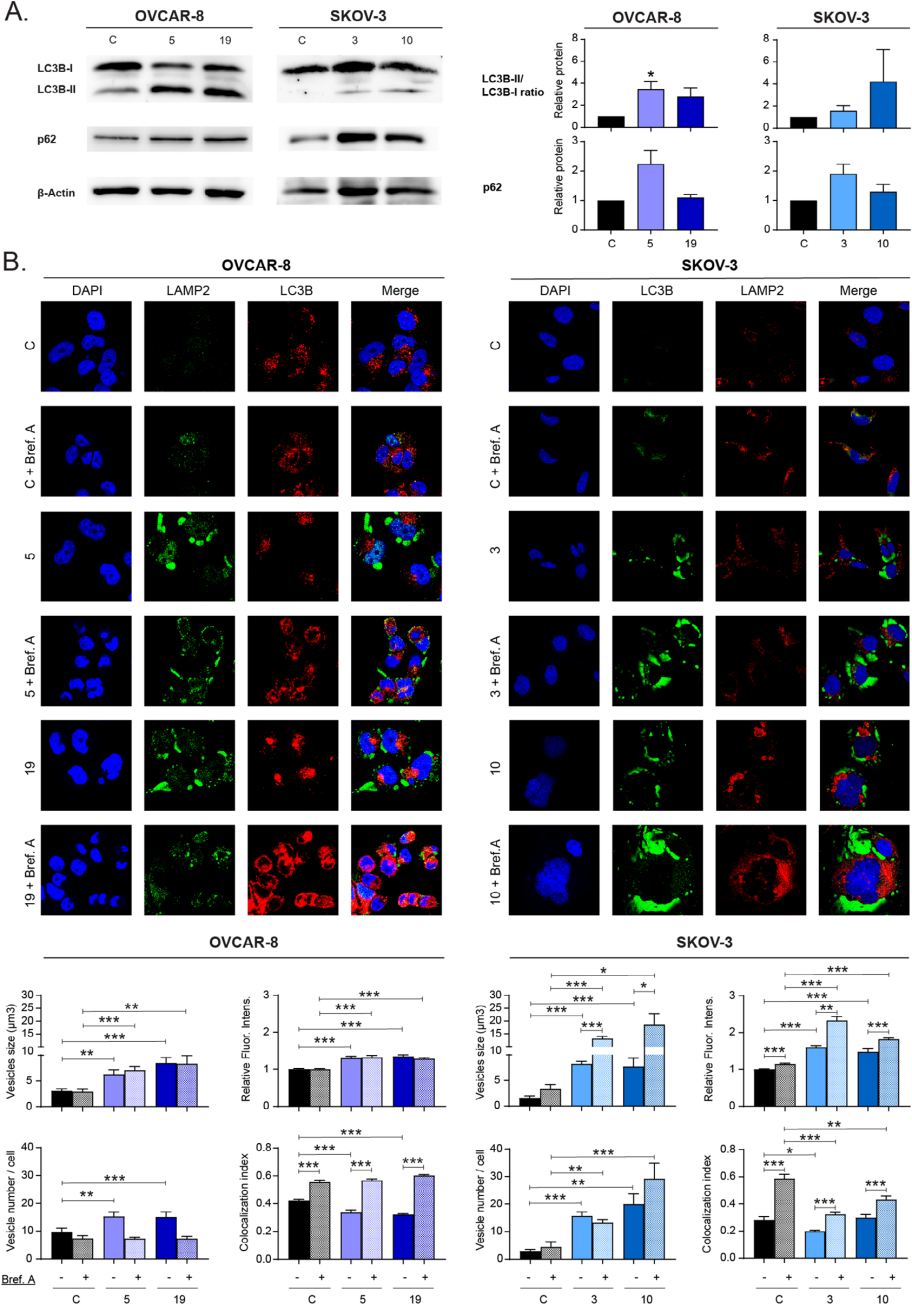
Context-dependent changes in autophagic flux upon Hook1 downregulation. (**A**) Protein levels of autophagy-associated proteins in Hook1-downregulated cells. (**B**) Immunofluorescence staining of LC3B (green) and LAMP2 (red) proteins in Hook1-downregulated cells treated with brefeldin A. DAPI (blue) was used as a nuclear stain, and the merge of the 3 markers is shown. The autophagosome size, number and relative fluorescence intensity, as well as the colocalization index of autophagosomes-lysosomes, were measured using ImageJ software. At least 100 cells of each condition were analyzed, and the statistical analysis was performed using Student’s t test (*p < 0.05; **p < 0.01; ***p < 0.001)

**Fig. 6:**
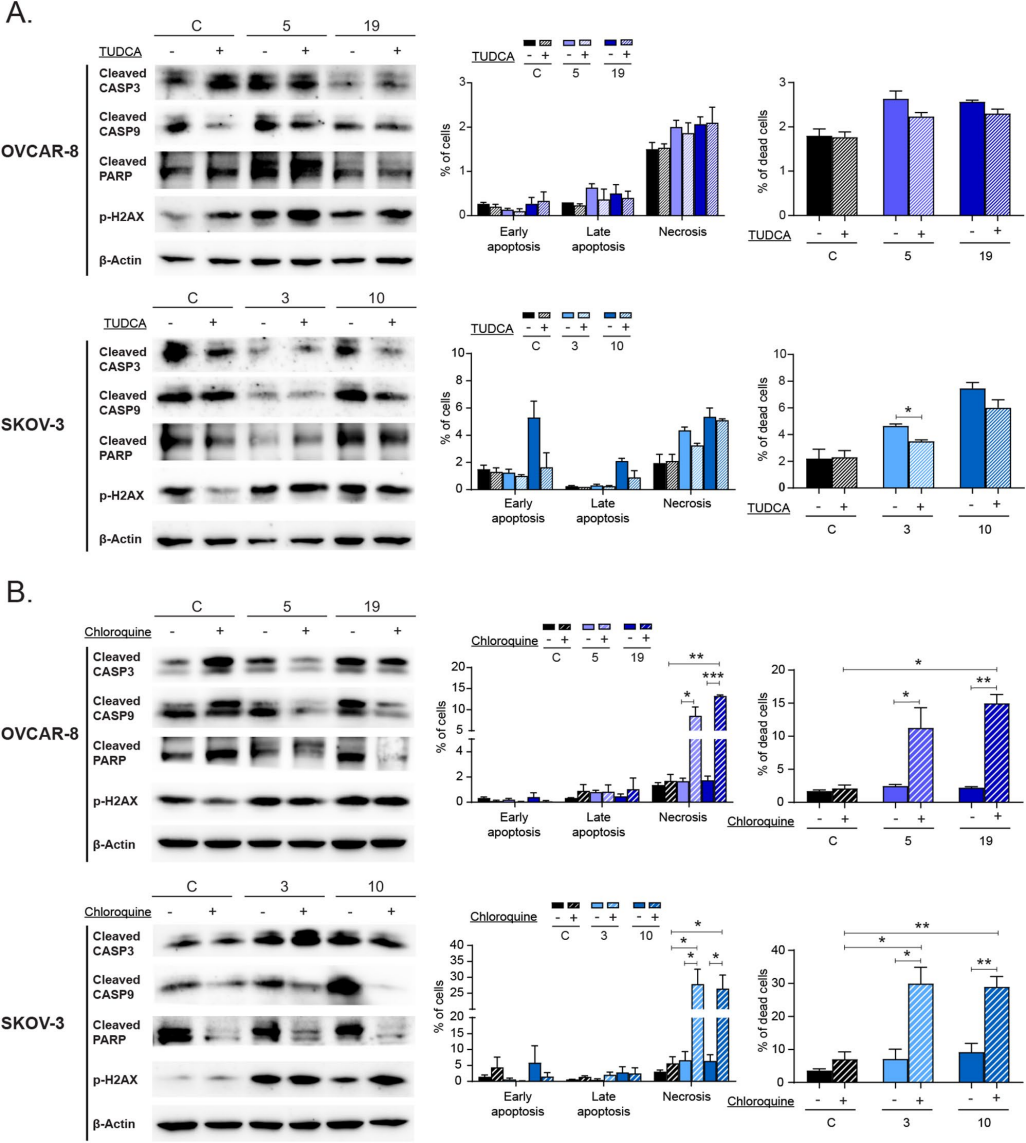
Cell death induced by Hook1 downregulation is dependent on the ER stress response and autophagic flux. (**A**) Analysis of cell death in Hook1-downregulated cells with relieved ER stress. Protein levels of apoptotic markers in Hook1-downregulated cells treated with TUDCA (left). Quantification by flow cytometry of the percentage of apoptotic and necrotic cells, as well as total cell death, in Hook1-downregulated cells treated with TUDCA (right). (**B**) Analysis of cell death in Hook1-downregulated cells with blocked autophagic flux. Protein levels of apoptotic markers in Hook1-downregulated cells treated with chloroquine (left). Quantification by flow cytometry of the percentage of apoptotic and necrotic cells, as well as total cell death, in Hook1-downregulated cells treated with chloroquine (right). The mean of 3 independent experiments ± SEM is represented. Statistical analysis was performed with Student’s t test (*p < 0.05; **p < 0.01; ***p < 0.001). The absence of an asterisk means that the data are not statistically significant

Therefore, in both cell lines, we observed an increase in the response to misfolded proteins and a decrease in proteins related to RNA regulation and translation. Moreover, when we analyzed the common proteins that were upregulated in both cell lines when HOOK1 levels are reduced, we found that they were predominantly chaperons involved in protein folding and, also, some proteins related to the cytoskeleton organization (Supp. Figure 6C). These data suggest that the reduction in HOOK1 levels may lead to an accumulation of misfolded proteins that could activate the unfolded protein response (UPR) in the ER. To further investigate this possibility, we analyzed some proteins involved in the UPR signaling pathways by WB analysis. We found that in the SKOV-3 cell line, the four proteins analyzed increased in both CRISPR clones. In the OVCAR-8 cell line, we also observed an increase in ATF-4, ATF-6 and CHOP levels in many of the clones analyzed (Fig. 4E, Supp. Figure 2B). These results reinforce the hypothesis that the reduction in HOOK1 levels triggers ER stress.

Consequently, we investigated whether the decrease in HOOK1 could sensitize ovarian cancer cells to ER stress-inducing agents. For this purpose, we calculated the IC50 (half-maximal inhibitory concentration) of three commonly employed drugs to study this mechanism: brefeldin A, thapsigargin, and tunicamycin. We observed that cells with reduced levels of HOOK1 were more sensitive to treatment with ER stress-inducing agents in all cases and with all of the treatments (Fig. 4F, Supp. Figure 7). Altogether, the activation of UPR signaling observed through both WB analysis and proteomics, along with the heightened sensitivity to these drugs, provides solid confirmation of the elevated ER stress levels in ovarian cancer cell lines when HOOK1 levels are reduced.

**Fig. 7:**
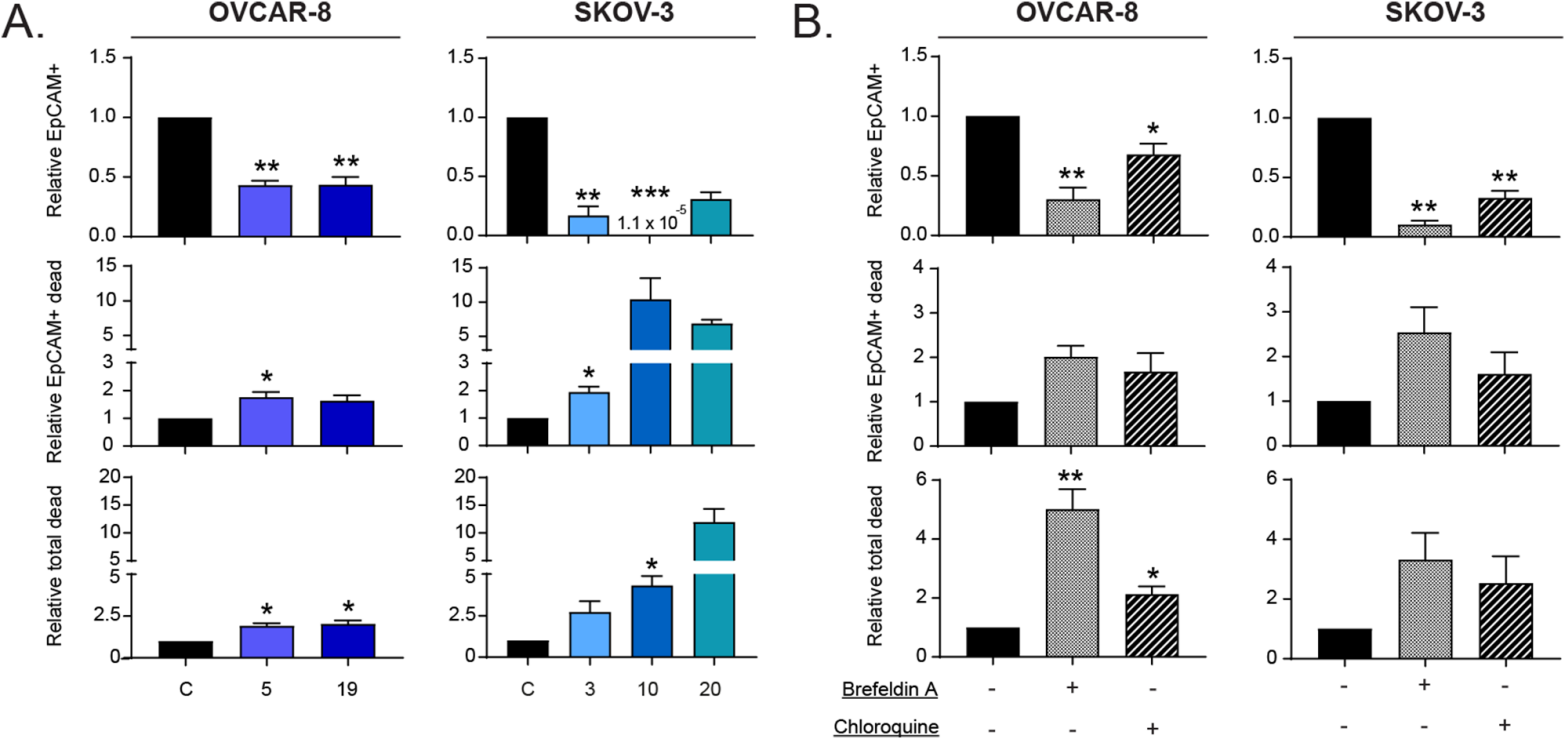
Cell death induced by Hook1 downregulation may selectively impact CSCs. (**A**) Quantification by flow cytometry of the relative number of cells expressing the CSC marker EpCAM, EpCAM + dead cells, and total dead cells in Hook1-downregulated cells. (**B**) Quantification by flow cytometry of the relative number of parental cells expressing the CSC marker EpCAM, EpCAM + dead cells, and total dead cells after the treatment with an ER stress inducer (Brefeldin A) or an autophagic flux blocker (chloroquine). The mean of 3 independent experiments ± SEM is represented. Statistical analysis was performed with Student’s t test (*p < 0.05; **p < 0.01; ***p < 0.001). The absence of an asterisk means that the data are not statistically significant

Furthermore, we analyzed the sensitivity of the studied cells to proteasome inhibitors because inhibiting proteasome activity should lead to an increased accumulation of misfolded proteins and, consequently, ER stress. We observed that cells with downregulated HOOK1 were more sensitive to bortezomib and MG-132. Only clone 10 of SKOV-3 appeared to be equally resistant to both compounds as the control cells. Hence, we investigated another HOOK1 CRISPR clone in this line, and we confirmed that it exhibited an increased sensitivity to the three ER stress inducers employed and to bortezomib and MG-132 (Fig. 4F, Supp. Figure 7). Therefore, overall, the reduction in HOOK1 levels seems to sensitize ovarian cancer cells to both ER stress-inducing drugs and proteasome inhibitors.

### Hook1 downregulation causes changes in autophagic flux

When cells undergo ER stress, a series of mechanisms are activated to restore cellular homeostasis. For example, autophagy can be activated to reduce ER swelling and promote cell survival [31]. Therefore, we investigated whether cells with downregulated HOOK1 presented changes in autophagic flux. First, we measured the LC3B-II/LC3B-I ratio and P62 levels using WB analysis. When we decreased HOOK1 expression, we observed an increase in the LC3-II/LC3-I ratio in both cell lines (Fig. 5A). This finding may indicate an elevation of autophagic flux, which is consistent with UPR-mediated autophagic activation. However, we also observed higher levels of the protein P62, especially in the SKOV-3 cell line (Fig. 5A), which could indicate that there is some blockage in autophagic flux.

To determine which of these two scenarios occurs in our model, we added brefeldin A to the cells to further increase ER stress and stimulate autophagy (Supp. Figure 8) and analyzed autophagosome-lysosome fusion by immunofluorescence. We observed that cells with reduced HOOK1 levels, even without receiving any treatment, presented a higher number of autophagosomes than normal cells, and these vesicles were significantly larger than those observed in the parental cells. Furthermore, in the SKOV-3 cell line treated with the drug, the number, size, and fluorescence intensity of these vesicles increased even further, surpassing the levels found in the control cells. However, we found that autophagosome-lysosome colocalization was reduced in cells with downregulated HOOK1, especially in cell line SKOV-3, where the addition of Brefeldin A was not able to replicate the colocalization levels observed in the control cells (Fig. 5B, Supp. Figure 2C). This finding suggests that there is some blockade in the autophagic flux when HOOK1 is downregulated. This blockade would prevent the fusion of autophagosomes with lysosomes, leading to an accumulation of both structures.

**Fig. 8:**
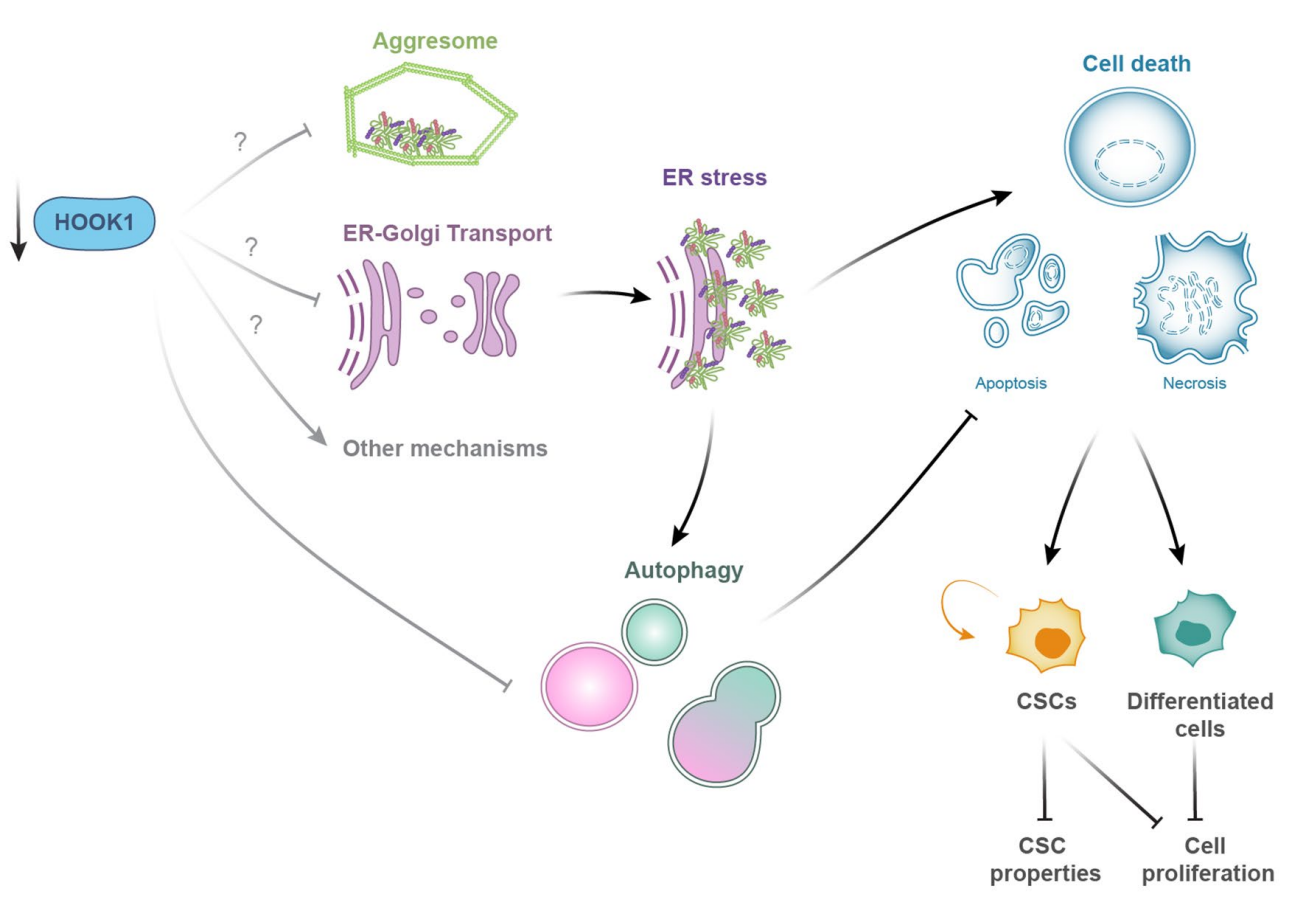
Interaction of HOOK1 with ER stress, autophagy, and cell death. Decreased HOOK1 levels lead to the activation of ER stress, which activates signaling pathways that result in increased cell death and autophagy. Autophagy acts as a protective mechanism for the cell, reducing protein load and thus contributing to cell survival. However, the decrease in HOOK1 may also interfere with autophagic flux, causing a partial blockage of it and, therefore, reducing the effectiveness of this mechanism in preventing cell death. The increased cell death affects both differentiated cells and CSCs, causing a reduction of the cell proliferation and CSC properties of ovarian cancer cells

### Cell death induced by Hook1 downregulation is dependent on the ER stress response and autophagic flux

As previously demonstrated, reducing HOOK1 levels activates apoptotic signaling pathways, leading to increased cell death (Fig. 4B and C). To investigate whether the activation of the UPR is responsible of this death, we used tauroursodeoxycholic acid (TUDCA) to reduce ER stress levels. Although TUDCA effectively reduced ER stress in cells with downregulated HOOK1 (Supp. Figure 9), its impact on cell death *shows some clonal variability*. In OVCAR-8 cells, apoptotic markers showed no significant changes, and TUDCA seemed to reduce cell death in clone 5 but had unclear effects in clone 19. In SKOV-3 cells, TUDCA reduced cleaved CASP3 and CASP9 and flow cytometry revealed reduced cell death in both clones, with clone 10 showing primarily a decrease in early-stage apoptosis and clone 3 displaying reduced necrosis (Fig. 6A). Altogether, these findings suggest that inhibiting ER stress attenuates cell death in cells with reduced HOOK1 levels, albeit not completely rescuing the phenotype.

In contrast, activation of autophagic flux has been reported to serve as a survival mechanism in cells with high ER stress. However, we found an impeded autophagic flux when we decreased HOOK1 levels (Fig. 5B, Supp. Figure 2C). Therefore, in order to determine whether autophagy contributes protectively or inductively to cell death, we used chloroquine as an inhibitor of autophagic flux [32]. We first confirmed its effectiveness by WB and immunofluorescence analyses (Supp. Figure 10). Subsequently, we observed that when we added chloroquine to cells with reduced HOOK1 levels, some apoptotic markers, such as CASP9 and PARP, were reduced in both cell lines (Fig. 6B). However, by flow cytometry, we observed an increase in the percentage of dead cells after the treatment, which was significantly more pronounced in the cells with reduced HOOK1 levels. This increase was not due to the activation of apoptosis; instead, we found a very significant increase in the number of necrotic cells (Fig. 6B). Therefore, these data suggest that cells with reduced HOOK1 levels are more sensitive to the blockage of autophagic flux, which likely acts as a protective mechanism to restore cellular proteostasis. This result, coupled with the increase of vesicle colocalization in CRIPSR clones following the addition of brefeldin A (Fig. 5B, Supp. Figure 2C), indicates that autophagic flux is not completely blocked in cells with reduced levels of HOOK1, retaining some degree of functionality.

### CSCs might be particularly affected by the cell death induced by Hook1 downregulation

Our data provide evidence that reducing HOOK1 leads to a decrease in cancer stem-like properties in ovarian cancer cells. However, this phenomenon was not dependent on genes traditionally associated with dedifferentiation. Therefore, we studied the link between the reduction in stem-like capabilities and the occurrence of ER stress-associated cell death upon HOOK1 downregulation. For that purpose, we analyzed the percentage of dead cells positive for EpCAM, a CSC marker. In both ovarian cancer cell lines, we observed that downregulating HOOK1 caused a decrease in the percentage of EpCAM + cells compared to that of parental cells. The analysis of cell death specifically in EpCAM + cells revealed an approximately 2-fold increase in cell death in most HOOK1 CRISPRs in comparison to parental cells. Strikingly, clone 10 of SKOV-3 cell line showed a 10-fold increase in cell death compared to that of the parental line. This result correlates with the very reduced percentage of EpCAM + cells that we found in said clone (Fig. 7A). These data support the hypothesis that the reduction in HOOK1 leads to a preferential decrease in CSCs in ovarian cancer due to an increase in their cell death, probably due to unsustainable ER stress.

As we observed changes in ER stress and the state of autophagic flux in our model, we wondered whether these changes were the cause of the increase in cell death in CSCs. Therefore, we measured the levels of cell death in EpCAM + cells treated with brefeldin A and chloroquine to increase ER stress or block autophagic flux, respectively. We observed a decrease in the percentage of EpCAM-positive cells with both treatments. Moreover, the number of dead cells marked with EpCAM increased, especially with brefeldin A (Fig. 7B). Thus, we concluded that increasing ER stress and partially blocking autophagic flux could also increase cell death in cells with stem-like properties. Altogether, it appears that the reduction in stem-associated properties that occurs when HOOK1 levels are decreased might be due to an increase in CSC death and that this death could be caused by an elevation in ER stress or a partial blockage of autophagic flux (see Fig. 8).

## Discussion

The downregulation of HOOK1 in ovarian cancer cell lines has resulted in a decrease in their tumorigenic and stemness properties while significantly increasing cell death. However, we have not found alterations in the expression of genes traditionally related to the stem cell phenotype that could explain this phenomenon. In some cases, we even found an increase of the expression of those genes, as in the case of KLF4. This could be explained by the increased ER stress observed when HOOK1 levels decrease. A recent study linked increased ER stress to the induction of KLF4 expression in melanoma cells, as an adaptive mechanism inhibiting UPR-induced apoptosis and promoting metastasis [33]. Furthermore, other studies have observed that the increase in GRP78 can promote the expression of OCT4 in head and neck and breast cancer cells [34, 35]. Hence, it is worth considering that the increase in ER stress may account for the lack of reduction in these genes.

Tumor cells commonly experience ER stress due to various stress sources [36]. While no established link between HOOK1 and ER stress exists, we can speculate on potential causes. The protein HOOK2, from the same family as HOOK1, is involved in the formation and maintenance of aggresomes [37], structures whose inhibition has been associated with accumulation of misfolded proteins and ER stress [38, 39]. HOOK1 might play a similar role in the formation of this structure, possibly through the transport of misfolded proteins to this structure due to its role in retrograde transport. Notably, there are structural and functional similarities between the HDAC6 protein, which is essential for aggresome formation, and the HOOK protein family [11, 38]. Additionally, the FHF complex (HOOK, FTS, and FHIP) has been associated with the motility of tubular intermediaries that allow ER transport to the Golgi apparatus [14]. Hindered transport between these two organelles could also lead to increased ER stress.

Independently from its origin, increased ER stress could be responsible for the elevation in cell death observed in cells with downregulated HOOK1. Sustained ER stress is cytotoxic to cells, leading to the activation of the apoptotic pathway and other cell death mechanisms [40, 41]. We found that reducing ER stress in our model partially reverses cell death, indicating that ER stress may be responsible, at least in part, for this death. However, we did not observe a complete reversal of the phenotype. This could be explained because the inhibition of ER stress was not sufficiently effective, although we did observe a reduction in ER stress markers when using TUDCA. Therefore, it is possible that cells with reduced HOOK1 levels experience increased cell death due to more than one factor.

In addition to UPR activation, cells with reduced HOOK1 levels show changes in autophagic flux. In recent years, a strong interconnection between the UPR and autophagy has been described [31]. The role of autophagy in cancer is complex and context dependent. Its function in maintaining homeostasis plays an important role in preventing tumorigenesis. However, autophagic induction in tumors exposed to hypoxia and/or nutrient deprivation can provide the necessary energy to promote cell survival and chemoresistance [36]. In contrast, excessive autophagic activation can also trigger cell death through different mechanisms [42]. Therefore, the exact role of autophagy in cancer is dependent on tumor type, stage, and tumor microenvironmental conditions. In cells with reduced HOOK1 levels, UPR activation would be expected to increase autophagic flux. However, our results suggest that the decrease in HOOK1 could cause some kind of blockage in this process, that would prevent autophagosome-lysosome fusion, leading to an accumulation of these structures.

This partial blockage of autophagy could be related to the interaction of the FHF complex, including HOOK proteins, with the HOPS complex. It has been suggested that the FHF complex may help coordinate the movement or interaction of vesicles through the HOPS complex [13], which is involved in the fusion of late endosomes and autophagosomes with lysosomes in multiple organisms. Therefore, the loss of any of its subunits leads to the accumulation of late endosomes and autophagosomes [43–45]. Thus, it is possible that HOOK1 loss blocks autophagic flux by interfering with the proper functioning of the HOPS complex. Studying the interaction of HOOK1 with the HOPS complex in the future could help elucidate why autophagic flux might be partially impeded in our model.

Both excessive activation and blockade of autophagy can lead to cell death through apoptotic signaling in tumor cells [46, 47]. However, the ability of autophagy to suppress necrotic death is considered one of its most important prosurvival mechanisms [48, 49], although other studies have shown that autophagy can promote necroptosis [50, 51]. Therefore, the involvement of autophagy in cell survival or death is complex and highly dependent on the cellular context. Generally, an increase in ER stress induces a protective increase in autophagic flux to reduce proteotoxic stress and avoid cell death. Our results show that blocking autophagic flux affect cells with reduced HOOK1 levels to a greater extent than parental cells, leading to a significant increase in cell death, especially necrosis. This finding correlates with the role of autophagy as a suppressor of necrotic death and suggests that the autophagic pathway may act as a protective mechanism against ER stress in our model. However, this prosurvival mechanism might not function perfectly, as the decrease in HOOK1 also appears to cause a partial blockade of autophagic flux. Therefore, it may not be as effective as expected in preventing cell death.

The occurrence of ER stress could also explain the changes observed in terms of CSC properties when HOOK1 is reduced. We observed that the downregulation of this gene led to an increase in the death of EpCAM + cells in the ovarian cancer cell lines studied. This result suggests that the reduction in HOOK1, not only causes a decrease in the survival of ovarian cancer cells in general, but also specifically affects CSCs. Numerous studies have shown that maintaining ER proteostasis intact is crucial for maintaining CSC integrity. For example, UPR induction can decrease CSC properties in colorectal carcinoma [52, 53], breast cancer [54], head and neck cancer [55], glioblastoma [56, 57], and prostate carcinoma [58]. We observed that increased ER stress can reproduce the cell death of CSCs observed when HOOK1 is downregulated. This finding further supports the hypothesis that the decrease in stem cell properties when HOOK1 is reduced may be due to this phenomenon. The partial blockade of autophagy could also impact the survival of CSCs. Autophagy has been linked to the maintenance of CSCs in different tumors, such as breast cancer [59, 60], pancreatic and liver carcinoma [61], osteosarcoma [62], ovarian cancer [63], head and neck cancer [64], and glioblastoma [65]. Consistent with these studies, we have observed that blocking autophagic flux with chloroquine also increases CSC death and reduces their proportion within the tumor cell culture.

Given that reducing HOOK1 levels leads to the occurrence of proteotoxic stress and compromises the survival of CSCs, inhibiting this protein could be a promising therapeutic strategy in ovarian tumors. In fact, we observed that the decrease in HOOK1 sensitizes cells to compounds inducing ER stress and to proteasome inhibitors and that inhibiting HOOK1 prevented tumor formation in mouse models. Drugs capable of inducing ER stress or blocking autophagy have been widely used in studies to treat different types of tumors. For ER stress, preclinical studies suggest the utility of potent activation of this mechanism to kill tumor cells [66–69]. In the case of autophagy, numerous studies have described that blocking this pathway can produce a cytotoxic effect in tumor cells. Two drugs that block the autophagic flux have been approved for clinical use, chloroquine and its derivative hydroxychloroquine (HCQ), and they have been used in clinical trials of adenocarcinoma, melanoma, colorectal carcinoma, myeloma, lymphoma, and renal carcinoma [70]. Hence, autophagy blockade is currently considered a potential strategy in the treatment of tumors refractory to conventional therapies. Based on the described use of drugs affecting proteostasis as therapeutic strategies in cancer, HOOK1 could be a promising therapeutic target, since reducing this protein affects multiple cellular processes capable of triggering proteotoxic stress in tumor cells. Therefore, it would be interesting to study HOOK1 inhibition as a potential treatment for ovarian cancer, either as monotherapy or in combination with chemotherapeutic agents or other drugs inducing proteotoxic stress, such as inhibitors of the proteasome or promoters of the UPR response.

## Methods

### Cell culture

SKOV-3 and OVCAR-8 cell lines were obtained from the ATCC commercial repository and maintained in RPMI (AQmedia: Sigma) with 10% fetal bovine serum (FBS) (Gibco), penicillin, streptomycin and fungizone (Sigma) and incubated at 37 °C in 5% CO2 in a humidified atmosphere. Cells were negative for mycoplasma.

### CRISPR/Cas9 generation of HOOK1 knockdown

A sgRNA targeting the HOOK1 sequence TCTGAATGACCTTCGCAAGC (exon 11) was used to generate knockdown models. First, we infected the cells with virus containing the sgRNA and selected them with puromycin (1 µg/ml). Then, the cells were isolated by single-cell sorting by FACS Jazz (BD Biosciences) in 96-well plates. One month later, samples from each well that grew were amplified and validated by Western-Blot analysis. The selected CRISPRs were sequenced by the *Genomics and Sequencing* service at IBiS.

### Growth curve

For measurement of the proliferative capacity, 5 × 10^4^ cells were seeded in 12-well plates in triplicate. At 24 h (Day 0), cells were fixed with 0.5% glutaraldehyde (Sigma), and every 24/48 h, a curve point was fixed for up to 9 days. Once all the points were collected, the plates were stained with 0.5% crystal violet (Sigma). Then, crystal violet was solubilized in 20% acetic acid (Sigma) and quantified at 595 nm absorbance as a relative measure of cell number. The values are presented relative to Day 0.

### Migration assay (Boyden chamber)

A total of 9 × 10^5^ (OVCAR-8) or 4 × 10^5^ (SKOV-3) cells were resuspended in FBS-free medium and seeded in an 8 μm Boyden chamber (Transwell). The chamber was placed in a 24-well plate with FBS medium. At 24 h, the cells were fixed with 0.5% glutaraldehyde and stained with 0.5% crystal violet. The inner membrane of the chamber was cleaned to prevent non-migrated cells being stained. Pictures were taken at 20x, and migrated cells were counted using an inverted microscope (Olympus IX-71).

### Cytotoxicity assay

A total of 1.2 × 10^4^ cells/well were seeded in triplicate in a 96-well plate. The next day, the cells were treated with decreasing concentrations of the compounds brefeldin A (10 − 0 µM), thapsigargin (1 − 0 µM), tunicamycin (100-0 µM), bortezomib (1 − 0 µM) and MG-132 (100-0 µM). After 96 h, the cells were stained with 0.5% crystal violet. Then, crystal violet was solubilized in 20% acetic acid (Sigma) and quantified at 595 nm absorbance to measure cell viability.

### Clonogenic assay and clonal heterogeneity analysis

For measurement of the ability of cells to form individual clones, 1 × 10^4^ cells were plated in 10 cm plates in triplicate. Cells were fixed with 0.5% glutaraldehyde and stained with 0.5% crystal violet after 10 days. The number of clones was counted, and the types of clones were classified according to phenotype and ability to reconstitute the culture.

### Tumorsphere assay

A total of 3 × 10^4^ cells were seeded in triplicate in 24-well Ultra-Low Attachment Plates (Costar) containing 1 mL of MammoCult basal medium (Stem Cell Technologies) supplied with 10% MammoCult proliferative supplement, 4 µg/mL heparin, 0.48 µg/mL hydrocortisone, penicillin and streptomycin. After 7 days, the number of primary tumorspheres formed was measured using an inverted microscope (Olympus IX-71).

### Cell death assay

The cells were maintained in culture with or without treatment for 48–72 hours (Table S1). Then, both suspended cells and adherent cells were collected, and the ‘Apoptosis Detection’ kit (Immunostep) was used to measure cell death following the manufacturer’s instructions. Briefly, cells were stained with Annexin V for 15 min at room temperature and in darkness. Subsequently, they were washed with Binding Buffer solution and centrifuged to remove the supernatant. Finally, they were incubated with propidium iodide for 5 min at room temperature. A FACSCanto II flow cytometer (BD Biosciences) was employed to detect the staining, and the results were analyzed using Diva software. Cells stained only with Annexin V were considered in early apoptosis, cells stained only with propidium iodide were considered in necrosis, and cells stained with both markers were considered in late apoptosis. All cells stained with propidium iodide were considered dead (Supp. Figure 11).

### Cell death assay in cells labeled with EpCAM by flow cytometry

The cells were maintained in culture with or without treatment for 48–72 h (Table S1). Then, both suspended and adherent cells were collected. 1 × 10^6^ of those cells were resuspended in PBS with 2% FBS and 5 mM EDTA. Next, the cells were incubated with blocking agent (Miltenyi Biotec) for 10 min at 4 °C, and then, antibody labeling (Miltenyi Biotec) was performed for 15 min at room temperature and 15 min at 4 °C. Following this, two washes were performed using PBS with 2% FBS and 5 mM EDTA. After that, the previously described kit (Inmunostep) was used to label dead cells. Finally, the cells were examined using a FACSCanto II flow cytometer (BD Biosciences), and the results were analyzed using Diva software. The number of dead cells was measured in EpCAM + cells and the whole population. All cells stained with propidium iodide were considered dead.

### Xenograft in nude mice

Tumorigenicity was assayed by the subcutaneous injection of 4 × 10^6^ (OVCAR-8) or 5 × 10^6^ (SKOV-3) cells into the right flanks of four 4-week-old female athymic nude mice. Cells were suspended in Matrigel (Corning) prior to the injection. Animals were examined weekly. After 80–120 days, depending on the cell lines, mice were sacrificed, and tumors were extracted and conserved at -80 °C. Tumor volume (mm^3^) was measured using calipers. All animal experiments were performed according to the experimental protocol approved by the IBIS and HUVR Institutional Animal Care and Use Committee (0309-N-15).

### RT‒qPCR

Total RNA from cell lines was extracted and purified using the ReliaPrepTM RNA Tissue Miniprep System (Promega), and reverse transcription was performed using the High Capacity cDNA Reverse Transcription kit (Life Technologies) according to the manufacturer’s instructions. The qPCR mixture contained the reverse transcriptase reaction product (16,6 ng/µl), 2.5 µL of water, 5 µL of GoTaqR Probe qPCR Master Mix (Promega) and 0.5 µL of the appropriate TaqMan Assay (Applied Biosystems/IDT). The following probes were used: HPRT1 (Hs.PT.58.v.45,621,572) as an endogenous control and HOOK1 (Hs.PT.58.3256265), NANOG (Hs04260366_g1), OCT4 (Hs00999632_g1), KLF4 (Hs00358836_m1) and MYC (Hs00153408_m1).

### Protein isolation and Western blot analysis

Western blotting was performed according to standard procedures. We used the following primary antibodies: anti-HOOK1 (Abcam, ab151756), anti-CASP3 (Cell Signaling, #9664), anti-CASP9 (Cell Signaling, #9502), anti-PARP (Cell Signaling, #9532), anti-p-H2AX (Ser139) (Cell Signaling, #9718), anti-NANOG (Santa Cruz Biotechnology, sc-293,121), anti-OCT3/4 (Santa Cruz Biotechnology, sc-5279), anti-KLF4 (Abcam, ab72543), anti-ATF6α (Santa Cruz Biotechnology, sc-166,659), anti-ATF4 (Santa Cruz Biotechnology, sc-390,063), anti-GRP78 (Santa Cruz Biotechnology, sc-13,539), anti-CHOP (Cell Signaling, #2895), anti-LC3B (Abcam, ab48394), anti-p62 (Abcam, ab109012), and anti-B-actin (Abcam, ab16039) as a loading control. We used the following secondary antibodies: rabbit anti-mouse (Abcam, ab97046) and goat anti-rabbit (Abcam, ab97051). The proteins were detected using an ECL detection system (Amersham Biosciences) and a Bio-Rad Chemidoc Touch.

### Proteomic analysis

**Gel electrophoresis and in-gel digestion of proteins.** Gel electrophoresis and in-gel digestion were performed as described elsewhere [71]. Briefly, protein lysates were separated on precast 4–12% gradient gels using the NuPAGE SDS-PAGE system (Invitrogen, Carlsbad, CA). Following electrophoresis, gels were fixed in 50% ethanol/3% phosphoric acid solution and stained with Coomassie R-250. Subsequently, the gels were washed and proteins reduced and alkylated by incubating the whole gel in dithiothreitol and iodoacetamide, respectively. Gel lanes were cut into 5 bands and each band was cut into ∼ 1 mm^3^ cubes. The gel cubes from one band were transferred into an eppendorf tube and incubated with trypsin o/n. The peptides from each gel band were extracted and stored at − 20 °C until LC-MS/MS analysis.

**LC-MS/MS**. Peptides were separated by an Ultimate 3000 nanoLC‐MS/MS system (Dionex LC‐Packings) equipped with a 45 cm × 75 μm ID fused silica column custom packed with 1.9 μm 120 Å ReproSil Pur C18 aqua (Dr Maisch GMBH). After injection, peptides were trapped at 6 µl/min on a 10 mm ×100 μm ID trap column packed with 5 μm 120 Å ReproSil Pur C18 aqua in 0.05% formic acid. Peptides were separated at 300 nl/min in a 10–40% gradient (buffer A: 0.5% acetic acid (Fisher Scientific), buffer B: 80% ACN, 0.5% acetic acid) in 60 min (100‐min inject‐to‐inject). Eluting peptides were ionized at a potential of + 2 kVa into a Q Exactive mass spectrometer (Thermo Fisher). Intact masses were measured at resolution 70,000 (at m/z 200) in the orbitrap using an AGC target value of 3E6 charges. The top 10 peptide signals (charge‐states 2 + and higher) were submitted to MS/MS in the HCD (higher‐energy collision) cell (1.6 amu isolation width, 25% normalized collision energy). MS/MS spectra were acquired at resolution 17,500 (at m/z 200) in the orbitrap using an AGC target value of 1E6 charges, a maxIT of 60 ms, and an underfill ratio of 0.1%. Dynamic exclusion was applied with a repeat count of 1 and an exclusion time of 30 s.

**Protein identification and label-free quantitation.** MS/MS spectra were searched against the reference proteome FASTA file (42,161 entries; swissprot_2017_03_human_canonical_and_isoform). Enzyme specificity was set to trypsin, and up to two missed cleavages were allowed. Cysteine carboxamidomethylation (Cys, + 57.021464 Da) was treated as fixed modification and methionine oxidation (Met, + 15.994915 Da) and N‐terminal acetylation (N‐terminal, + 42.010565 Da) as variable modifications. Peptide precursor ions were searched with a maximum mass deviation of 4.5 ppm and fragment ions with a maximum mass deviation of 20 ppm. Peptide and protein identifications were filtered at an FDR of 1% using the decoy database strategy. The minimal peptide length was seven amino acids. Proteins that could not be differentiated based on MS/MS spectra alone were grouped into protein groups (default MaxQuant settings). Searches were performed with the label‐free quantification option selected. Proteins were quantified by spectral counting, that is, the number of identified MS/MS spectra for a given protein [72] combining the five fractions per sample. Raw counts were normalized on the sum of spectral counts for all identified proteins in a particular sample, relative to the average sample sum determined with all samples. To find statistically significant differences in normalized counts between sample groups, we applied the beta‐binomial test [73], which takes into account within‐sample and between‐sample variation using an alpha level of 0.05. The mass spectrometry proteomics data have been deposited to the ProteomeXchange Consortium via the PRIDE [74] partner repository with the dataset identifier PXD050782. The generated data were filtered using the R platform [75] to obtain proteins whose expression increased (FC > 1) or decreased (FC < -1) significantly (*p* < 0.05) and analyzed using the *ShinyGO 0.77* [76] (http://bioinformatics.sdstate.edu/go/) and *STRING* (https://string-db.org/) [77] platforms.

### Colocalization assays

Cells were seeded onto glass coverslips, fixed with 4% paraformaldehyde for 20 min and permeabilized with 0.5% Triton X-100 for 5 min. The coverslips were incubated with blocking solution (PBS + 0.1% Triton X-100 + 3% BSA) for 1 h and then incubated with anti-LAMP2 antibody (1:250) overnight at 4 °C. The coverslips were washed four times with PBS + 0.1% Triton X-100 and incubated overnight at 4 °C with the second primary antibody, anti-LC3B (1:250). The secondary antibodies anti-rabbit Alexa Fluor 488 (1:250, Thermo Fisher A-11,008) and anti-mouse Alexa Fluor 633 (1:250, Thermo Fisher A-21,052) were used. The nuclei were stained with DAPI, and the coverslips were mounted with ProLong Gold Antifade (Life Technologies). A confocal ultraspectral microscope (Leica Stellaris 8) that allowed sequential scanning of emission channels was used for image detection, and the images were analyzed with *ImageJ/Fiji* software [78, 79].

### Public database analysis

The following public databases were employed in this article: cBioPortal (http://www.cbioportal.org/) [80], GEPIA (http://gepia.cancer-pku.cn/) [81] and R2: Genomics Analysis and Visualization Platform (http://r2.amc.nl), which were employed to gather information on tumors from ovarian cancer patients.

### Statistical analysis

Statistical analyses of experiments were performed using GraphPad Prism. Control samples and CRISPR clones were compared using unpaired Student’s t test or Student’s t test with Welch’s correction, as appropriate. Experiments were performed a minimum of three times independently and in triplicate samples. p values less than 0.05 were considered statistically significant and were represented according to the following classification: *p* < 0.05 (*), *p* < 0.01 (**), and *p* < 0.001 (***).

### Electronic supplementary material

Below is the link to the electronic supplementary material.

Supplementary Material 1

Supplementary Material 2

## Data Availability

All data and material will be available upon reasonable request.

## Abbreviations

Bref. A: Brefeldin A
CIE: Clathrin-independent endocytosis
CSCs: Cancer stem cells
ER: Endoplasmic reticulum
EMT: Epithelial-mesenchymal transition
FC: Fold-change
HCQ: Hydroxychloroquine
HOPS complex: Homotypic fusion and protein sorting complex
IC50: Half-maximal inhibitory concentration
RT-qPCR: Reverse transcription-quantitative PCR
sgRNA: Single guide-RNA
TUDCA: Tauroursodeoxycholic acid
UPR: Unfolded protein response
WB: Western blot
