# Supplementary material for: Protein homeostasis maintained by HOOK1 levels promotes the tumorigenic and stemness properties of ovarian cancer cells through reticulum stress and autophagy
Source: J Exp Clin Cancer Res. 2024 May 29;43:150. doi: 10.1186/s13046-024-03071-2 (PMC11134651; doi:10.1186/s13046-024-03071-2)
Supplement: Supplementary file 1 — Supplementary Material 1 [file 13046_2024_3071_MOESM1_ESM.pdf]

Supplementary Figures

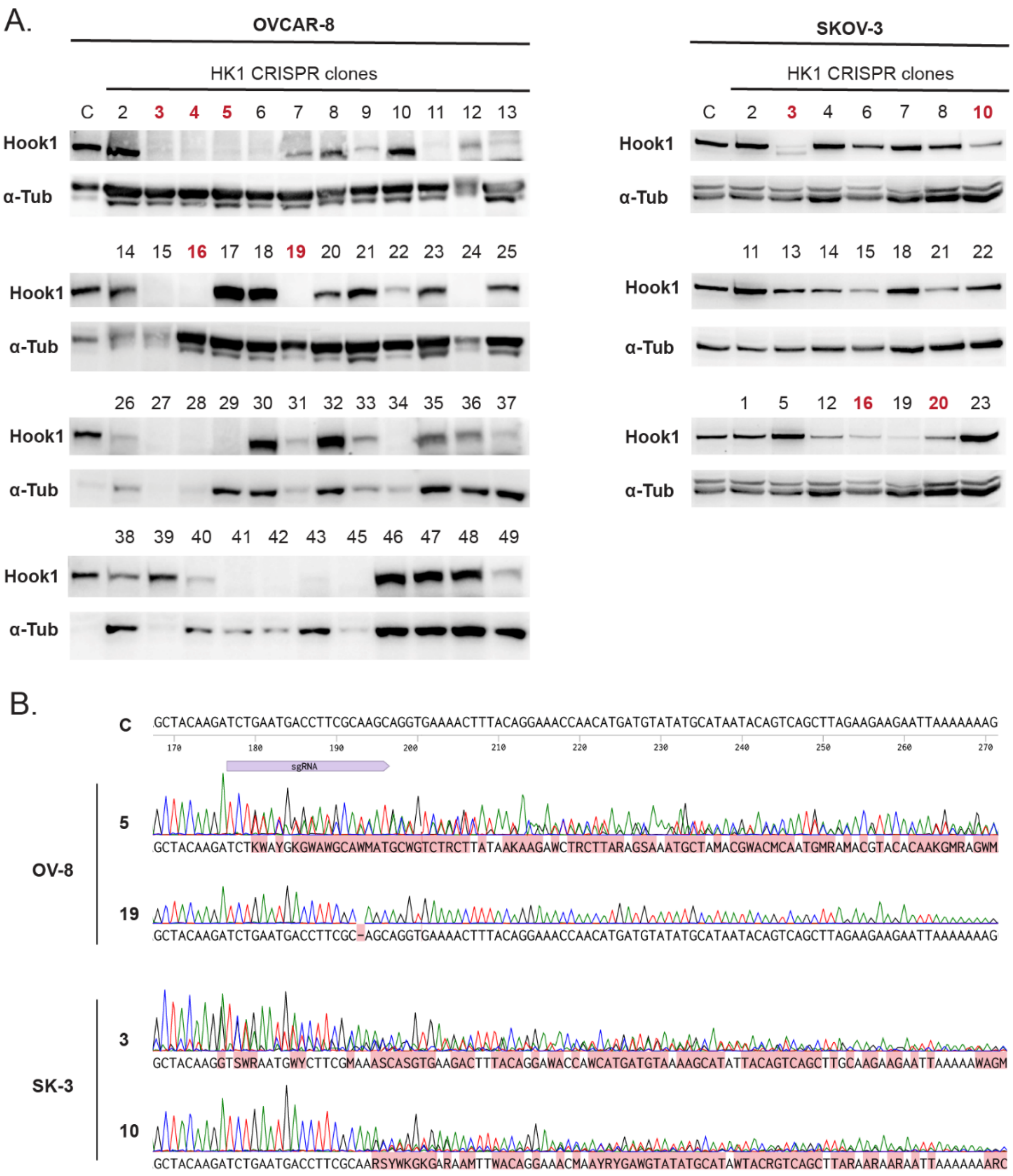

Supplementary Figure 1. Generation and validation of HOOK1 CRISPRs in ovarian cancer cell lines. (A) Protein levels of HOOK1 after CRISPR–Cas9 system infection in ovarian cancer cell lines. The selected clones are highlighted in red. **(B)** DNA sequencing of the sgRNA region of clones 5 and 19 of OVCAR-8 CRISPR and clones 3 and 10 of SKOV-3 CRISPR compared to the original sequence.

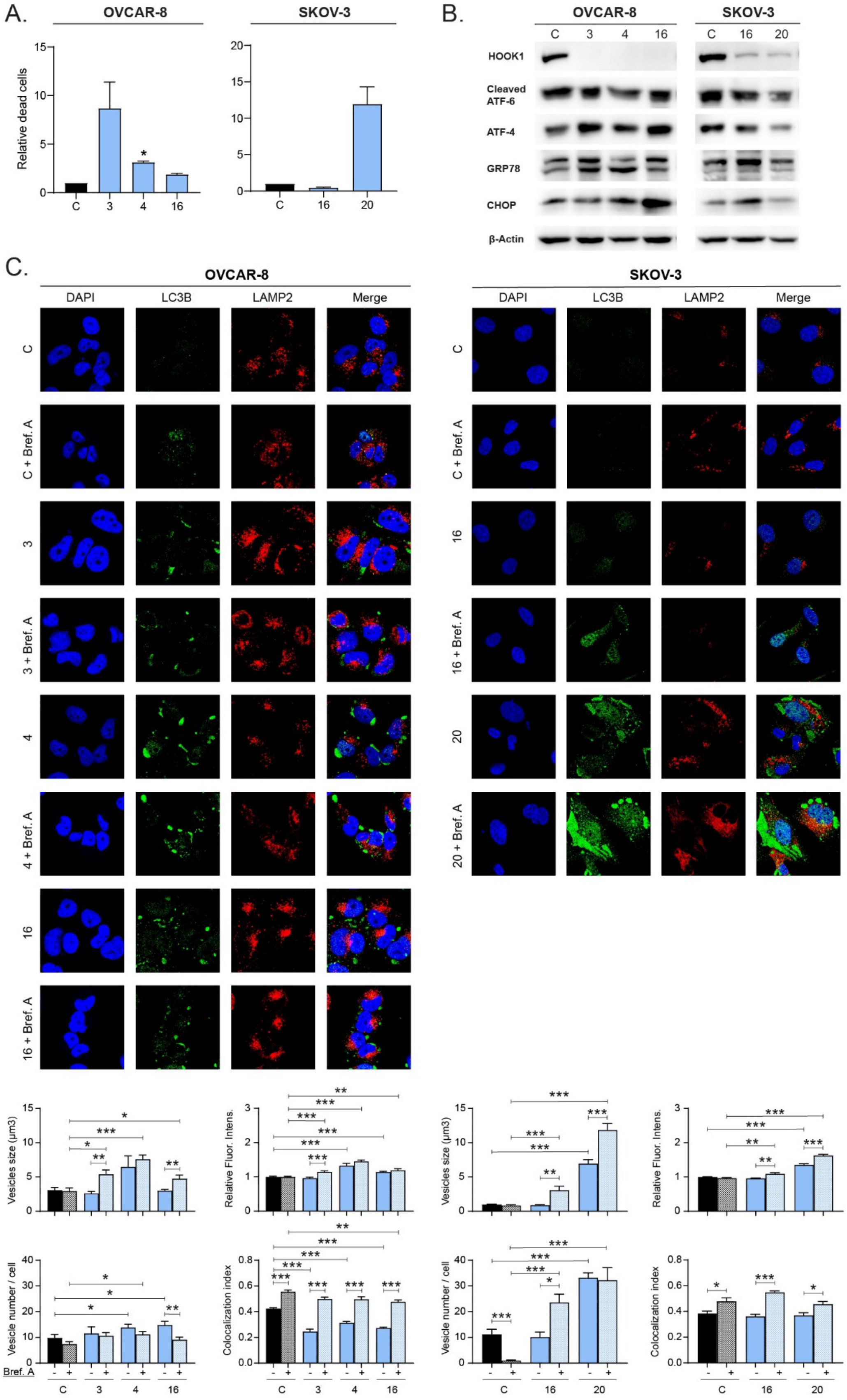
A.
OVCAR-8
SKOV-3
Relative dead cells
C
3
4
16
20
B.
OVCAR-8
SKOV-3
HOOK1
Cleaved ATF-6
ATF-4
GRP78
CHOP
β-Actin
C.
DAPI
LC3B
LAMP2
Merge
C + Bref. A
3 + Bref. A
4 + Bref. A
16 + Bref. A
20 + Bref. A
Vesicles size (μm3)
Relative Fluor. Intens.
Vesicle number / cell
Colocalization index
Bref. A

Supplementary Figure 2. Analysis of cell death, ER stress and autophagy in different HOOK1 CRISPR clones in OVCAR-8 and SKOV-3 cell lines. (A) Quantification by flow cytometry of the relative number of dead cells when Hook1 is downregulated. **(B)** Protein levels of UPR-associated proteins in Hook1-downregulated cells. **(C)** Immunofluorescence staining of LC3B (green) and LAMP2 (red) proteins in Hook1-downregulated cells treated with brefeldin A. DAPI (blue) was used as a nuclear stain, and the merge of the 3 markers is shown. The autophagosome size, number and relative fluorescence intensity, as well as the colocalization index of autophagosomes-lysosomes, were measured using ImageJ software. The statistical analysis was performed using Student's t test (*$p<0.05$; **$p<0.01$; ***$p<0.001$).

A.

OVCAR-8

Process	Go term	Associated genes
**Protein folding**	Protein folding in endoplasmic reticulum	HSPA5 HSP90B1 PDIA3 CALR
	ATF6-mediated unfolded protein response	HSPA5 HSP90B1 CALR
	ER unfolded protein response/ Cellular response to unfolded protein	HSPA5 PDIA6 LMNA HSP90B1 CALR GFPT1
	Cellular response to topologically incorrect protein	HSPA5 UGGT1 PDIA6 LMNA HSP90B1 CALR GFPT1
	Response to unfolded protein	HSPA5 PDIA6 MANF LMNA HSP90B1 CALR GFPT1
	Response to topologically incorrect protein	HSPA5 UGGT1 PDIA6 MANF LMNA HSP90B1 CALR GFPT1
	Response to ER stress	HSPA5 UGGT1 PDIA6 MANF PDIA4 LMNA HSP90B1 PDIA3 CALR GFPT1
	Protein folding	HSPA5 UGGT1 PDIA6 PDIA4 HSP90B1 PDIA3 CALR
**Metabolic process**	UDP-N-acetylglucosamine/ amino sugar biosynthetic process	PGM3 AMDHD2 GFPT1
	L-ascorbic acid/ lactone metabolic process	SLC2A3 SLC2A1 AKR1A1
	Nucleotide-sugar metabolic process	PGM3 UGGT1 AMDHD2 GFPT1
**Cytoskeleton organization**	Negative regulation of supramolecular fiber organization	CAPG LIMA1 ARHGEF2 LDLR HIP1R GSN
	Actin cytoskeleton organization/ Actin filament-based process	CAPG LIMA1 ACTN1 ICAM1 ARHGEF2 PALLD HIP1R RAB13 GSN HSP90B1 CALR
	Cytoskeleton organization	CAPG LIMA1 ACTN1 ICAM1 CNTNAP1 ARHGEF2 CENPF PALLD CLIP1 HIP1R RAB13 GSN LMNA HSP90B1 CALR
**Others**	Response to organonitrogen compound	CD9 HSPA5 CYBA ICAM1 ARHGEF2 SLC2A1 LDLR UGGT1 USO1 RAB13 GSN RGS10 HSP90B1
	Response to oxygen-containing compound	CD9 HSPA5 CYBA ICAM1 MTAP ARHGEF2 SLC2A1 AKR1A1 LDLR USO1 PFKL RAB13 GSN RGS10 HSP90B1 CALR SMYD3

SKOV-3

Process	Go term	Associated genes
**Migration**	Mesenchyme migration	ACTA2 ACTA1 ACTC1 ACTG2
	Tissue migration	ANLN HSPB1 ACTA2 ANXA1 ACTA1 ACTC1 ACTG2 ITGB3
**Protein folding**	Protein folding in endoplasmic reticulum	HSPA5 HSP90B1 P4HB
**Cytoskeleton organization**	Neg. Reg. of actin filament depolymerization	LIMA1 SPTBN1 GSN SPTAN1
	Actin filament-based movement	VIM ACTA1 GSN MYO1E ACTC1 FLNA
	Actin filament organization	LIMA1 PDLIM1 SPTBN1 ACTA1 GSN MYO1E ACTC1 HSP90B1 FLNA SPTAN1 CD2AP
	Actin cytoskeleton organization	ANLN LIMA1 PDLIM1 SPTBN1 ANXA1 SDCBP ACTA1 GSN MYO1E ACTC1 HSP90B1 FLNA SPTAN1 CD2AP
	Actin filament-based process	ANLN VIM LIMA1 PDLIM1 SPTBN1 ANXA1 SDCBP ACTA1 GSN MYO1E ACTC1 HSP90B1 FLNA SPTAN1 CD2AP
	Supramolecular fiber organization	VIM LIMA1 PDLIM1 SPTBN1 ACTA1 GSN MYO1E ACTC1 HSP90B1 P4HB FLNA SPTAN1 CD2AP
	Cytoskeleton organization	ANLN VIM LIMA1 PDLIM1 CCDC6 GAPDH SPTBN1 CDC20 CCNB1 ANXA1 SDCBP ACTA1 GSN MYO1E ACTC1 HSP90B1 FLNA SPTAN1 CD2AP
**Secretion**	Exocytosis	VCL HUWE1 CPPED1 ANXA1 SDCBP GSN TAGLN2 PGAM1 FLNA IGF2R SPTAN1 ITGB3
	Secretion by cell	VCL HUWE1 CPPED1 OSBP ANXA1 SDCBP GSN PDIA4 TAGLN2 PGAM1 FLNA IGF2R SPTAN1 CD2AP ITGB3
	Muscle system process	VIM VCL ENO1 ACTA2 ACTA1 GSN ACTC1 ACTG2 FLNA
**Others**	Neg. reg. of cellular component organization	VIM HSPA5 LIMA1 HUWE1 SPTBN1 CDC20 TOP2A CCNB1 SDCBP GSN FLNA SPTAN1 ITGB3
	Reg. of organelle organization	LIMA1 ENO1 HUWE1 OSBP SPTBN1 CDC20 TOP2A CCNB1 ANXA1 SDCBP GSN FLNA SPTAN1 CD2AP

B.

OVCAR-8

Process	Go term	Associated genes
**Regulation of RNA**	RNA splicing	DDX5 HNRNPH3 GEMIN5 U2AF2 GEMIN4 HNRNPH1 PTBP2 PUF60
	ncRNA metabolic process	GLTSCR2 DDX21 PRKDC GEMIN4 RRS1 GTPBP4 LARS TARS RRP12
	Nucleotide metabolic process	RRM1 PKM NT5C2 MCCC2 PGM1 PAPSS2 HSD17B4 NNMT
	RNA processing	DDX5 GLTSCR2 DDX21 HNRNPH3 GEMIN5 U2AF2 PRKDC GEMIN4 RRS1 HNRNPH1 GTPBP4 PTBP2 PUF60 RRP12
	RNA metabolic process	TRIP13 DDX5 GLTSCR2 NOTCH2 DDX21 POLR1A HNRNPH3 GEMIN5 U2AF2 PRKDC GEMIN4 RRS1 HNRNPH1 TTC37 GTPBP4 PTBP2 TCOF1 LARS TARS PUF60 RRP12 GTF2I DDX24 CSDE1
**Formation of protein complex**	Ribonucleoprotein complex assembly	GLTSCR2 GEMIN5 PRKDC GEMIN4 RRS1 PTBP2 PUF60
	Ribonucleoprotein complex biogenesis	GLTSCR2 DDX21 GEMIN5 PRKDC GEMIN4 RRS1 GTPBP4 PTBP2 PUF60 RRP12
	Protein-containing complex assembly	GLTSCR2 GEMIN5 RRM1 MAT2A PRKDC BIN1 GEMIN4 RRS1 DNAJB12 FN1 PTBP2 ATL2 DIAPH1 DIAPH3 PUF60 EHD1
**Regulation of translation**	rRNA processing /Ribosome biogenesis	GLTSCR2 DDX21 PRKDC GEMIN4 RRS1 GTPBP4 RRP12
**Others**	Pos, reg, of endocytic recycling	EHD2 EHD1
	Maintenance of dna methylation	DNMT1 UHRF1
	Response to nutrient	OXCT1 NNT AGL MAP1B PKM AACS

SKOV-3

Process	Go term	Associated genes
**Regulation of translation**	rRNA processing	FTSJ3 BRIX1 DDX54 DDX56 EIF4A3 RRP1B PRKDC
	Reg. of translation	NCL PUM1 THBS1 EIF4A3 ERBB2 APP LARP1 RBM4 PRKDC
	Ribosome biogenesis	FTSJ3 BRIX1 DDX54 DDX56 EIF4A3 RRP1B PRKDC
**Regulation of RNA**	ncRNA processing	FTSJ3 BRIX1 DDX54 DDX56 SSB EIF4A3 RRP1B PRKDC
	RNA splicing	NCL SMU1 EIF4A3 NONO RRP1B RBM4 HNRNPA0 AHNAK2
	Posttranscriptional reg. of gene expression	AAAS NCL PUM1 THBS1 EIF4A3 ERBB2 APP LARP1 RBM4 HNRNPA0 PRKDC
	RNA processing	FTSJ3 BRIX1 NCL SMU1 DDX54 PUM1 DDX56 SSB EIF4A3 APP NONO RRP1B RBM4 HNRNPA0 AHNAK2 PRKDC
	mRNA metabolic process	NCL SMU1 PUM1 SSB EIF4A3 APP NONO LARP1 RRP1B RBM4 HNRNPA0
	Neg. Reg. of gene expression	AAAS NCL PUM1 THBS1 ADAM10 SSB EIF4A3 APP LARP1 AXL RBM4 HNRNPA0 PRKDC
**Others**	Response to epidermal growth factor	NCL ERBB2 MCM7 BAIAP2
	Extracellular matrix organization	ITGA6 COL12A1 ITGB4 THBS1 ADAM10 APP SERPINH1 AGRN
	Reg. of cellular amide metabolic process	NCL PUM1 THBS1 EIF4A3 ERBB2 APP LARP1 RBM4 PRKDC
	Ribonucleoprotein complex biogenesis	HSP90AA1 FTSJ3 BRIX1 DDX54 DDX56 EIF4A3 RRP1B PRKDC
	Cell junction organization	ITGA6 ITGB4 THBS1 ADAM10 ERBB2 APP PTPRF BAIAP2 AGRN L1CAM
	Cellular component morphogenesis	HSP90AA1 ITGB4 ADAM10 ERBB2 APP DCDC2 KRT8 KRT19 BAIAP2 L1CAM
	Neurogenesis	HSP90AA1 ITGA6 ITGB4 DDX56 ADAM10 ERBB2 APP PTPRF DCDC2 AXL BAIAP2 AGRN L1CAM TGM2

Supplementary Figure 3. Biological processes associated with the proteins altered when HOOK1 is downregulated. Biological processes, GO terms and associated genes of **(A)** upregulated and **(B)** downregulated proteins in OVCAR-8 and SKOV-3 cell lines with downregulated HOOK1.

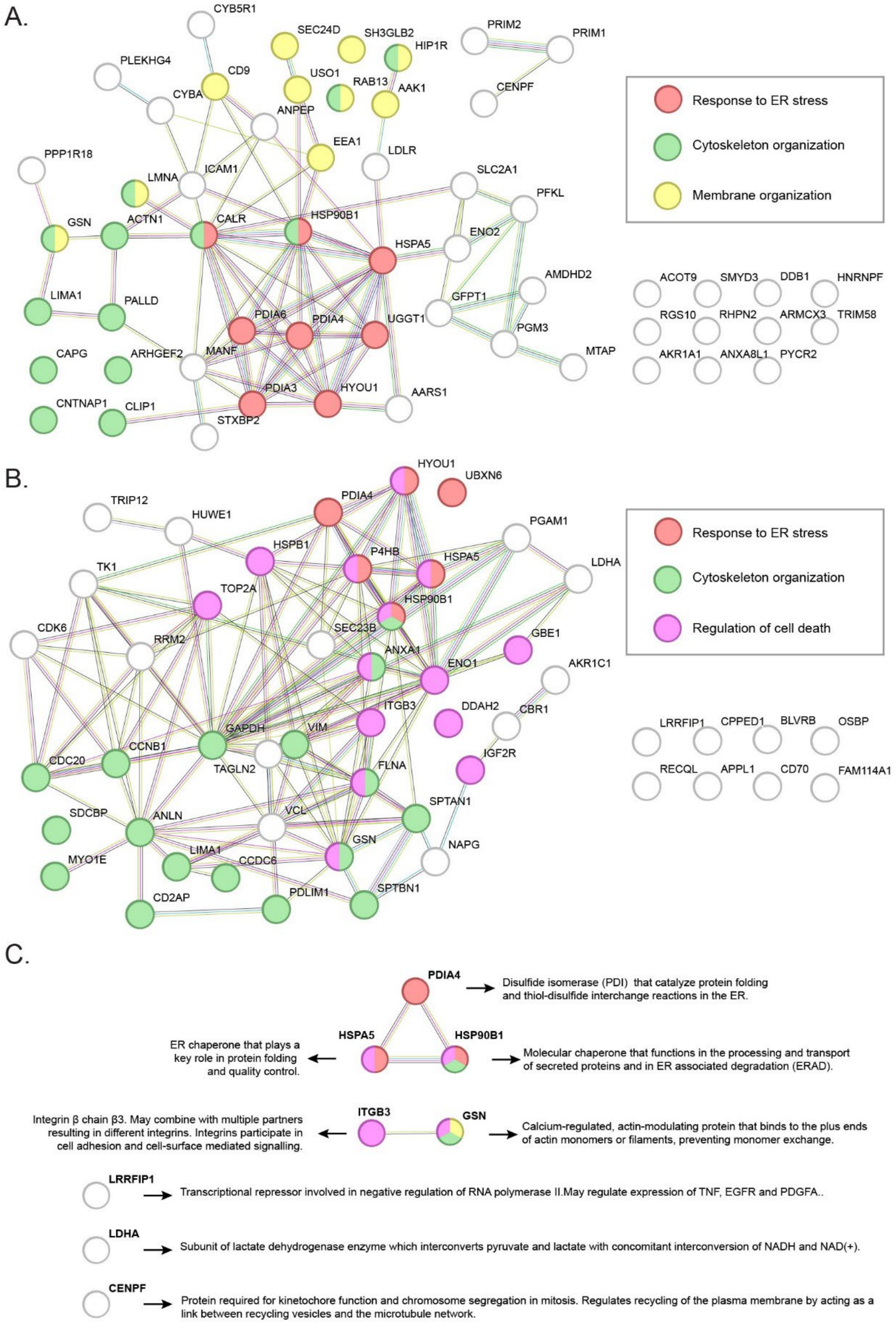
A.
CYB5R1
SEC24D
SH3GLB2
HIP1R
PRIM2
PRIM1
PLEKHG4
CD9
USO1
RAB13
AAK1
CENPF
CYBA
ANPEP
EEA1
LDLR
PPP1R18
LMNA
ICAM1
SLC2A1
PFKL
GSN
ACTN1
CALR
HSP90B1
HSPA5
ENO2
AMDHD2
LIMA1
PALLD
PDIA6
PDIA4
UGGT1
GFPT1
PGM3
CAPG
ARHGEF2
MANF
MTAP
PDIA3
HYOU1
AARS1
CNTNAP1
CLIP1
STXBP2
Response to ER stress
Cytoskeleton organization
Membrane organization
ACOT9
SMYD3
DDB1
HNRNPF
RGS10
RHPN2
ARMCX3
TRIM58
AKR1A1
ANXA8L1
PYCR2
B.
HYOU1
UBXN6
TRIP12
PDIA4
HUWE1
PGAM1
HSPB1
P4HB
HSPA5
LDHA
TK1
TOP2A
HSP90B1
CDK6
RRM2
SEC23B
GBE1
ANXA1
ENO1
AKR1C1
ITGB3
DDAH2
CBR1
GAPDH
VIM
CDC20
CCNB1
TAGLN2
IGF2R
FLNA
SPTAN1
SDCBP
ANLN
VCL
GSN
NAPG
MYO1E
LIMA1
CCDC6
CD2AP
PDLIM1
SPTBN1
Response to ER stress
Cytoskeleton organization
Regulation of cell death
LRRFIP1
CPPED1
BLVRB
OSBP
RECQL
APPL1
CD70
FAM114A1
C.
PDIA4
Disulfide isomerase (PDI) that catalyze protein folding and thiol-disulfide interchange reactions in the ER.
HSPA5
HSP90B1
ER chaperone that plays a key role in protein folding and quality control.
Molecular chaperone that functions in the processing and transport of secreted proteins and in ER associated degradation (ERAD).
ITGB3
GSN
Integrin β chain β3. May combine with multiple partners resulting in different integrins. Integrins participate in cell adhesion and cell-surface mediated signalling.
Calcium-regulated, actin-modulating protein that binds to the plus ends of actin monomers or filaments, preventing monomer exchange.
LRRFIP1
Transcriptional repressor involved in negative regulation of RNA polymerase II.May regulate expression of TNF, EGFR and PDGFA..
LDHA
Subunit of lactate dehydrogenase enzyme which interconverts pyruvate and lactate with concomitant interconversion of NADH and NAD(+).
CENPF
Protein required for kinetochore function and chromosome segregation in mitosis. Regulates recycling of the plasma membrane by acting as a link between recycling vesicles and the microtubule network.

Supplementary Figure 4. Analysis of upregulated proteins upon HOOK1 downregulation using STRING resource. Network of upregulated proteins in **(A)** OVCAR-8 and **(B)** SKOV-3 cell line. Network nodes represent proteins and edges represent protein-protein associations. Some genes are colored to show that they are associated to a specific GO term. **(C)** Network of the top 5 most significantly upregulated proteins in OVCAR-8 and SKOV-3 cell lines and brief description of their function within the cells.

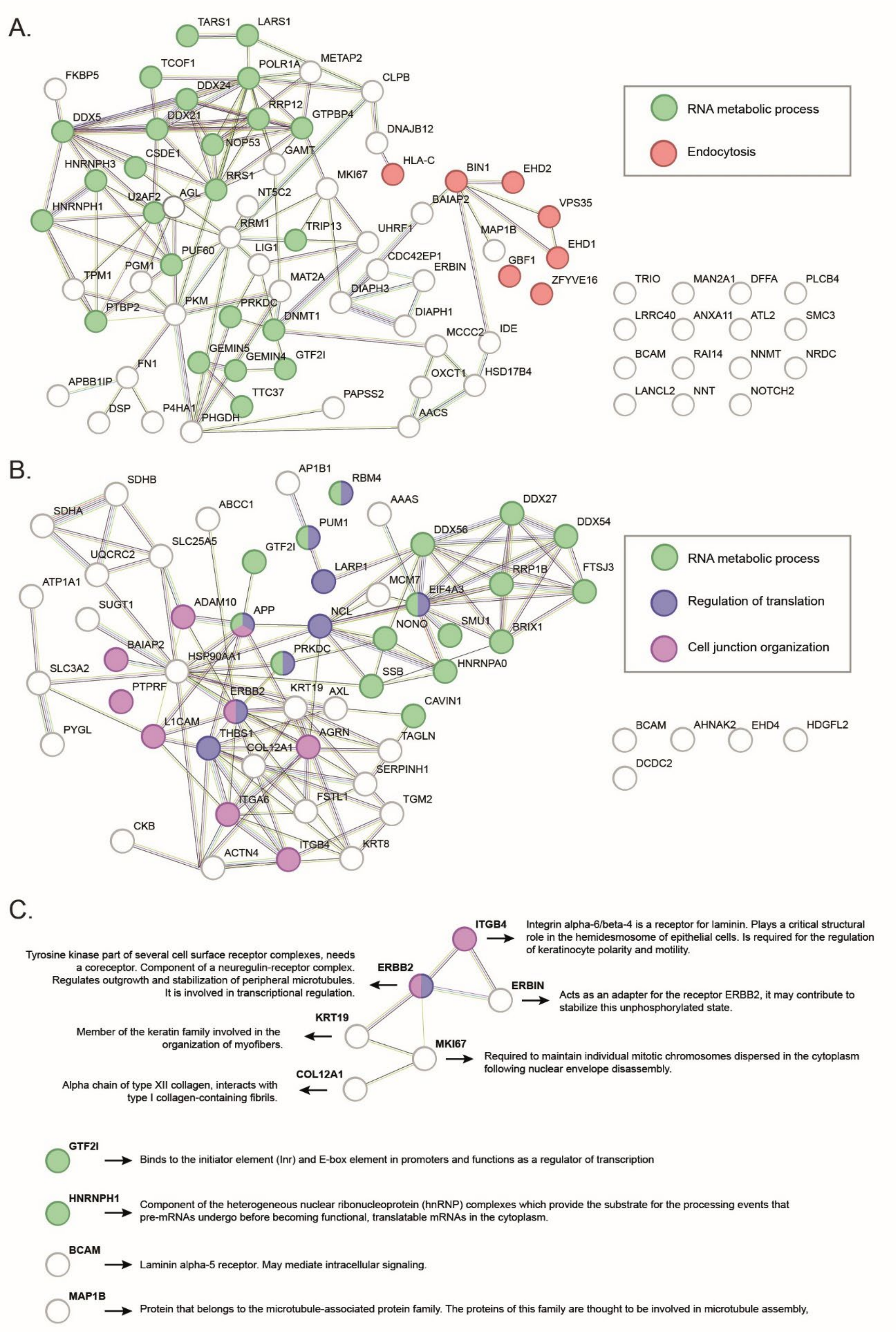
A.
TARS1
LARS1
TCOF1
POLR1A
METAP2
FKBP5
DDX24
CLPB
DDX5
DDX21
RRP12
GTPBP4
NOP53
GAMT
DNAJB12
CSDE1
HLA-C
HNRNPH3
RRS1
MKI67
BIN1
EHD2
U2AF2
AGL
NT5C2
HNRNPH1
BAIAP2
VPS35
RRM1
TRIP13
UHRF1
MAP1B
PUF60
LIG1
EHD1
PGM1
CDC42EP1
GBF1
TPM1
MAT2A
ERBIN
ZFYVE16
PTBP2
PKM
PRKDC
DIAPH3
DNMT1
DIAPH1
MCCC2
IDE
GEMIN5
GEMIN4
GTF2I
FN1
OXCT1
HSD17B4
APBB1IP
DSP
P4HA1
TTC37
PAPSS2
PHGDH
AACS
RNA metabolic process
Endocytosis
TRIO
MAN2A1
DFFA
PLCB4
LRRC40
ANXA11
ATL2
SMC3
BCAM
RAI14
NNMT
NRDC
LANCL2
NNT
NOTCH2
B.
AP1B1
RBM4
SDHB
SDHA
ABCC1
AAAS
DDX27
PUM1
DDX54
DDX56
SLC25A5
UQCRC2
GTF2I
LARP1
FTSJ3
RRP1B
MCM7
ATP1A1
EIF4A3
SUGT1
ADAM10
APP
NCL
NONO
SMU1
BRIX1
BAIAP2
PRKDC
HSP90AA1
HNRNPA0
SLC3A2
SSB
PTPRF
KRT19
ERBB2
AXL
CAVIN1
L1CAM
PYGL
THBS1
AGRN
TAGLN
COL12A1
SERPINH1
FSTL1
TGM2
ITGA6
CKB
ITGB4
KRT8
ACTN4
RNA metabolic process
Regulation of translation
Cell junction organization
BCAM
AHNAK2
EHD4
HDGFL2
DCDC2
C.
ITGB4
Integrin alpha-6/beta-4 is a receptor for laminin. Plays a critical structural role in the hemidesmosome of epithelial cells. Is required for the regulation of keratinocyte polarity and motility.
Tyrosine kinase part of several cell surface receptor complexes, needs a coreceptor. Component of a neuregulin-receptor complex. Regulates outgrowth and stabilization of peripheral microtubules. It is involved in transcriptional regulation.
ERBB2
ERBIN
Acts as an adapter for the receptor ERBB2, it may contribute to stabilize this unphosphorylated state.
KRT19
Member of the keratin family involved in the organization of myofibers.
MKI67
Required to maintain individual mitotic chromosomes dispersed in the cytoplasm following nuclear envelope disassembly.
COL12A1
Alpha chain of type XII collagen, interacts with type I collagen-containing fibrils.
GTF2I
Binds to the initiator element (Inr) and E-box element in promoters and functions as a regulator of transcription
HNRNPH1
Component of the heterogeneous nuclear ribonucleoprotein (hnRNP) complexes which provide the substrate for the processing events that pre-mRNAs undergo before becoming functional, translatable mRNAs in the cytoplasm.
BCAM
Laminin alpha-5 receptor. May mediate intracellular signaling.
MAP1B
Protein that belongs to the microtubule-associated protein family. The proteins of this family are thought to be involved in microtubule assembly,

Supplementary Figure 5. Analysis of downregulated proteins upon HOOK1 downregulation using STRING resource. Network of downregulated proteins in **(A)** OVCAR-8 and **(B)** SKOV-3 cell line. Network nodes represent proteins and edges represent protein-protein associations. Some genes are colored to show that they are associated to a specific GO term. **(C)** Network of the top 5 most significantly downregulated proteins in OVCAR-8 and SKOV-3 cell lines and brief description of their function within the cells.

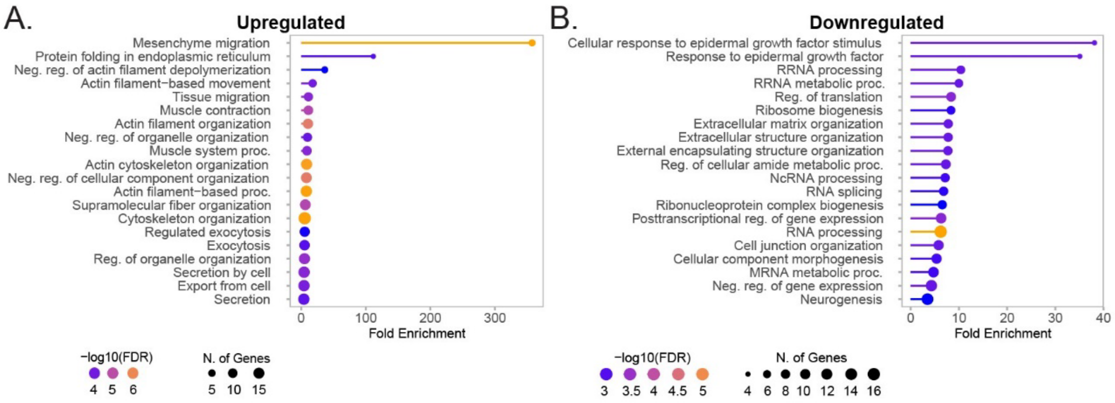

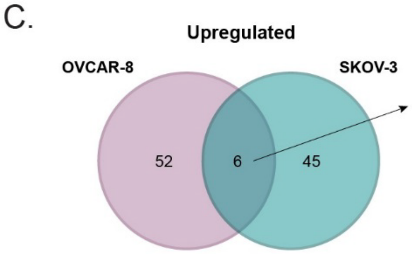

**HSPA5**	ER chaperone that plays a key role in protein folding and quality control
**HSP90B1**	Molecular chaperone that functions in the processing and transport of secreted proteins and in ER associated degradation (ERAD)
**PDIA4**	Disulfide isomerase that catalyze protein folding and thiol-disulfide interchange reactions in the ER
**GSN**	Calcium-regulated, actin-modulating protein that binds to the plus ends of actin monomers or filaments, preventing monomer exchange
**HYOU1**	Protein from the heat shock 70 family that has a pivotal role in cytoprotective mechanisms triggered by oxygen deprivation. May play a role as a molecular chaperone and participate in protein folding.
**LIMA1**	Actin-binding protein involved in actin cytoskeleton regulation and dynamics.

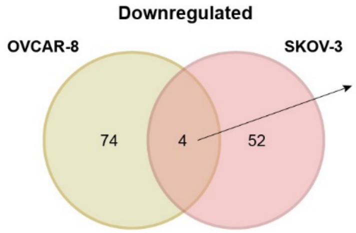

**GTF2I**	Binds to the initiator element (Inr) and E-box element in promoters and functions as a regulator of transcription.
**BAIAP2**	Adapter protein that links membrane-bound small G-proteins to cytoplasmic effector proteins.
**PRKDC**	Serine/threonine-protein kinase that acts as a molecular sensor for DNA damage
**BCAM**	Laminin alpha-5 receptor. May mediate intracellular signaling.

Supplementary Figure 6. Analysis of Gene Ontology and overlapping genes upon Hook1 downregulation. Gene Ontology terms of **(A)** upregulated (FC > 1) and **(B)** downregulated (FC < -1) proteins upon Hook1 downregulation in the SKOV-3 cell line. **(C)** Venn diagram of upregulated and **(D)** downregulated proteins in OVCAR-8 and SKOV-3 cell lines with downregulated HOOK1. A brief description of the overlapping genes is shown.

A.

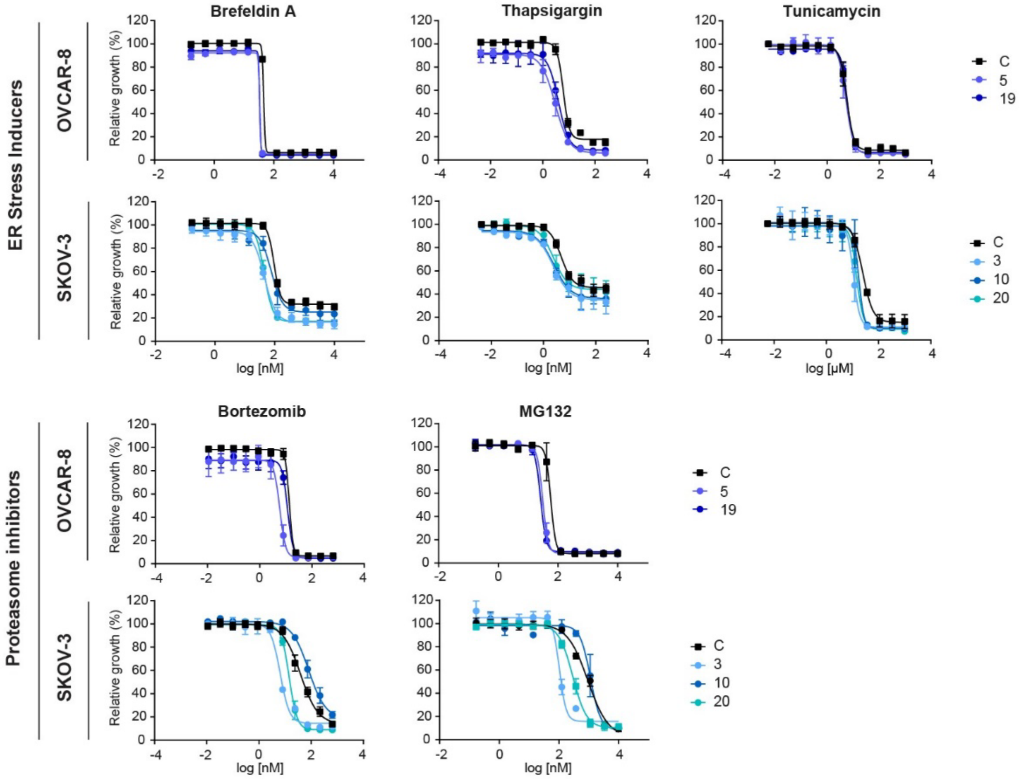

B.

ER Stress Inducers

		Brefeldin A (nM)	Thapsigargin (nM)	Tunicamycin (μM)
OV8	C	45,36 ± 0,41	6,27 ± 0,44	0,70 ± 0,08
	5	24,11 ± 5,02	3,26 ± 0,54	0,52 ± 0,07
	19	34,58 ± 1,14	4,04 ± 0,88	0,64 ± 0,05
SK3	C	107,97 ± 7,67	4,36 ± 0,51	2,77 ± 0,30
	3	47,66 ± 5,33	2,41 ± 0,46	1,23 ± 0,17
	10	75,00 ± 6,13	2,89 ± 0,35	1,41 ± 0,06
	20	46,61 ± 5,72	2,48 ± 0,24	1,34 ± 0,09

Proteasome inhibitors

		Bortezomib (nM)	MG-132 (nM)
OV8	C	15,39 ± 2,75	67,14 ± 18,17
	5	5,81 ± 0,47	30,88 ± 5,54
	19	14,65 ± 3,06	28,60 ± 5,29
SK3	C	36,51 ± 6,66	914,08 ± 75,46
	3	12,32 ± 3,35	104,72 ± 2,05
	10	70,73 ± 16,49	1100,19 ± 432,55
	20	16,87 ± 1,26	295,96 ± 38,99

Supplementary Figure 7. IC50 curves and mean values upon Hook1 downregulation. (A) IC50 curves of compounds that induce ER stress or inhibit proteasome function in Hook1-downregulated cells. The mean of 3 independent experiments ± SEM is represented. **(B)** Table with mean values of IC50 ± SEM of cells with downregulated HOOK1 treated with ER stress inducers or proteasome inhibitors.

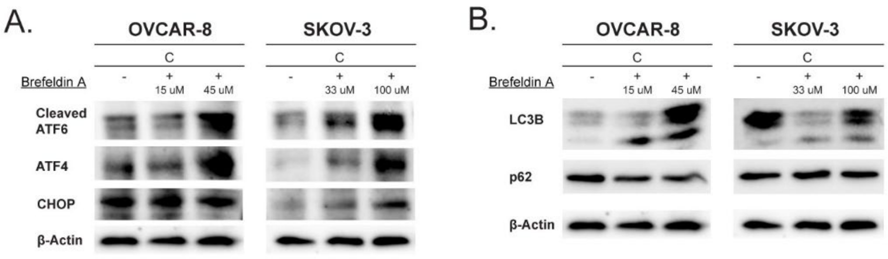

Supplementary Figure 8. Validation of brefeldin A treatment. (A) Protein levels of UPR-associated proteins in Hook1-downregulated cells treated with brefeldin A. **(B)** Protein levels of autophagy-associated proteins in Hook1-downregulated cells treated with brefeldin A.

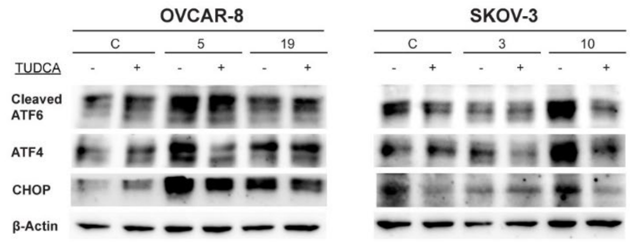

Supplementary Figure 9. Validation of TUDCA treatment. Protein levels of UPR-associated proteins in Hook1-downregulated cells treated with TUDCA.

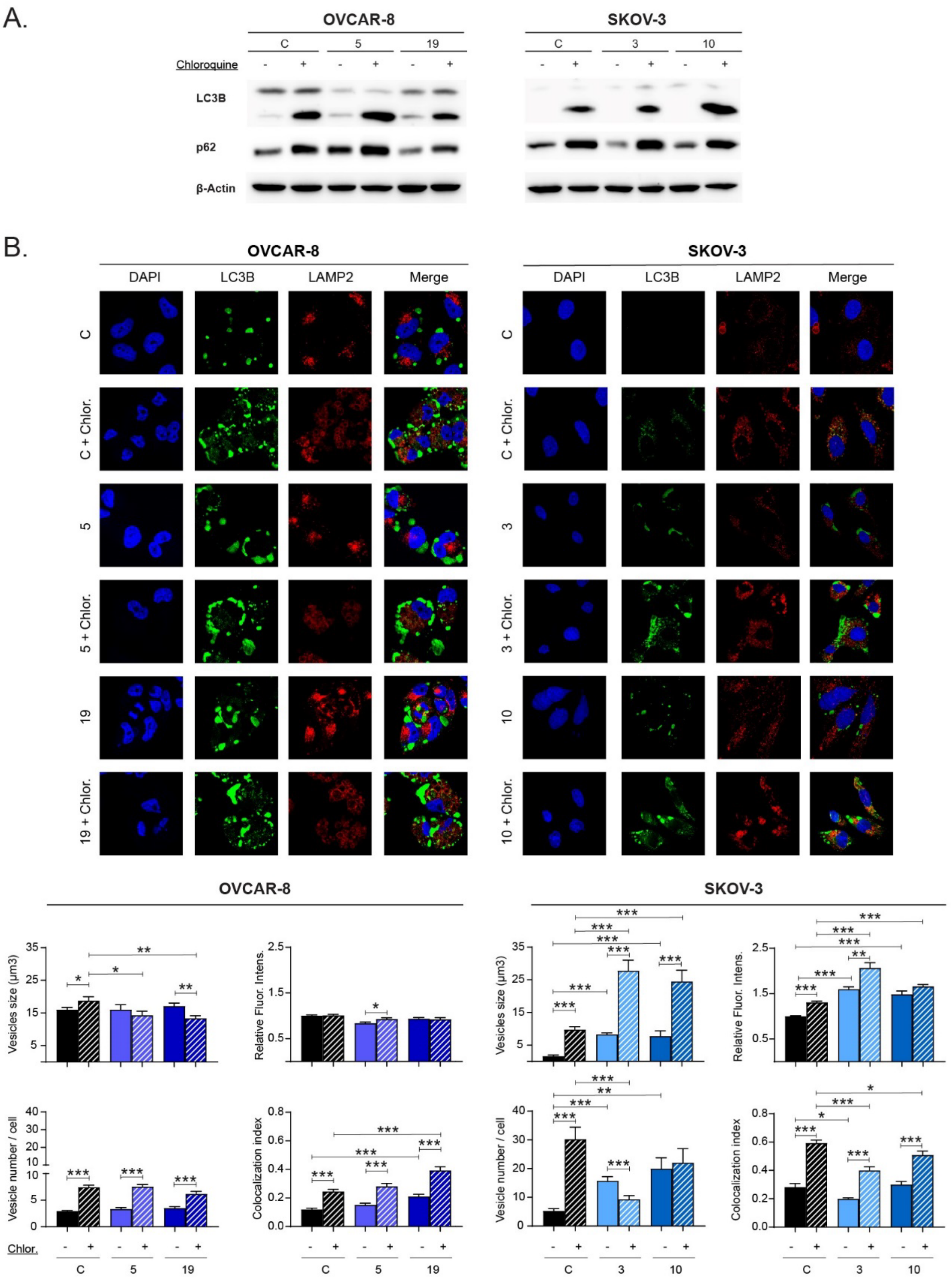

Supplementary Figure 10. Validation of chloroquine treatment. (A) Protein levels of autophagy-associated proteins in Hook1-downregulated cells treated with chloroquine. **(B)** Immunofluorescence staining of LC3B (green) and LAMP2 (red) proteins in Hook1-downregulated cells treated with chloroquine. DAPI (blue) was

used for nuclear staining, and the merge of the 3 markers is shown. The autophagosome size, number and relative fluorescence intensity, as well as the colocalization index of autophagosomes-lysosomes, were measured using ImageJ software. The statistical analysis was performed using Student’s t test (*$p<0.05$; **$p<0.01$; ***$p<0.001$).

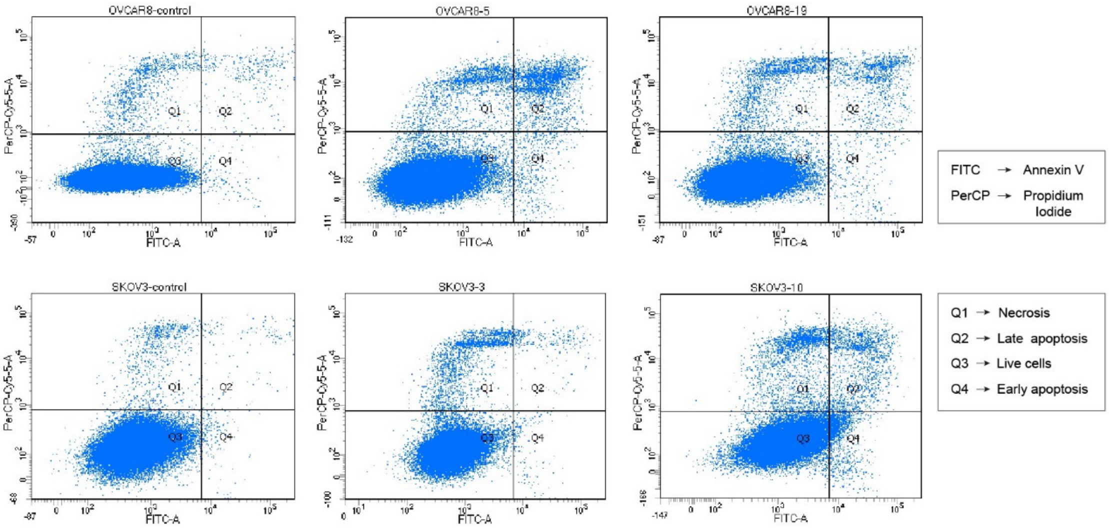

Supplementary Figure 11. Representative images of the cell death assay. Flow cytometry charts of ovarian cancer cells with downregulated HOOK1 stained with Annexin V and propidium iodide.

Treatment	Origin	Concentration OVCAR-8 line	Concentration SKOV-3 line
**Brefeldin A**	MedChemExpress	45 nM	100 nM
**Chloroquine**	MedChemExpress	25 µM	50 µM
**TUDCA (tauroursodeoxycholic acid)**	MedChemExpress	100 µM	100 µM

Supplementary Table 1. Concentration of the treatments employed in the ovarian cancer cell lines.

Figure 1D

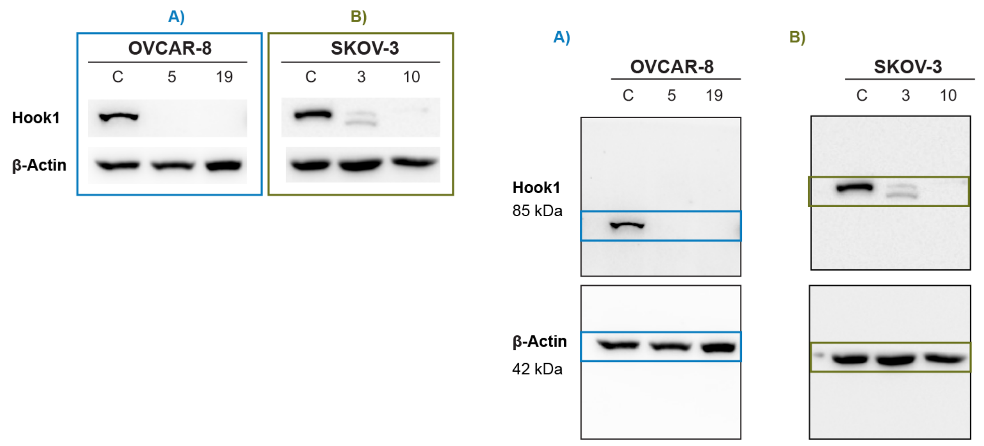

Figure 3D

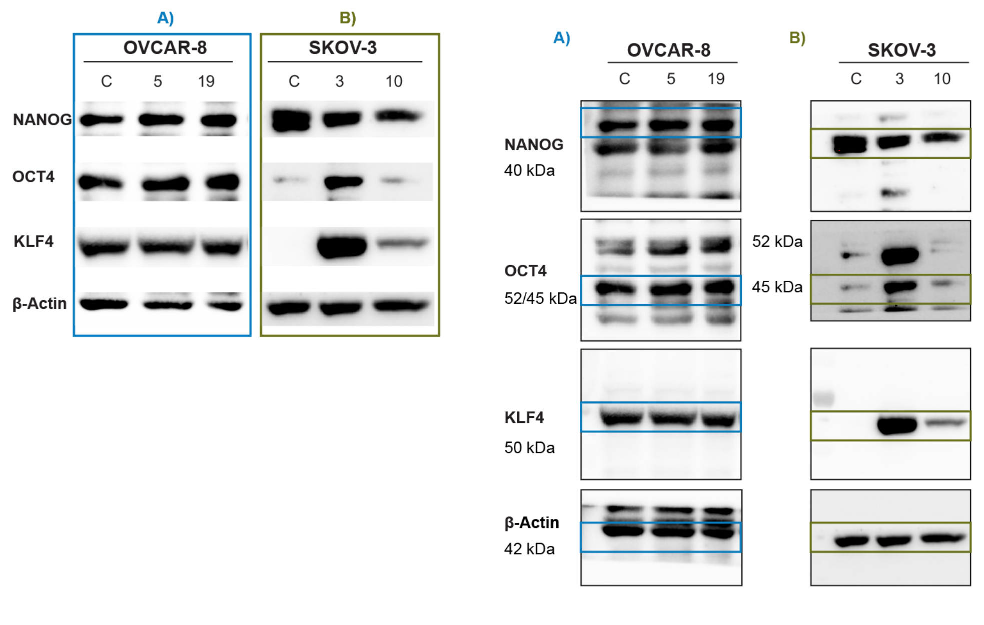

Figure 4E

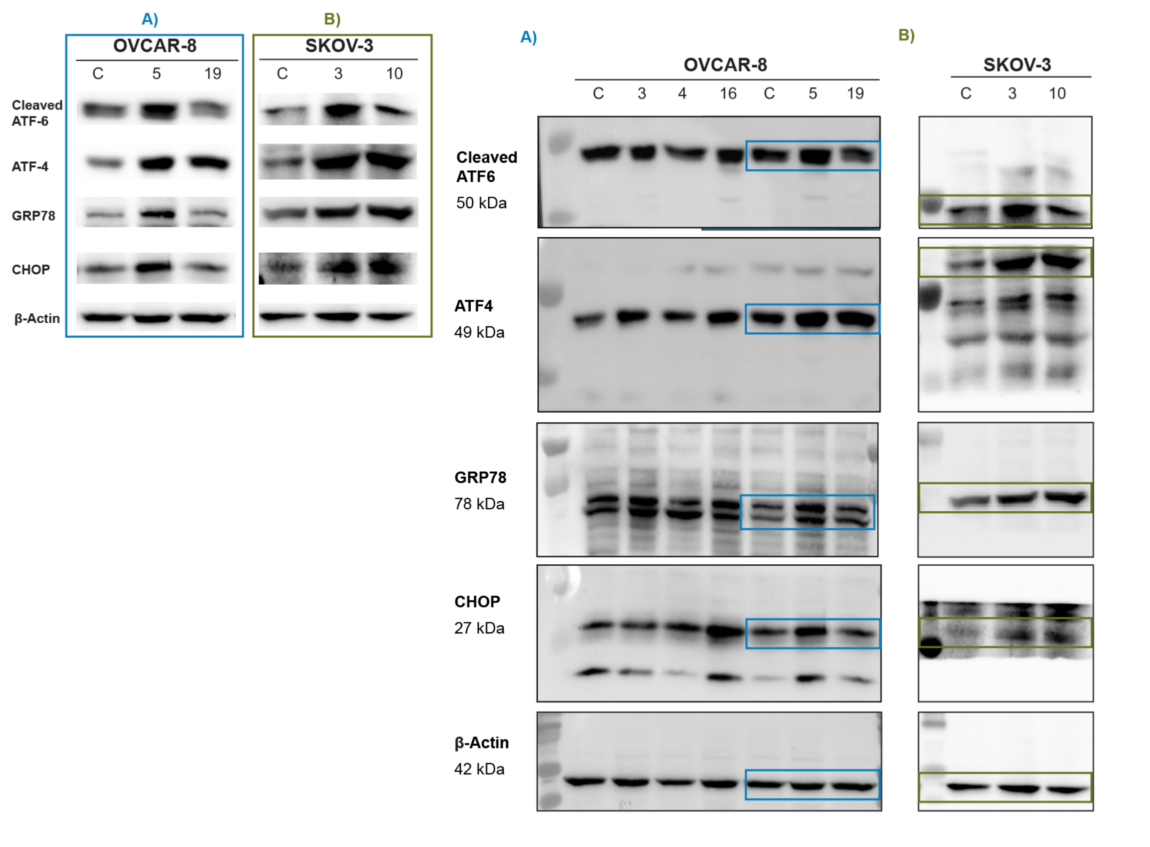

Figure 5A

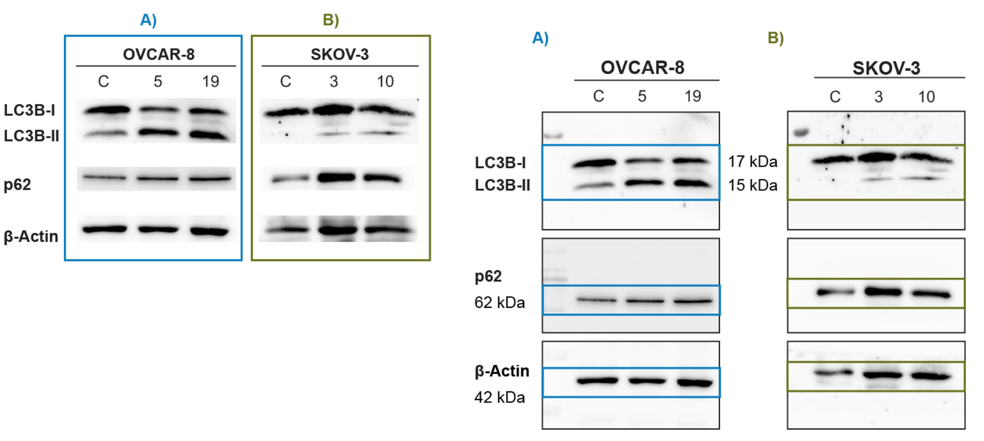

Figure 2C

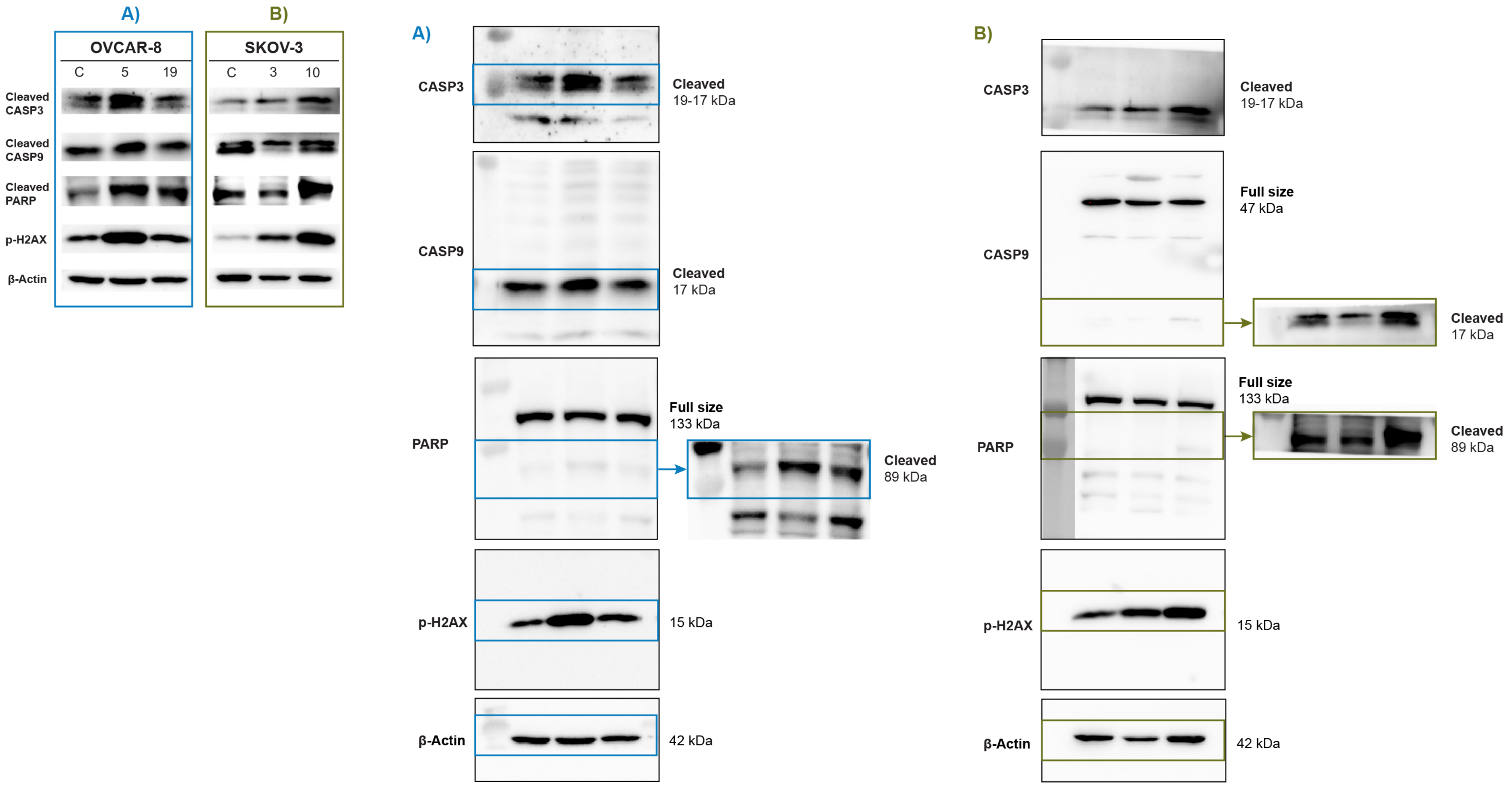

A)
B)
OVCAR-8
C
5
19
SKOV-3
C
3
10
Cleaved CASP3
Cleaved CASP9
Cleaved PARP
p-H2AX
β-Actin
A)
CASP3
Cleaved
19-17 kDa
CASP9
Cleaved
17 kDa
PARP
Full size
133 kDa
Cleaved
89 kDa
p-H2AX
15 kDa
β-Actin
42 kDa
B)
CASP3
Cleaved
19-17 kDa
CASP9
Full size
47 kDa
Cleaved
17 kDa
PARP
Full size
133 kDa
Cleaved
89 kDa
p-H2AX
15 kDa
β-Actin
42 kDa

Figure 6A

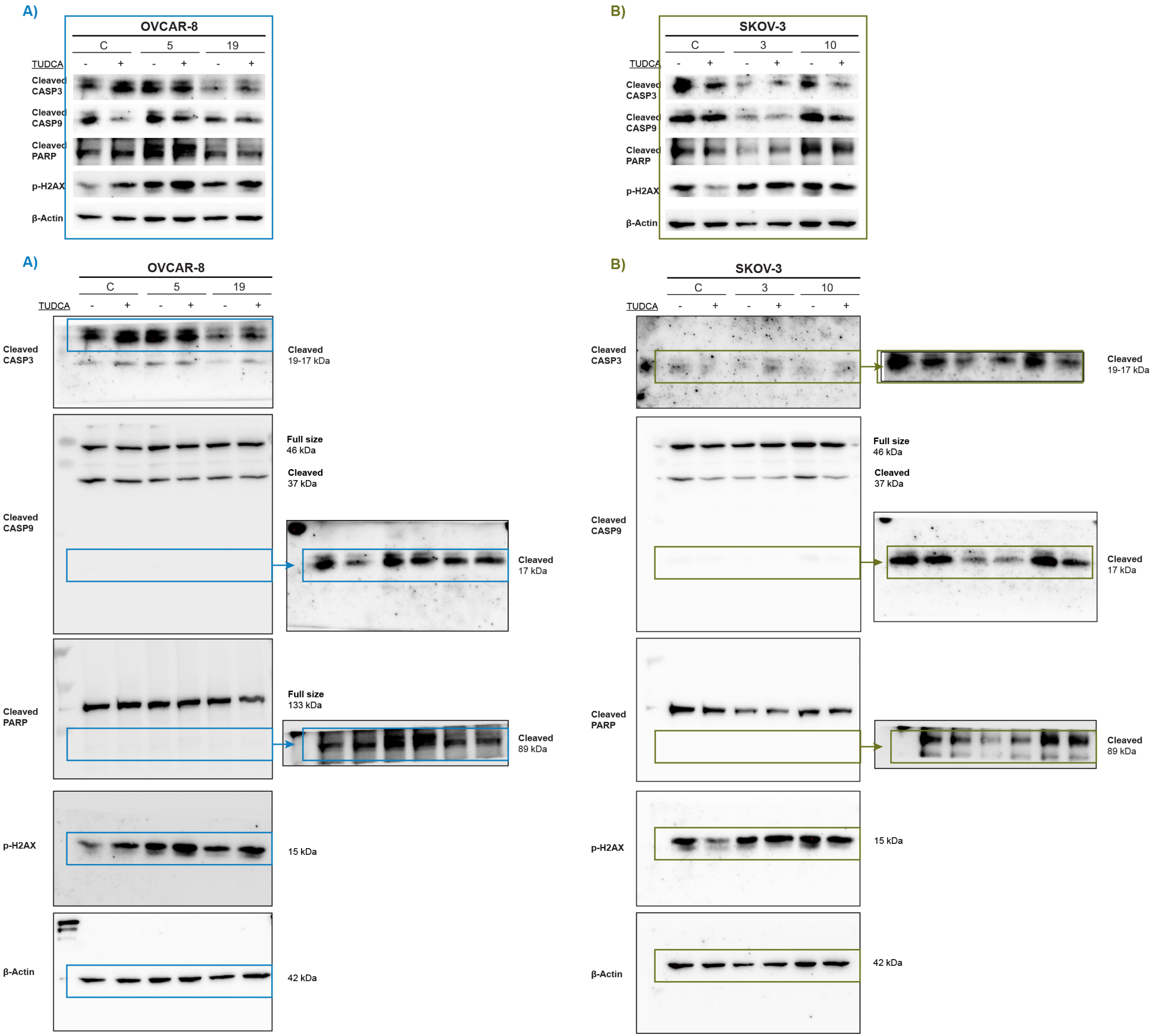

A)
OVCAR-8
C
5
19
TUDCA
Cleaved CASP3
Cleaved CASP9
Cleaved PARP
p-H2AX
β-Actin
B)
SKOV-3
C
3
10
TUDCA
Cleaved CASP3
Cleaved CASP9
Cleaved PARP
p-H2AX
β-Actin
Cleaved 19-17 kDa
Full size 46 kDa
Cleaved 37 kDa
Cleaved 17 kDa
Full size 133 kDa
Cleaved 89 kDa
15 kDa
42 kDa

Figure 6B

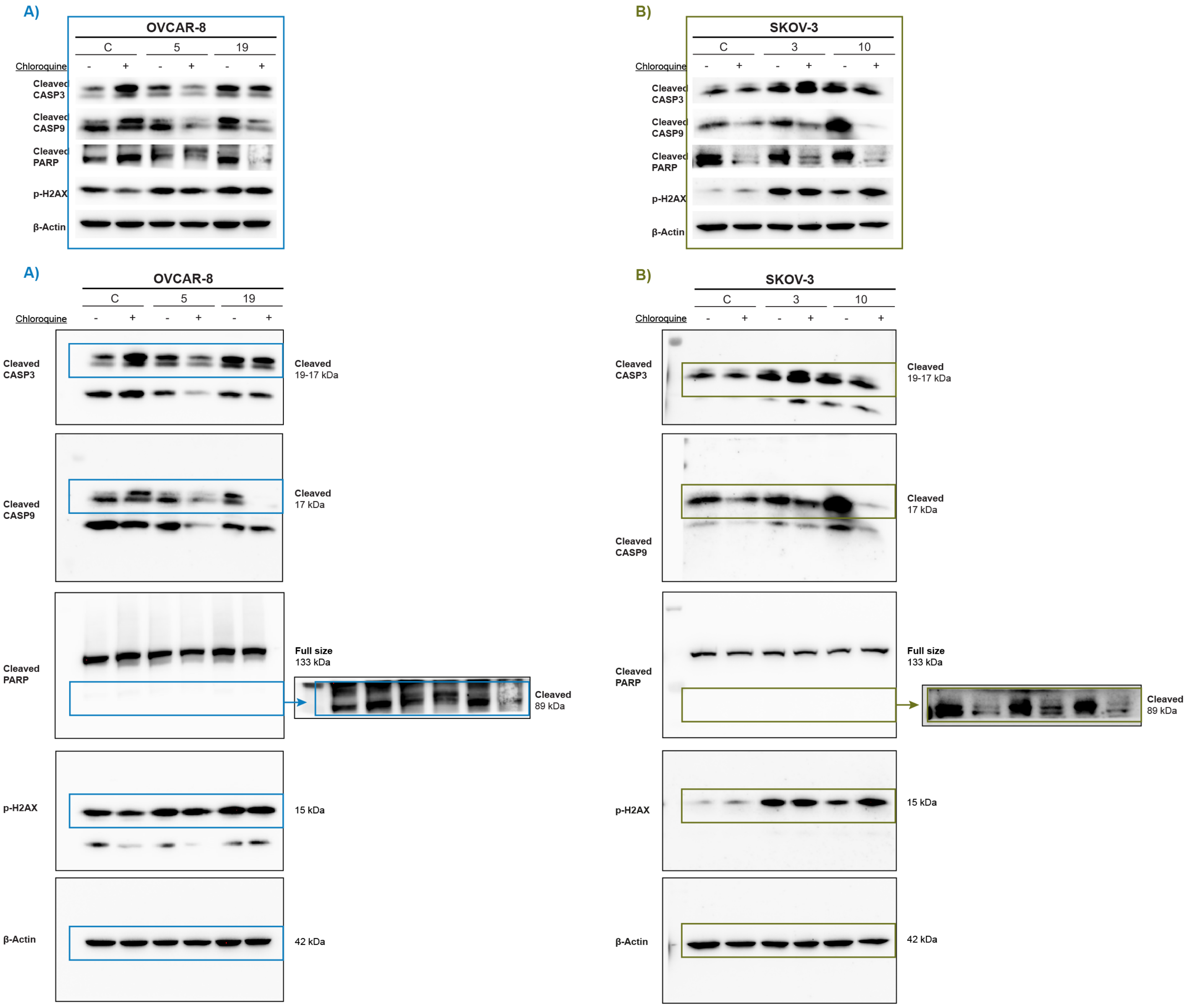
A)
OVCAR-8
C
5
19
Chloroquine
-
+
-
+
-
+
Cleaved CASP3
Cleaved CASP9
Cleaved PARP
p-H2AX
β-Actin
B)
SKOV-3
C
3
10
Chloroquine
-
+
-
+
-
+
Cleaved CASP3
Cleaved CASP9
Cleaved PARP
p-H2AX
β-Actin
A)
OVCAR-8
C
5
19
Chloroquine
- + - + - +
Cleaved CASP3
Cleaved 19-17 kDa
Cleaved CASP9
Cleaved 17 kDa
Cleaved PARP
Full size 133 kDa
Cleaved 89 kDa
p-H2AX
15 kDa
β-Actin
42 kDa
B)
SKOV-3
C
3
10
Chloroquine
- + - + - +
Cleaved CASP3
Cleaved 19-17 kDa
Cleaved CASP9
Cleaved 17 kDa
Cleaved PARP
Full size 133 kDa
Cleaved 89 kDa
p-H2AX
15 kDa
β-Actin
42 kDa
