# Supplementary material for: Protein homeostasis maintained by HOOK1 levels promotes the tumorigenic and stemness properties of ovarian cancer cells through reticulum stress and autophagy
Source: J Exp Clin Cancer Res. 2024 May 29;43:150. doi: 10.1186/s13046-024-03071-2 (PMC11134651; doi:10.1186/s13046-024-03071-2)
Supplement: Supplementary file 2 — Supplementary Material 2 [file 13046_2024_3071_MOESM2_ESM.pdf]

Reviwers reports:

Reviewer #1: This paper investigates the impact of HOOK1 gene knockout on ovarian cancer cells, examining cell growth, migration, tumor formation, and stem-like properties. Remarkably, the study unveils a reduction in stem-like characteristics post-HOOK1 knockout, attributed to heightened cell death within the cancer stem cell population induced by ER stress via the unfolded protein response.

While the findings offer valuable insights into the role of the Hook 1 gene across various biological processes and its potential as a therapeutic target, a few drawbacks warrant clarification and enhancement of rigor.

Thanks for your comments and valuable input to improve the manuscript.

1) Maintaining consistency in axis scales across histograms is crucial for accurately comparing data between different cell lines (e.g. panels in 1E; 1G; 2B; 2C; 3C; 3D, 4 E, 4F, ect.) and for facilitating a more reliable assessment of the differences or similarities between experimental conditions. Therefore, it is highly recommended that the authors address this issue to improve the overall quality and accuracy of their study.

Whenever is possible we have modified the axis making them uniform to maintain consistency in axis scales across histograms. As suggested, we have changed them in all figures except the axes from OCT4 and KLF4 from Figure 3D and axes from Figure 6, that have not been changed to match between the two cell lines because their ranges are too different, and the results wouldn't be easily readable. The values of cell death in Figure 6 have not been relativized to establish the same axis between the two lines, as this would result in losing information about the predominant type of cell death in each scenario (apoptotic vs. non-apoptotic).

Also, IC50 values in Figure 4F have been normalized relative to the control to ensure axis uniformity between the two cell lines. Additional information regarding Figure 4F can be found in new ***Supplementary Figure 7****.*

2) To enhance the clarity and interpretability of figure 3A, it is essential for the authors to specify what the optical images represent, such as different treatment groups, control conditions, or specific experimental manipulations.

To improve the interpretability of the experiment as the reviewer suggests, Figure 3A legend has been modified in order to give more insight into the different types of clones we observe and in what conditions the experiment was performed. New Fig legend reads as:

Figure 3. Hook1 downregulation causes a decrease in CSC-associated properties independent of the expression of genes traditionally linked to the CSC phenotype. *(A) Analysis of the type of clones originated from a clonability assay of ovarian cancer cell lines with downregulated Hook1. The percentage of holoclones, meroclones and paraclones was assessed. Representative images of each type of clone are shown.* ***(B)*** *Analysis of the number and size of tumorspheres of ovarian cancer cells with downregulated Hook1.* ***(C)*** *Relative mRNA levels and* ***(D)*** *relative protein levels of factors traditionally associated with stemness in ovarian cancer cell lines with downregulated Hook1. The mean of 3 independent experiments ± SEM is represented. Statistical analysis was*

*performed with Student's t test (*p < 0.05; **p < 0.01; ***p < 0.001). The absence of an asterisk means that the data are not statistically significant.*

Additionally, the text in the manuscript has been altered to give more information about where the concept of holoclones, meroclones and paraclones come from and what they represent.

*Due to the previously found physiological alterations, we wondered whether HOOK1 might also play a role in the generation or maintenance of CSCs in ovarian tumors. For that purpose, we analyzed the proportion of different types of clones formed after seeding the cells at low density (REFs newly incorporated in the new manuscript) and performed a tumorsphere assay. In the analysis of the clones type, we observed a significant decrease in the percentage of holoclones (clones enriched in CSCs), and an increase in the percentage of paraclones (clones enriched in differentiated non-stem cells) under all conditions with reduced levels of HOOK1 (**Figure 3A**).*

(page 7-8, lanes 141-147)

(REFs newly incorporated in the new manuscript)

- *Barrandon Y, Green H. Three clonal types of keratinocyte with different capacities for multiplication. Proc Natl Acad Sci U S A. 1987 Apr;84(8):2302–6.*
- *Li H, Chen X, Calhoun-Davis T, Claypool K, Tang DG. PC3 Human Prostate Carcinoma Cell Holoclones Contain Self-renewing Tumor-Initiating Cells. Cancer Res. 2008 Mar 15;68(6):1820–5.*
- *Beaver CM, Ahmed A, Masters JR. Clonogenicity: holoclones and meroclones contain stem cells. Nie D, editor. PLoS One. 2014 Feb 26;9(2):e89834.*

3) Is it biological relevant a 3%-8% cell death rate in figure 2B and 6.

*Decreasing HOOK1 leads to an increase in cellular death, with rates ranging from doubling to quadrupling those observed in the parental cell lines (**Figure 2B**). This increase is sustained and persistent over time. Consequently, we anticipate that this sustained rise in cell death will significantly diminish the viability of ovarian cancer cells, which is biologically pertinent for tumor development and progression. Moreover, considering the suggested cause of this heightened mortality (stemming from an increase in ER stress), it is reasonable to infer that under conditions of elevated stress, such as in vivo tumor growth, there would be an even more pronounced increase in cell death due to decreased levels of HOOK1. Particularly if this effect is predominantly in CSCs, tumor growth would be impeded. This could elucidate why there is a lack of in vivo growth of cells with downregulated HOOK1 when injected into mice.*

*This conclusion is further supported by the heightened cell death induced by cell stressors. It is noteworthy that the increase in cell death significantly escalates when cells with downregulated HOOK1 are treated with other drugs inducing cellular stress, such as those activating the UPR (**Figure 4F**) or blocking autophagic flux (**Figure 6B**). Particularly in the latter case, we observe an increase in cell death reaching 15% in the OVCAR-8 line and 30% in the SKOV-3 line. Therefore, we believe that reducing HOOK1 could hold significant promise in the treatment of ovarian cancer patients, especially when combined with compounds that increase proteotoxic stress.*

4) In Figure 7A, the percentage of cell death among Epcam positive SKOV-3 cells appears to exceed that of the total cell population. This anomaly prompts questions about the underlying biological mechanisms and experimental conditions.

Thanks for the comment.

*The percentage of total alive EpCAM+ cells (**Figure 7A**, upper graph) is low due to the preferential death of these cells (**Figure 7A**, middle graph). The events are measured in percentage, so we can compare the effects, and perhaps this can give the clear difference observed. We think that this data supports the underlying hypothesis.*

*Even though the experiment has been repeated several times, the low abundance of EpCAM+ cells in clone 10 may pose challenges in accurately quantifying the percentage of dead cells under this condition. To avoid any doubt, we replicated this experiment using a different CRISPR clone of HOOK1 to validate our findings. We have incorporated the data of said clone in new **Figure 7A**. In said clone we also observe a decrease in EpCAM+ cells, accompanied by an elevation in cell death rates. In this case, the increase in cell death among EpCAM+ cells was less pronounced compared to the total population. This might explain the less pronounced decrease in EpCAM+ cells observed, as compared to clone 10.*

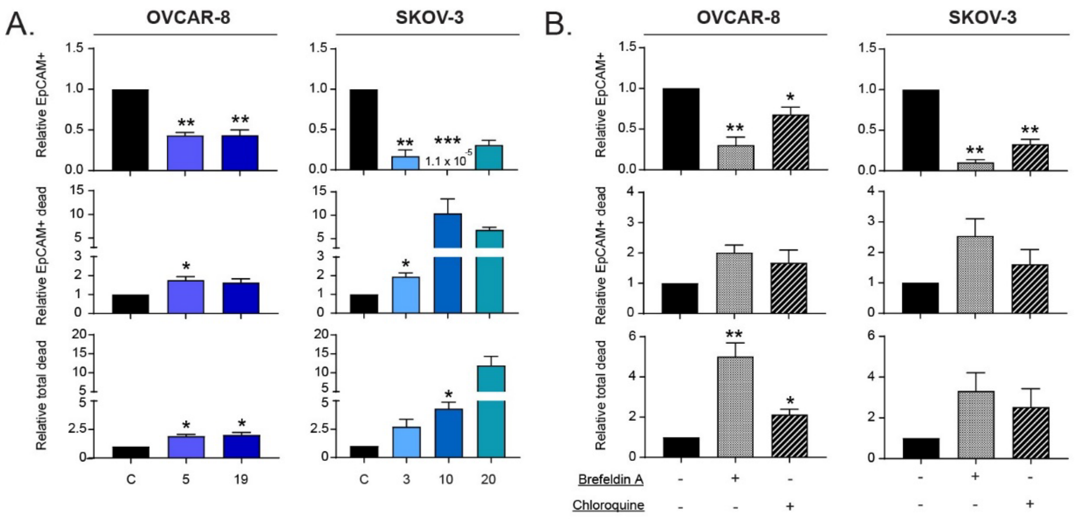

Figure 7. Cell death induced by Hook1 downregulation may selectively impact CSCs. (A) *Quantification by flow cytometry of the relative number of cells expressing the CSC marker EpCAM, EpCAM+ dead cells, and total dead cells in Hook1-downregulated cells.* ***(B)*** *Quantification by flow cytometry of the relative number of parental cells expressing the CSC marker EpCAM, EpCAM+ dead cells, and total dead cells after the treatment with an ER stress inducer (Brefeldin A) or an autophagic flux blocker (chloroquine). The mean of 3 independent experiments ± SEM is represented. Statistical analysis was performed with Student's t test (*p < 0.05; **p < 0.01; ***p < 0.001). The absence of an asterisk means that the data are not statistically significant.*

5) The absence of a legend in Figure 7 B severely impedes the comprehension of the results. Are reported the effects of inhibitors exclusively in wildtype cells? In summary, a thorough understanding of Figure 7's implications necessitates access to its legend and additional context

regarding the experimental setup, cell types, treatments. Without such information, a comprehensive interpretation remains elusive.

*Thanks for calling our attention on this. There must have been some mistake on our part when incorporating the legend for **Figure 7** in the text. We have included this legend and revised the to enhance clarity and have included it in its appropriate place within the text. The legend would be as follows:*

Figure 7. Cell death induced by Hook1 downregulation may selectively impact CSCs. (A) *Quantification by flow cytometry of the relative number of cells expressing the CSC marker EpCAM, EpCAM+ dead cells, and total dead cells in Hook1-downregulated cells.* ***(B)*** *Quantification by flow cytometry of the relative number of parental cells expressing the CSC marker EpCAM, EpCAM+ dead cells, and total dead cells after the treatment with an ER stress inducer (Brefeldin A) or an autophagic flux blocker (chloroquine). The mean of 3 independent experiments ± SEM is represented. Statistical analysis was performed with Student's t test (*$p < 0.05$; **$p < 0.01$; ***$p < 0.001$). The absence of an asterisk means that the data are not statistically significant.*

Addressing the questions of the reviewer, the effect of the drugs has been measured in wildtype cells to see if we could recapitulate the phenotype obtained by decreasing HOOK1 through increased ER stress or blockade of autophagy in parental cells.

The experimental setup is described in more detail in "Materials and methods". We have modified the section "Cell death assay in cells labeled with EpCAM by flow cytometry" to improve the description of the experimental design:

*The cells were maintained in culture with or without treatment for 48-72 hours (**Table S1**). Then, both suspended and adherent cells were collected. $1x10^6$ of those cells were resuspended in PBS with 2% FBS and 5 mM EDTA. Next, the cells were incubated with blocking agent (Miltenyi Biotec) for 10 minutes at 4 °C, and then, antibody labeling (Miltenyi Biotec) was performed for 15 minutes at room temperature and 15 minutes at 4 °C. Following this, two washes were performed using PBS with 2% FBS and 5 mM EDTA. After that, the previously described kit (Inmunostep) was used to label dead cells. Finally, the cells were examined using a FACSCanto II flow cytometer (BD Biosciences), and the results were analyzed using Diva software.*

(page 21, lanes 441-450)

6) In Figure 4B, it would be more appropriate to report the IC50 values on the curves rather than solely in the histogram, enhancing clarity and consistency.

*We included histograms in **Figure 4F** to enhance result interpretation, as we believed their readability to be superior to that of the IC50 curves. However, to improve data consistency and clarity, as suggested, we have now incorporated the IC50 curves into new **Supplementary Figure 7**. We have also adjusted the values in **Figure 4F** relative to the parental cells to maintain consistency of the axes between the two cell lines, as previously recommended by the reviewer. Therefore, and to improve clarity, we have included in **Supplementary Figure 7** a table with all the mean IC50 values +/- SEM for each condition:*

A.

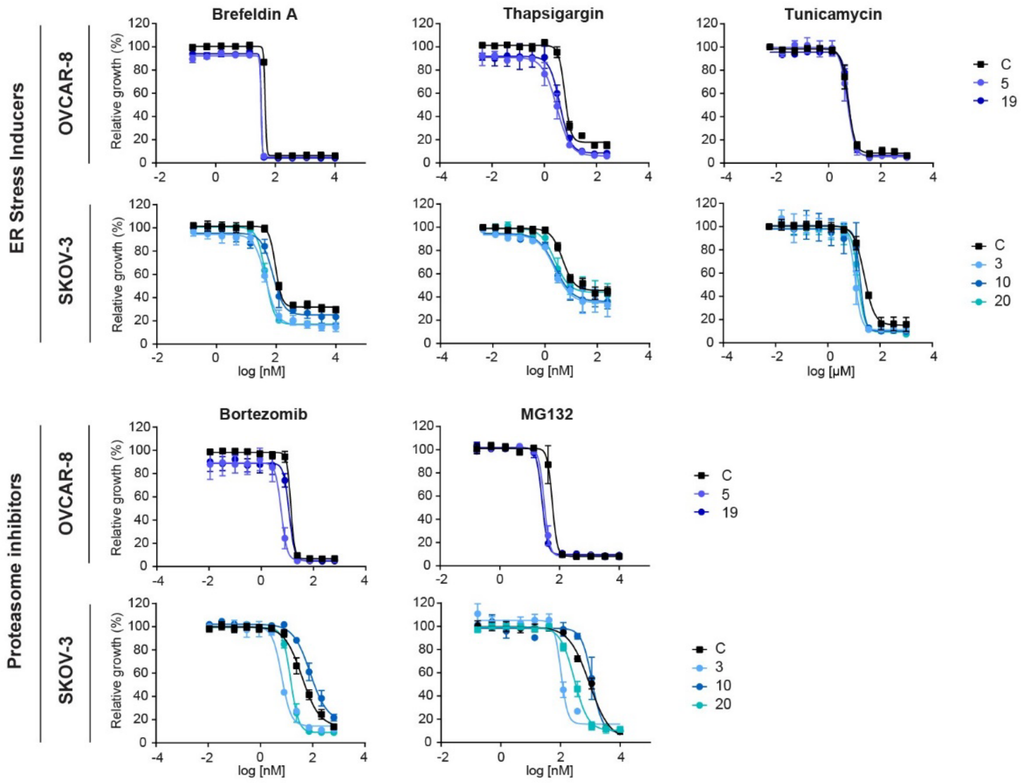

B.

ER Stress Inducers

		Brefeldin A (nM)	Thapsigargin (nM)	Tunicamycin (µM)
OV8	C	45,36 ± 0,41	6,27 ± 0,44	0,70 ± 0,08
	5	24,11 ± 5,02	3,26 ± 0,54	0,52 ± 0,07
	19	34,58 ± 1,14	4,04 ± 0,88	0,64 ± 0,05
SK3	C	107,97 ± 7,67	4,36 ± 0,51	2,77 ± 0,30
	3	47,66 ± 5,33	2,41 ± 0,46	1,23 ± 0,17
	10	75,00 ± 6,13	2,89 ± 0,35	1,41 ± 0,06
	20	46,61 ± 5,72	2,48 ± 0,24	1,34 ± 0,09

Proteasome inhibitors

		Bortezomib (nM)	MG-132 (nM)
OV8	C	15,39 ± 2,75	67,14 ± 18,17
	5	5,81 ± 0,47	30,88 ± 5,54
	19	14,65 ± 3,06	28,60 ± 5,29
SK3	C	36,51 ± 6,66	914,08 ± 75,46
	3	12,32 ± 3,35	104,72 ± 2,05
	10	70,73 ± 16,49	1100,19 ± 432,55
	20	16,87 ± 1,26	295,96 ± 38,99

Supplementary Figure 7. IC50 curves and mean values upon Hook1 downregulation. (A) *IC50 curves of compounds that induce ER stress or inhibit proteasome function in Hook1-downregulated cells. The mean of 3 independent experiments ± SEM is represented.* ***(B)*** *Table with mean values of IC50 ± SEM of cells with downregulated HOOK1 treated with ER stress inducers or proteasome inhibitors.*

7) Moreover, discrepancies arise in the Western blot analysis of **LC3B** expression in SKOV cells between Figure 5 and supplemental Figure 8. Such inconsistencies undermine the reliability of the findings and raise concerns regarding experimental reproducibility and data integrity. Addressing these discrepancies and ensuring accurate figure labeling and presentation are essential for maintaining the scientific rigor and credibility of the study.

We believe that the discrepancies noted by the reviewer may stem from different exposition of blots due to the high increase of LC3B-II band of cells treated with chloroquine.

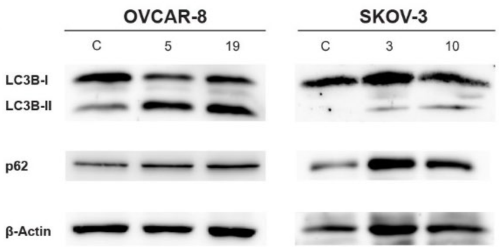

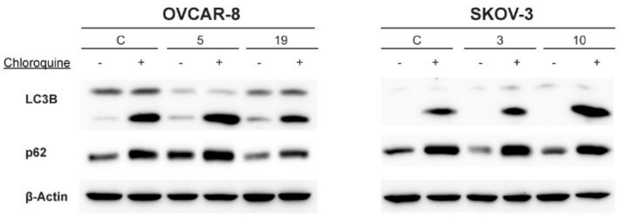

Figure 5A

New Supplementary Figure 10A

(previous Supplementary Figure 8A)

*In the SKOV-3 parental line under basal conditions (**Figure 5A**), the LC3B-II form is barely discernible in the WB, given the expectedly low levels of basal autophagy. In contrast, in the OVCAR-8 line, both forms of LC3B are distinctly observable in parental cells under basal conditions. In both lines, decreasing HOOK1 levels results in an increase in LC3B-II and the LC3B-II/LC3B-I ratio. However, in the SKOV-3 line, the LC3B-II band appears notably fainter, consistent with the baseline state of LC3B in the parental line.*

*When chloroquine is added to either of the cell lines (new **Supplementary Figure 10A**), as expected, there is a highly significant accumulation of LC3B-II, as its degradation is being inhibited. Consequently, we observe a substantial accumulation of LC3B-II across all conditions in both cell lines. Given that the baseline level of LC3B-II prior to chloroquine addition was higher in the OVCAR-8 line compared to SKOV-3, we are able to appreciate the LC3B-II band in untreated OVCAR-8 cells on the WB. However, in the SKOV-3 line, the significant LC3B-II accumulation upon chloroquine addition impedes observation of this band in untreated cells, as it would saturate the WB image exposure. Additionally, LC3B-I appears with considerably less intensity than in **Figure 5A**, again due to the significant accumulation of LC3B-II, that is masking the other form of LC3B. The only way to clearly observe LC3B in untreated cells in **Supplementary Figure 10A** would be to overexpose the WB membrane, thus losing LC3B information from the treated cells. Therefore, since in **Supplementary Figure 10A** we wanted to verify if chloroquine effectively blocked autophagic flux, we decided to use that WB image.*

8) The confusion arising from the authors' mislabeling of figures exacerbates the challenge of interpreting the findings accurately. Specifically, they misidentify supplemental figures, which may lead to misunderstanding or misinterpretation among readers. Additionally, in Figure 6, the incomplete labeling of the cell line name obstructs clear identification.

We apologize for the confusing labelling of the figures and how it might have caused a misinterpretation of the content of the article. We have modified the labels of the Supplementary Figures and verified that all of the Figures are correctly cited in the text. We have also corrected the incomplete labeling of Figure 6.

Specifically, the authors must clarify whether Hook1 significantly influences cell death and cancer stem cell (CSC) death. The observation that stem cell genes are upregulated post-knockout suggests a complex relationship between Hook and CSC regulation. To elucidate Hook's role, the authors should investigate the expression of stem cell genes in ovarian cancer cells with a

deletion of Hook1. This analysis would provide insights into how Hook1 deletion impacts CSC-related pathways and shed light on its therapeutic potential.

We believe that the decrease in HOOK1 significantly affects the death of ovarian cancer cells. In ***Supplementary Figure 2A****, we have incorporated the cell death rates of 5 additional CRISPR clones targeting HOOK1, further supporting our hypothesis. Notably, 4 of these newly introduced clones experience an increase in cell death. This increase is particularly notable in clones 3 of the OVCAR-8 line and 20 of the SKOV-3 line, where it is observed a tenfold increase in dead cells when compared to the parental cells. Regarding the death of CSCs specifically, we have added a new CRISPR clone to* ***Figure 7A****, providing more evidence that the decline in HOOK1 levels can specifically impact CSCs viability.*

(page 13, lanes 262-274).

*Regarding stem cell genes, to date, we have not found evidence that HOOK1 directly regulates the expression any of the genes related to stemness that we have examined (****Figure 3C and 3D****). We believe that the increase in KLF4 and OCT4 that we observe in some cases might be derived from the increased ER stress (****REF****). However, we cannot discard that HOOK1 may be involved in the regulation of stem genes that have not been identified in this study. Nevertheless, beyond the regulation of these genes, we think that the decrease in CSC viability resulting from HOOK1 reduction could be the reason for the decline in stem properties that we have observed. We believe that HOOK1 is necessary to control cellular stress, and such stress can induce cell death, especially in those cells that are more sensitive, such as CSCs.* ***We propose a mechanism in which the maintenance of protein homeostasis by HOOK1 levels promotes the survival of ovarian cancer cells by regulating ER stress and autophagy.***

REF

Zhang, D. et al. Regulation of the adaptation to ER stress by KLF4 facilitates melanoma cell metastasis via upregulating NUCB2 expression. J Exp Clin Cancer Res 37, 176, doi:10.1186/s13046-018-0842-z (2018).

Chiu, C-C et al. Grp78 as a therapeutic target for refractory head-neck cancer with CD24(-)CD44(+) stemness phenotype. Cancer gene therapy vol. 20,11 (2013): 606-15. doi:10.1038/cgt.2013.64

Yao, Xiaoli et al. "Endoplasmic reticulum stress confers 5-fluorouracil resistance in breast cancer cell via the GRP78/OCT4/lncRNA MIAT/AKT pathway." American journal of cancer research vol. 10,3 838-855. 1 Mar. 2020

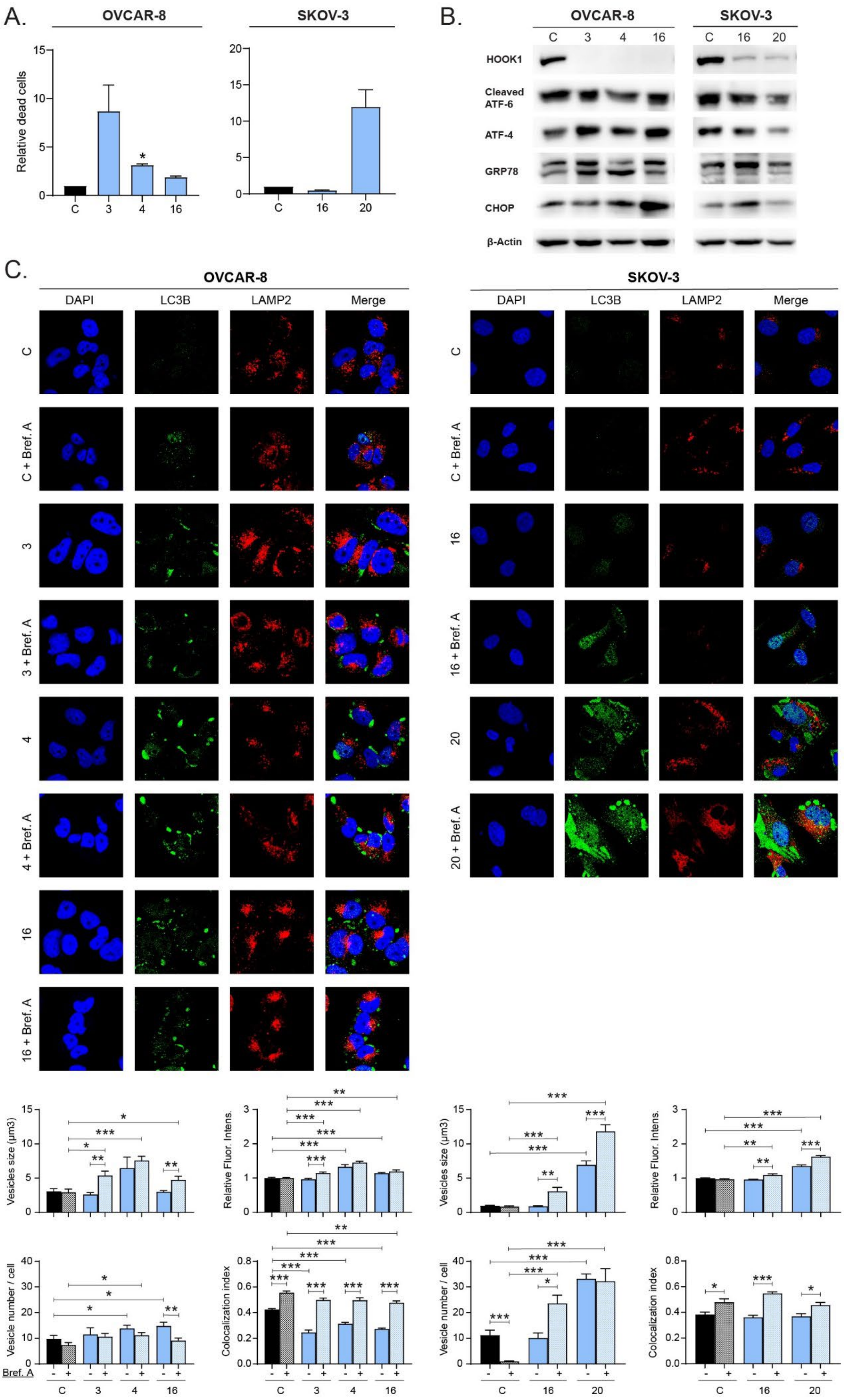
A.
OVCAR-8
SKOV-3
Relative dead cells
C
3
4
16
20
B.
OVCAR-8
SKOV-3
HOOK1
Cleaved ATF-6
ATF-4
GRP78
CHOP
β-Actin
C.
OVCAR-8
SKOV-3
DAPI
LC3B
LAMP2
Merge
C
C + Bref. A
3
3 + Bref. A
4
4 + Bref. A
16
16 + Bref. A
20
20 + Bref. A
Vesicles size (μm3)
Relative Fluor. Intens.
Vesicle number / cell
Colocalization index
Bref. A

Supplementary Figure 2. Analysis of cell death, ER stress and autophagy in different HOOK1 CRISPR clones in OVCAR-8 and SKOV-3 cell lines. (A) *Quantification by flow cytometry of the relative number of dead cells when Hook1 is downregulated.* ***(B)*** *Protein levels of UPR-associated proteins in Hook1-downregulated cells.* ***(C)*** *Immunofluorescence staining of LC3B (green) and LAMP2 (red) proteins in Hook1-downregulated cells treated with brefeldin A. DAPI (blue) was used as a nuclear stain, and the merge of the 3 markers is shown. The autophagosome size, number and relative fluorescence intensity, as well as the colocalization index of autophagosomes-lysosomes, were measured using ImageJ software.*

In summary, while the findings regarding Hook1 are promising, the paper's inconsistencies and lack of clarity necessitate revisions to ensure its suitability for publication. Clarifying Hook's effects on cell death and CSC regulation through additional experiments would strengthen the manuscript and contribute to our understanding of its therapeutic relevance in cancer.

Thanks for your comments. We hope that the new version clarifies these doubts.

Reviewer #2: The manuscript entitled "Protein homeostasis maintained by HOOK1 levels promotes the tumorigenic and stemness properties of ovarian cancer cells through reticulum stress and autophagy", by Suarez-Martinez et al., investigates the role of the protein HOOK1 in ovarian cancer.

Since this tumor entity is notoriously difficult to treat, this work is of great clinical interest. HOOK1 has been described as linker between intracellular vesicles and molecular motors that run along microtubules. The authors found HOOK1 to be overexpressed in ovarian cancer, and its downregulation impaired tumor cell proliferation in vitro and in vivo (s.c. injection in immune-deficient mice- but the actual number of experimental animals is indicated nowhere). These effects are attributed by the authors to a decreased tumor stemness upon downregulation of HOOK1. Mechanistically, this is explained by increased ER stress and an active UPR response in cells with low HOOK1 levels, and alterations in the autophagic flux. The authors present a nicely done study with an intriguing hypothesis, however, I do feel that some of their claims are not fully supported by the data. Whereas the tumorigenic properties of HOOK1 are clear and well documented by several orthogonal assays (except maybe for a model with forced expression that mimicks the higher HOOK1 levels seen in clinical samples), its effects on stemness are, at least in my view, not so convincing. Accordingly, the presumed influence on autophagic flux remains a bit vague, and no cell biological data are shown that would shed light on the involvement in kinesin/dynein-mediated vesicle transport, which could clear some of the central issues.

Thanks for your comments and valuable suggestions.

Specific points

Page 2: in the Abstract: why is it mentioned that Epcam was measured "only" by FACS?

It appears there was some English editing mistake during the Abstract submission process, as the content did not align with our intended message for the Methods section. We have rectified this mistake and present the revised version below:

Methods. *The downregulation of HOOK1 was performed in ovarian cancer cell lines using CRISPR/Cas9 technology, followed by growth in vitro and in vivo assays. Subsequently, migration (Boyden chamber), cell death (Western-Blot and flow cytometry) and stemness properties (clonal heterogeneity analysis, tumorspheres assay and flow cytometry) of the downregulated cell lines were analyzed. To gain insights into the specific mechanisms of action of HOOK1 in ovarian cancer, a proteomic analysis was performed, followed by Western-blot and cytotoxicity assays to confirm the results found within the mass spectrometry. Immunofluorescence staining, Western-blotting and flow cytometry were also employed to finish uncovering the role of HOOK1 in ovarian cancer.*

(page 2, lanes 28-35)

Page 5: the Crispr-KO clones show a "nearly complete" elimination of protein, but that means there is some remaining, residual expression of HOOK1 (maybe from escaping non-mutated cells that "contaminate" the cell cultures)? Has the mutated gene locus been sequenced?

*The term "nearly complete elimination of protein" has been used because in the SKOV-3 line, we still observe residual levels of the HOOK1 protein by WB. However, in the OVCAR-8 line, no residual protein is observed (**Figure 1D and Supplementary Figure 1**). All clones used in the study have been sequenced to determine if they had undergone modifications at the desired locus. These sequencing results are depicted in **Supplementary Figure 1B**. In the case of the SKOV-3 line, we consider that residual protein levels may persist because the cells are heterozygous for the mutated allele. Furthermore, due to the CRISPR generation method and the low expression levels of HOOK1 that we have consistently observed in this line, we do not believe that any escape of non-mutated cells has occurred to contaminate our culture.*

Recognizing that our previous phrasing in the text might lead to misunderstandings, we have revised that sentence in the manuscript to enhance clarity:

*The clones used for this study were selected among many by Western blot (WB) analysis (**Supplementary Figure 1A**) and sequenced (**Supplementary Figure 1B**) to confirm the mutation of the gene. In the OVCAR-8 cell line, a complete elimination of the protein was achieved, while in the SKOV-3 cell line, a very significant decrease in protein levels was observed. Additionally, in both cell lines, we observed very low residual mRNA levels compared to those of the parental line (**Figure 1D**).*

(page 6, lanes 113-118)

Page 5: as mentioned above, the number of mice injected is not indicated

We have included the number of mice injected per condition. It was 4. We have revised the "Xenograft in nude mice" section of the Methods to incorporate this information:

Xenograft in nude mice

Tumorigenicity was assayed by the subcutaneous injection of $4x10^6$ (OVCAR-8) or $5x10^6$ (SKOV-3) cells into the right flanks of four 4-week-old female athymic nude mice. Cells were suspended in Matrigel (Corning) prior to the injection. Animals were examined weekly. After 80-120 days, depending on the cell lines, mice were sacrificed, and tumors were extracted and conserved at -80 °C. Tumor volume (mm^3) was measured using calipers. All animal experiments were performed according to the experimental protocol approved by the IBIS and HUVR Institutional Animal Care and Use Committee (0309-N-15).

(page 21, lanes 451-458)

Page 6: p-H2AX is more a direct marker of DNA double strand breaks and DNA replication stress than "classical" apoptosis, was there a reason to presume that HOOK1-downregulation is inducing repair defects for DSBs?

Currently, no established link exists between the reduction in HOOK1 and defects in DSB repair mechanisms. We used p-H2AX as a marker for apoptosis because it plays a crucial role in DNA fragmentation during apoptosis and is phosphorylated at Ser139 by various kinases in response to apoptotic signals (Ref). Nevertheless, it is indeed noteworthy that pH2AX is an indicator of double-strand DNA breaks, suggesting potential future investigation into any closer association between decreased HOOK1 levels and replicative stress.

Ref:

- *Mukherjee B, Kessinger C, Kobayashi J, Chen BP, Chen DJ, Chatterjee A, et al. DNA-PK phosphorylates histone H2AX during apoptotic DNA fragmentation in mammalian cells. DNA Repair (Amst). 2006;5(5):575-90.*
- *Solier S, Sordet O, Kohn KW, Pommier Y. Death receptor-induced activation of the Chk2- and histone H2AX-associated DNA damage response pathways. Mol Cell Biol. 2009;29(1):68-82.*
- *Lu C, Zhu F, Cho YY, Tang F, Zykova T, Ma WY, et al. Cell apoptosis: requirement of H2AX in DNA ladder formation, but not for the activation of caspase-3. Mol Cell. 2006;23(1):121-32.*
- *Lu C, Shi Y, Wang Z, Song Z, Zhu M, Cai Q, et al. Serum starvation induces H2AX phosphorylation to regulate apoptosis via p38 MAPK pathway. FEBS Lett. 2008;582(18):2703-8.*

Page 6: please provide more details for the "clonability" assay - are the terms "paraclones" and "meroclones" etc. referring to the classical concept of Barrandon and Green (1987, I believe)?

The terms holoclones, meroclones, and paraclones in the clonal heterogeneity analysis indeed refer to the concepts initially described by Barrandon and Green in 1987. To facilitate understanding of this experiment, we have incorporated this reference into the manuscript text and updated the legend of Figure 3A.

*Due to the previously found physiological alterations, we wondered whether HOOK1 might also play a role in the generation or maintenance of CSCs in ovarian tumors. For that purpose, we analyzed the proportion of different types of clones formed after seeding the cells at low density (REFs newly incorporated in the new manuscript) and performed a tumorsphere assay. In the analysis of the clones type, we observed a significant decrease in the percentage of holoclones (clones enriched in CSCs), and an increase in the percentage of paraclones (clones enriched in differentiated non-stem cells) under all conditions with reduced levels of HOOK1 (**Figure 3A**).*

(page 7-8, lanes 141-147)

(REFs newly incorporated in the new manuscript)

- *Barrandon Y, Green H. Three clonal types of keratinocyte with different capacities for multiplication. Proc Natl Acad Sci U S A. 1987 Apr;84(8):2302–6.*
- *Li H, Chen X, Calhoun-Davis T, Claypool K, Tang DG. PC3 Human Prostate Carcinoma Cell Holoclones Contain Self-renewing Tumor-Initiating Cells. Cancer Res. 2008 Mar 15;68(6):1820–5.*
- *Beaver CM, Ahmed A, Masters JR. Clonogenicity: holoclones and meroclones contain stem cells. Nie D, editor. PLoS One. 2014 Feb 26;9(2):e89834.*

FIGURE 3

Figure 3. Hook1 downregulation causes a decrease in CSC-associated properties independent of the expression of genes traditionally linked to the CSC phenotype. ***(A)*** *Analysis of the type of clones originated from a clonability assay of ovarian cancer cells with downregulated Hook1. The percentage of holoclones, meroclones and paraclones was assessed. Representative images of each type of clone are shown.* ***(B)*** *Analysis of the number and size of tumorspheres of ovarian cancer cells with downregulated Hook1.* ***(C)*** *Relative mRNA levels and* ***(D)*** *relative protein levels of factors traditionally associated with stemness in ovarian cancer cell lines with downregulated Hook1. The mean of 3 independent experiments ± SEM is represented. Statistical analysis was performed with Student's t test (*$p < 0.05$*; **$p < 0.01$*; ***$p < 0.001$*). The absence of an asterisk means that the data are not statistically significant.*

Page 7: that is major issue: despite the fact that some properties of stemness (as assessed in vitro) seem to be regulated by HOOK1, the stem cell expression signature is not consistently altered (therefore, no effect on "classical" stem cell phenotype). This certainly is an interesting finding, but to claim that HOOK1 still regulates stemness, but "likely through a variety of different mechanisms" is a bit bold. Is it really "stemness" that is induced by HOOK1 (as defined by tumor induction in an animal model by injection limiting dilutions of cells)?

Our findings suggest that HOOK1 plays a regulatory role in the physiology of CSCs, although not directly targeting the core Yamanaka genes. It is plausible that the reduction in HOOK1 could influence the expression of other stemness-related factors not addressed in our analysis. Regardless of that, our observations indicate that diminished HOOK1 levels lead to increased ER stress and cell death, which we know specifically affects CSCs. We hypothesize that the reduction in viability of these cells could be the cause of the reduction in stem properties observed in our study, but without transcriptional alteration of stemnes genes. In the manuscript text, we have

attempted to clarify that HOOK1 may be involved in the "maintenance" of CSCs, referring to their survival. However, recognizing the potential for ambiguity, we have revised the text to ensure clarity regarding the relationship between HOOK1 and stemness:

*Next, we analyzed the mRNA levels of genes commonly involved in the generation of CSCs due to their involvement in the dedifferentiation process. Surprisingly, we did not find a significant decrease in these genes when HOOK1 levels were reduced. In fact, in some cases, there was an increase, such as in KLF4 (**Figure 3C**). To confirm these results, we performed WB analysis and we observed that there was an increase in the amount of OCT4 and KLF4 proteins in the SKOV-3 cell line. However, we did not observe significant changes in any of these proteins in the OVCAR-8 line (**Figure 3D**). Therefore, the involvement of HOOK1 in diminishing stem properties in ovarian cancer cells seems to be independent of the expression of these genes conventionally linked to stemness, suggesting the presence of an alternative mechanism to account for such alterations.*

(page 8, lanes 151-161)

Along that line, Epcam is used as single marker to identify cancer stem cells (Fig. 7), that is not really convincing, and other markers (e.g. CD44, CD24 etc.) should be included.

*To explore alternative markers for identifying CSCs, we initially analyzed the expression of various markers proposed in the literature for ovarian cancer (**REF1**) in the cell lines used in our study (**Supplementary Fig. 12A**). Notably, CD44 and CD24 exhibit remarkably elevated expression levels in both cell lines. In fact, previous experiments conducted in our laboratory showed that 100% of the cells in both lines are positive for CD44 by flow cytometry, but not all reconstitute the culture. Therefore, we decided to exclude this marker from our analysis. Regarding CD24, there are studies in ovarian cancer suggesting that CSCs could be CD24+, while others suggest they could be CD44+/CD24-, similarly to breast CSCs (**REF2, REF3**). Given the 100% CD44+ cells in both cell lines, we considered selecting CD24- cells to study them as potential CSCs in our model. As for CD105 and CD10, we observed that they are expressed in the OVCAR-8 line, while in the SKOV-3 line, they have very reduced (neglectable) expression levels. On the other hand, CD117 is expressed in the SKOV-3 line but not in the OVCAR-8 line. Finally, CD106 is not expressed in either of the cell lines (**Supplementary Fig. 12A**).*

*Based on these data, we selected potential CSC markers alternative to EpCAM for the studied cell lines. Flow cytometry analysis was conducted to determine the percentage of positive cells for these markers in cells with reduced levels of HOOK1. According to the gene expression data from The Human Protein Atlas, we did not find CD117+ cells in the OVCAR-8 line, nor CD10+ or CD105+ cells in the SKOV-3 line (**Supplementary Fig. 12A, 12B**). Additionally, we found approximately 40% of CD24- cells in the OVCAR-8 line and 10% in the SKOV-3 line. This high percentage of CD24- cells suggests that the ability to maintain the culture was not dependent on this marker. Therefore, we decided to exclude it from our study. Based on the results obtained, we ultimately decided to select CD105 in the OVCAR-8 line and CD117 in the SKOV-3 line to continue their study as CSC markers (**Supplementary Fig. 12B**).*

The flow cytometry analysis was conducted in triplicate for both cell lines using these markers. However, we found a significant variability in the percentage of cells positive for these markers across experiment replicates, especially in the CRISPR clones. Overall, we observed a positive correlation between cell passage number and the proportion of cells positive for these markers.

*This increase was accompanied by a reduction in the proportion of dead cells positive for these markers (**Supplementary Fig. 12C**). Therefore, these results would suggest that ovarian CSC populations expressing these surface markers could be dynamic, changing over time, and becoming resistant to death caused by the reduction of HOOK1.*

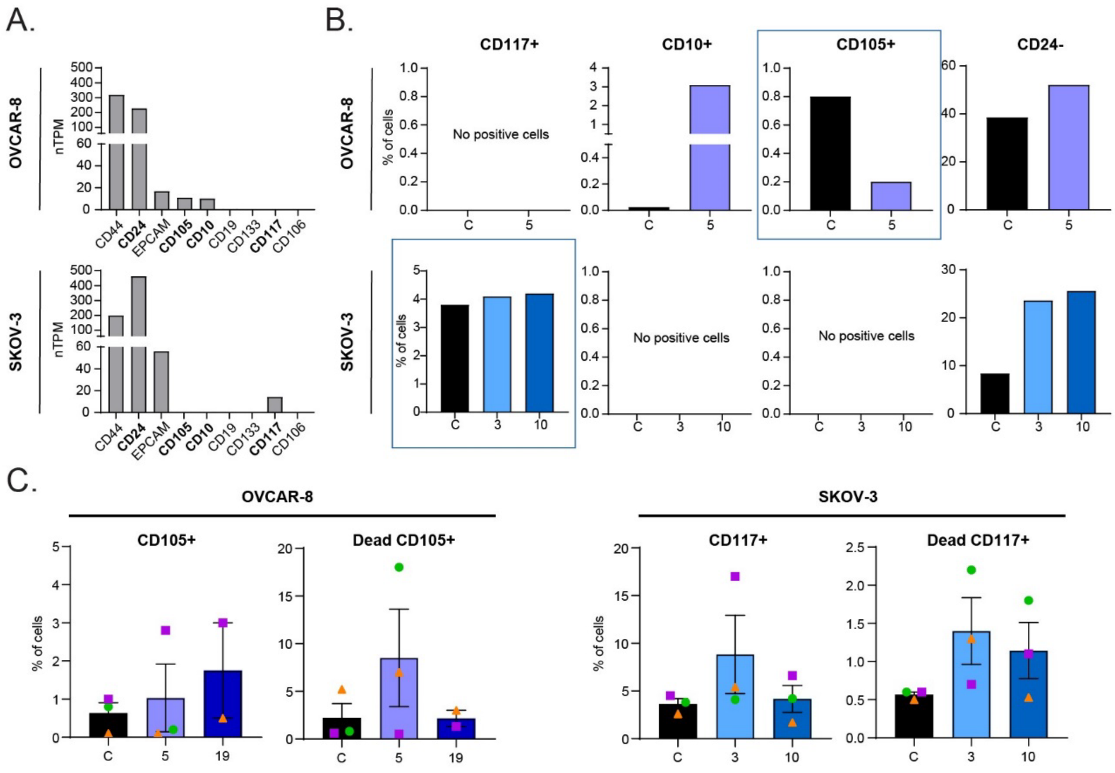

***Supplementary Figure 12. Study of CSC markers in the ovarian cancer cell lines OVCAR-8 and SKOV-3 with downregulated HOOK1. (A)** Normalized gene expression values of reported CSC markers in OVCAR-8 and SKOV-3 cell lines from The Human Protein Atlas. **(B)** Quantification by flow cytometry of the percentage of cells expressing a selection of the CSC markers suggested for ovarian cancer in OVCAR-8 and SKOV-3 cells with HOOK1 downregulation. **(C)** Quantification by flow cytometry of the percentage of CD105+ cells in OVCAR-8 cell line and CD117+ cells in SKOV-3 cell line with downregulated HOOK1.*

***REF1:** Królewska-Daszczyńska P, Wendlocha D, Smycz-Kubańska M, Stępień S, Mielczarek-Palacz A. Cancer stem cells markers in ovarian cancer: Clinical and therapeutic significance (Review). Oncol Lett. 2022 Nov 7;24(6):465. doi: 10.3892/ol.2022.13585. PMID: 36406181; PMCID: PMC9667454.*

***REF2**: Tarhriz V, Bandehpour M, Dastmalchi S, Ouladsahebmadarek E, Zarredar H, Eyvazi S. Overview of CD24 as a new molecular marker in ovarian cancer. J Cell Physiol. 2019 Mar;234(3):2134-2142. doi: 10.1002/jcp.27581. Epub 2018 Oct 14. PMID: 30317611.*

Gu Y, Zhou G, Tang X, Shen F, Ding J, Hua K. The biological roles of CD24 in ovarian cancer: old story, but new tales. Front Immunol. 2023 Jun 9;14:1183285. doi: 10.3389/fimmu.2023.1183285. PMID: 37359556; PMCID: PMC10288981.

REF3: *Meng E, Long B, Sullivan P, McClellan S, Finan MA, Reed E, Shevde L, Rocconi RP. CD44+/CD24- ovarian cancer cells demonstrate cancer stem cell properties and correlate to survival. Clin Exp Metastasis. 2012 Dec;29(8):939-48. doi: 10.1007/s10585-012-9482-4. Epub 2012 May 18. PMID: 22610780.*

Li, W., Ma, H., Zhang, J. et al. Unraveling the roles of CD44/CD24 and ALDH1 as cancer stem cell markers in tumorigenesis and metastasis. Sci Rep 7, 13856 (2017). https://doi.org/10.1038/s41598-017-14364-2

Sheridan C, Kishimoto H, Fuchs RK, Mehrotra S, Bhat-Nakshatri P, Turner CH, Goulet R Jr, Badve S, Nakshatri H. CD44+/CD24- breast cancer cells exhibit enhanced invasive properties: an early step necessary for metastasis. Breast Cancer Res. 2006;8(5):R59. doi: 10.1186/bcr1610. PMID: 17062128; PMCID: PMC1779499.

The next point where the interpretation of the data is not entirely consistent regards autophagy. The authors have tested two cell lines, and basically observed two opposing effects on autophagy (increase in OVCAR-8, and blockage in SKOV-3, Fig. 5). However, both cell lines show increased cell death upon downregulation of HOOK1. This is explained by ER stress (OK), and by "partial blockage of autophagy" (Discussion, p. 15), despite the fact that this only happens in one cell line, not the other.

*Thanks for the constructive criticism. We replicated these experiments again, this time incorporating a larger set of HOOK1 CRISPR clones (5 clones in total in the OVCAR-8 line and 4 clones in the SKOV-3 line). These repetitions, coupled with the introduction of novel clones, has led us to clarify the hypothesis previously presented in the article. We observed that cells with reduced levels of HOOK1 exhibit an accumulation of autophagic vesicles, alongside a decrease in autophagosome-lysosome colocalization in the OVCAR-8 line, as we described also in the SKOV-3 line. These consistent alterations were observed across all 5 distinct clones analyzed (**Figure 5B, Supplementary Figure 2C**). However, the introduction of Brefeldin A to cells with diminished HOOK1 levels appeared capable of normalizing autophagosome-lysosome colocalization to that observed in parental cells treated with this drug. In light of our previous and recent experiments, we propose that autophagic flux may be partially impeded in both cell lines upon HOOK1 reduction. While this partial obstruction would occur in both lines, it appears more pronounced in the SKOV-3 line, as even the addition of Brefeldin A fails to induce an increase in autophagosome-lysosome colocalization reaching parental cell colocalization levels (**Figure 2B**). Accordingly, we have modified the manuscript text to reflect our updated hypothesis:*

*To determine which of these two scenarios occurs in our model, we added brefeldin A to the cells to further increase ER stress ~~(Figure S3A)~~ and stimulate autophagy ~~(Figure S3B)~~ (**Supp. Fig. 8**) and analyzed autophagosome-lysosome fusion by immunofluorescence. ~~In the OVCAR-8 cell line, we observed that the number, size, and fluorescence intensity of autophagosomes were similar in cells with reduced HOOK1 and parental cells. Furthermore, upon brefeldin A treatment, the size and number of autophagosomes did not change significantly under either condition. However, autophagosome-lysosome colocalization was significantly increased by the treatment, especially in HOOK1 CRISPRs, where it significantly surpassed what we observed in the parental cells (Figure~~*

~~5B). These data suggest that cells with reduced HOOK1 levels in this cell line experience increased activation of autophagic flux to compensate for ER swelling.~~

*~~Nevertheless, in the SKOV-3 cell line,~~ We observed that cells with reduced HOOK1 levels, even without receiving any treatment, presented a higher number of autophagosomes than normal cells, and these vesicles were significantly larger than those observed in the parental cells. Furthermore, in the SKOV-3 cell line treated with the drug ~~after treatment~~, the number, size, and fluorescence intensity of these vesicles increased even further, surpassing the levels found in the control cells. However, we found that autophagosome-lysosome colocalization was reduced in cells with downregulated HOOK1, especially in cell line SKOV-3, where the addition of Brefeldin A was not able to replicate the colocalization levels observed in the control cells (**Figure 5B, Supp. Fig. 2C**). ~~upon treatment, autophagosome-lysosome colocalization significantly increased in the parental cells, while in the HOOK1 CRISPRs, the increase was not able to replicate the levels observed in the control cells (Figure 5B).~~ This finding suggests that there is some blockade in the autophagic flux ~~of the SKOV-3 cell line~~ when HOOK1 is downregulated. This blockade would prevent the fusion of autophagosomes with lysosomes, leading to an accumulation of both structures. ~~Hence, the reduction in HOOK1 affects autophagic flux; however, this phenomenon seems to be cell context dependent.~~*

(Page 11, lanes 220-232)

Minor points:

Fig. 1A: the labels are missing regarding the two groups in the boxplot

The labels of Figure 1A have been included.

Fig 1B: Typos "Estrctural"

The typo has been corrected. Fig 1B has been swapped with Fig 1C. Therefore, old Fig 1B is now new Fig 1C.

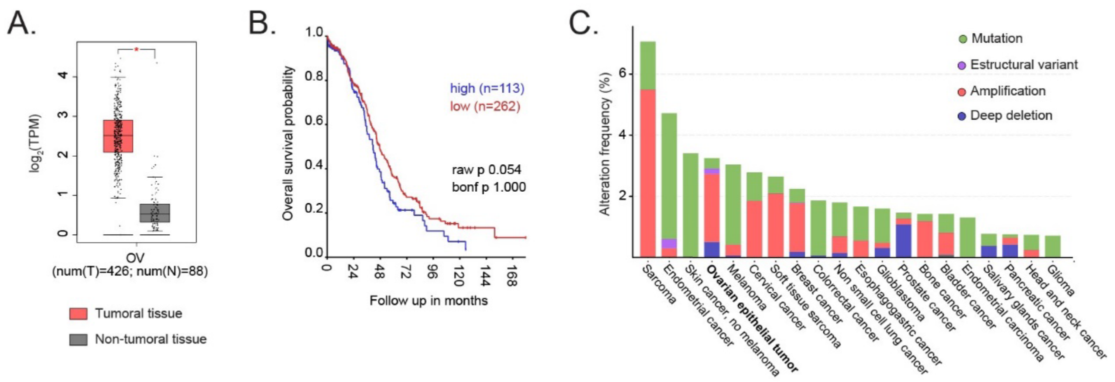

Throughout - the uncropped immuno blot should be provided, since some of the blots are not exactly "high quality"

We provide the uncropped immuno blots, that have been grouped in a PDF file called "Supplementary WB".

Fig. 8 the graphical abstract is a bit oversimplified and quite general, should either be revised or taken away

In our study, we have observed that the reduction of HOOK1 affects general cellular processes that can impact cell viability. Therefore, as suggested by the reviewer, the graphical abstract may appear overly simplistic since we have not identified specific signaling pathways affected. We have revised the graphical abstract to include more information about the article's content and to enhance understanding of the discussion. However, if the reviewers still find it too general, we have no objection to its removal from the publication.

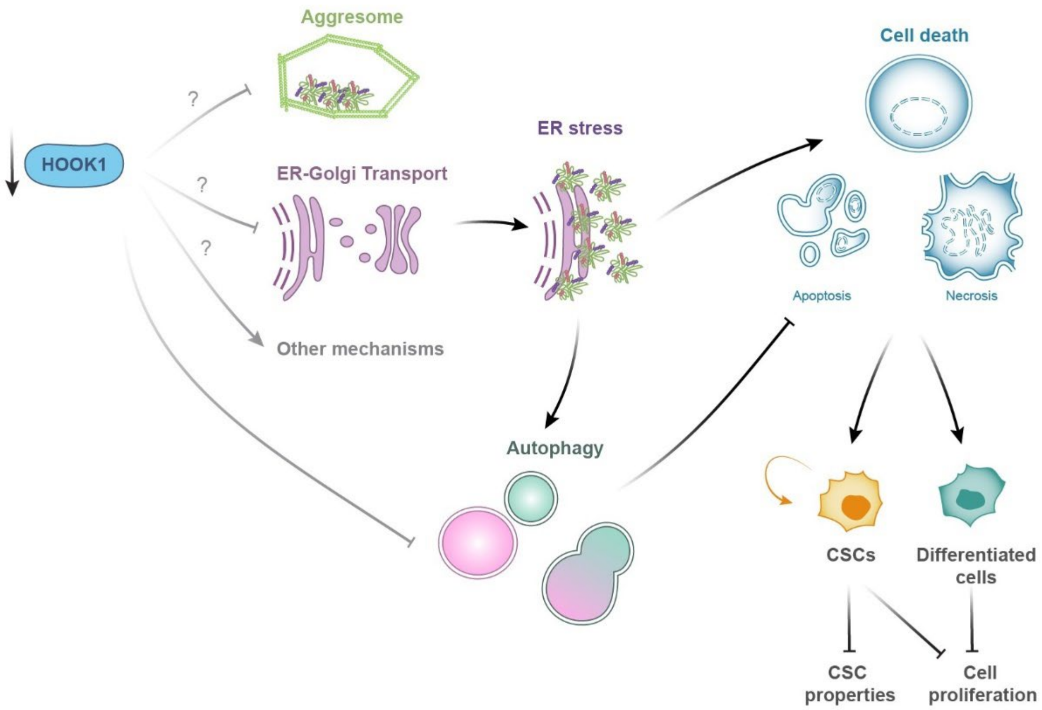

Figure 8. Interaction of HOOK1 with ER stress, autophagy, and cell death. *Decreased HOOK1 levels lead to the activation of ER stress, which activates signaling pathways that result in increased cell death and autophagy. Autophagy acts as a protective mechanism for the cell, reducing protein load and thus contributing to cell survival. However, the decrease in HOOK1 may also interfere with autophagic flux, causing a partial blockage of it and, therefore, reducing the effectiveness of this mechanism in preventing cell death. The increased cell death affects both differentiated cells and CSCs, causing a reduction of the cell proliferation and CSC properties of ovarian cancer cells.*

Reviewer #3: The authors analyze the impact of HOOK1 expression in the maintenance of stemness properties of two ovarian cancer cell lines. Following knockdown of HOOK1 by CRISPR/cas9 editing, they identify an impairment of cell growth in vitro and in vivo. Analyzing the whole proteome, they also aim to demonstrate by functional assays an increased ER stress

and autophagy which overall sustain an increased cell death. The study design and the experimental plan are appropriate but data description and interpretation should be improved. My major concerns are about the high variability observed at many different points (see also below) between the two cell lines and also among the different clones of the same cell line.

Although of potential interest, to correctly assess the biological relevance of the data shown, a higher number of clones should be considered at least in the key experiments showing activation of ER stress, autophagy and cell death.

*Key experiments regarding the reviewer's suggested processes have been conducted on several different CRISPR clones of HOOK1 in two different cell lines. Specifically, randomly selected 3 and 2 CRISPR clones were employed in OVCAR-8 and SKOV-3 cell lines, respectively. The decrease of HOOK1 in these clones was validated by WB (**Supplementary Fig. 2B**). To verify the increase in ER stress caused by the downregulation of HOOK1 in ovarian cancer, WBs of key proteins involved in UPR activation were performed. In the OVCAR-8 line, an increase in ATF-4 and CHOP was observed in the 2 studied clones. Similarly, in the SKOV-3 line, an increase in GRP78 and CHOP was found in in one of the clones studied (**Supplementary Fig. 2B**). These findings suggest that, although with some variability among the clones, the decrease of HOOK1 is capable of inducing a general increase in ER stress in ovarian cancer cells.*

*Furthermore, flow cytometry analysis was employed to examine changes in cell death experienced by cells with downregulated HOOK1. It was found that 4 out of the 5 clones with reduced levels of HOOK1 studied on this occasion experience an increase in cell death. This increase is particularly notable in clones 3 of the OVCAR-8 line and 20 of the SKOV-3 line, where it is observed a tenfold increase in dead cells when compared to the parental cells (**Supplementary Fig. 2A**).*

*Lastly, the autophagic flux status of these HOOK1-deficient clones was studied by immunofluorescence, both in the absence and presence of the ER stress inducer Brefeldin A. We observed that parental cells exhibited small amount of autophagic vesicles stained with LC3B, which slightly increased upon treatment with Brefeldin A. However, we can appreciate that, in both cell lines, cells with reduced levels of HOOK1 clearly show an accumulation of autophagosomes, and these vesicles are much larger than those in parental cells. Additionally, we can see that, while parental cells treated with Brefeldin A undergo a marked increase in autophagosome-lysosome colocalization, the same does not occur in cells with just reduced levels of HOOK1 (**Supplementary Fig. 2C**). These results indicate that the increase in autophagic vesicles observed when downregulating HOOK1 cannot be solely explained by the increase in ER stress. Indeed, in some of the clones, we even observe a decrease in autophagosome-lysosome colocalization (**Supplementary Fig. 2C**). This fact, combined with the presence of an increased number and size of autophagosomes, would again suggest that there may be some kind of blockage of autophagic flux when reducing HOOK1, leading to the accumulation of these vesicles.*

*Upon treatment with Brefeldin A, CRISPR clones with downregulated HOOK1 show an increase in the autophagosome-lysosome colocalization similar to that found in control cells (**Supplementary Fig. 2C**). Therefore, although autophagic flux may be partially blocked, it is not completely inactive. This observation aligns with the dependency of cells with reduced levels of*

*HOOK1 on autophagic flux, as evidenced by the pronounced increase in mortality of these cells observed upon chloroquine treatment (**Figure 6B**).*

In summary, we could conclude that the different clones with reduced levels of HOOK1 analyzed (5 in total in the OVCAR-8 line and 4 in total in the SKOV-3 line) behave similarly to each other and in both cell lines.

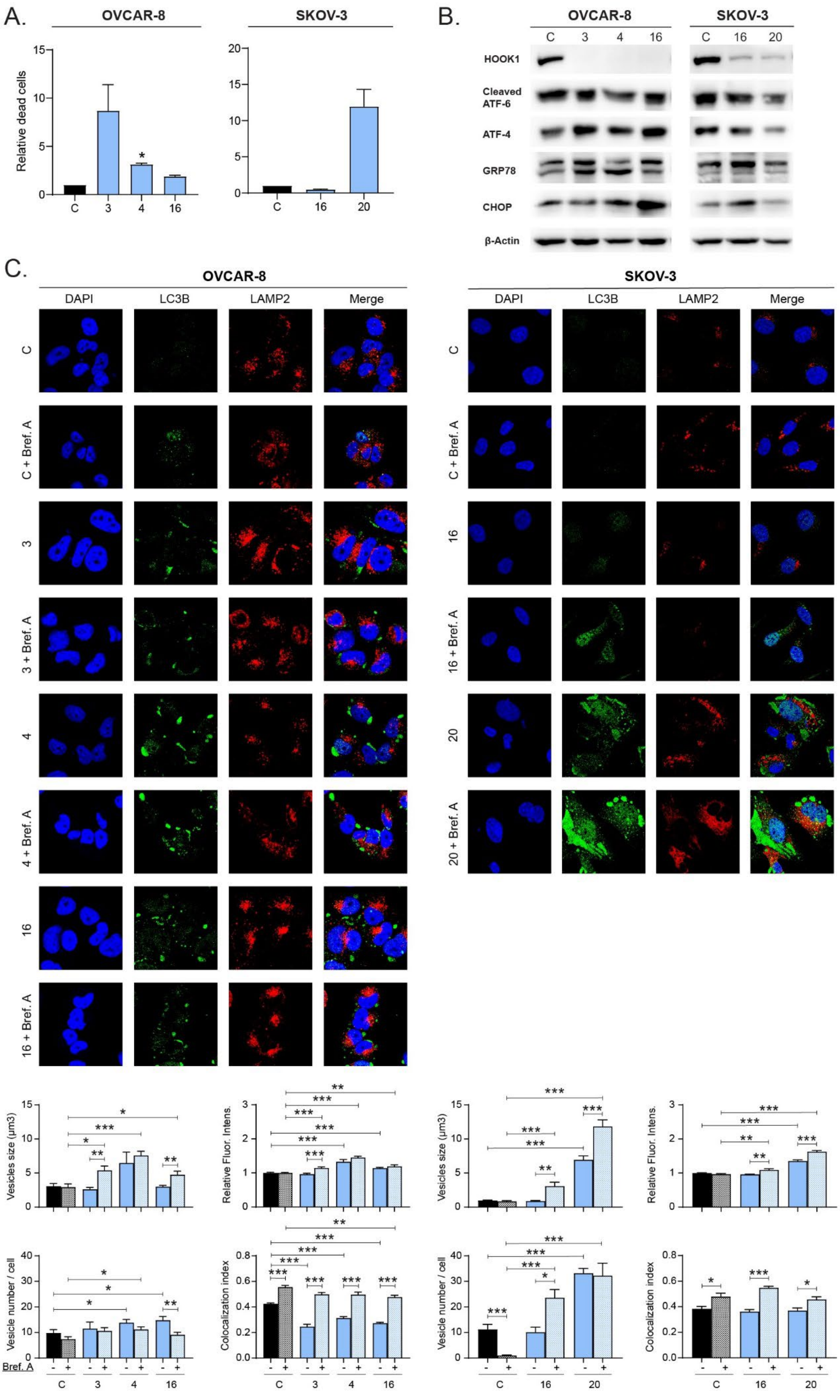
A.
OVCAR-8
SKOV-3
Relative dead cells
B.
HOOK1
Cleaved ATF-6
ATF-4
GRP78
CHOP
β-Actin
C.
DAPI
LC3B
LAMP2
Merge
C + Bref. A
3 + Bref. A
4 + Bref. A
16 + Bref. A
20 + Bref. A
Vesicles size (µm3)
Relative Fluor. Intens.
Vesicle number / cell
Colocalization index
Bref. A

Supplementary Figure 2. Analysis of cell death, ER stress and autophagy in different HOOK1 CRISPR clones in OVCAR-8 and SKOV-3 cell lines. (A) *Quantification by flow cytometry of the relative number of dead cells when Hook1 is downregulated.* ***(B)*** *Protein levels of UPR-associated proteins in Hook1-downregulated cells.* ***(C)*** *Immunofluorescence staining of LC3B (green) and LAMP2 (red) proteins in Hook1-downregulated cells treated with brefeldin A. DAPI (blue) was used as a nuclear stain, and the merge of the 3 markers is shown. The autophagosome size, number and relative fluorescence intensity, as well as the colocalization index of autophagosomes-lysosomes, were measured using ImageJ software.*

Below is a point by point listing of the other major issues

*In the Introduction lines 85-88 are not clear. Data available on HOOK1 expression/function in cancers should be better described with more insights and details.

A more detailed information about HOOK1 proposed role in cancer have been included in the Introduction. Some additional changes have been made in the Introduction in order to make it more concise and focused on the relation between HOOK1 and cancer.

The role of HOOK1 in cancer has not been explored in depth. However, a few studies have linked this protein to different types of tumors. For instance, a decrease in HOOK1 has been observed in hepatocellular carcinoma, where its downregulation has been associated with increased malignancy (REF1). Furthermore, HOOK1 has been proposed as a biomarker in papillary mucinous neoplasm following a proteomic screening (REF2). Nonetheless, the mechanism by which HOOK1 may be involved in cancer remains unclear. The most studied hypothesis in this regard is the association between HOOK1 and the phosphatase SHP2, implicated in tumorigenesis and metastasis in different types of tumors (REF3, REF4, REF5). However, the function of SHP2 as an oncogene or tumor suppressor gene is controversial and apparently depends on the tumor type (REF4). It has been described that HOOK1 is capable of binding to SHP2, and it has been suggested that it acts as an inhibitor of its phosphatase activity (REF4). In lung cancer cells, it was observed that while SHP2 positively regulates TEM, HOOK1 has the opposite effect (REF4). Nonetheless, other studies have linked HOOK1 to the progression of different types of tumors independently to its proposed interaction with SHP2 (REF1, REF6, REF7). Hence, the precise role of HOOK1 in cancer and its underlying mechanism remain unclear.

(page 5, lanes 79-92)

New REFs

RE1. Sun X, Zhang Q, Chen W, Hu Q, Lou Y, Fu QH, et al. Hook1 inhibits malignancy and epithelial-mesenchymal transition in hepatocellular carcinoma. Tumour Biol. 2017;39(7):1010428317711098.

RE2. Do M, Han D, Wang JI, Kim H, Kwon W, Han Y, et al. Quantitative proteomic analysis of pancreatic cyst fluid proteins associated with malignancy in intraductal papillary mucinous neoplasms. Clin Proteomics. 2018;15:17.

REF3. He L, Li Y, Huang X, Cheng H, Ke Y, Wang L. The prognostic significance of SHP2 and its binding protein Hook1 in non-small cell lung cancer. Onco Targets Ther. 2019;12:5897-906.

REF4. Li S, Wang L, Zhao Q, Liu Y, He L, Xu Q, et al. SHP2 positively regulates TGFβ1-induced epithelial-mesenchymal transition modulated by its novel interacting protein Hook1. J Biol Chem. 2014;289(49):34152-60.

REF5. Yang H, He L, Zhang Y, Li Y, Huang X, Lou Y, et al. The clinicopathological and prognostic implications of tyrosine phosphatase SHP2 and ankyrin Hook1 gene expression in non- small cell lung cancer patients treated with gemcitabine plus platinum as first-line chemotherapy. Ann Palliat Med. 2020;9(5):2943-52.

REF6. Cao, Kui et al. "Analysis of multiple programmed cell death-related prognostic genes and functional validations of necroptosis-associated genes in oesophageal squamous cell carcinoma." EBioMedicine vol. 99 (2024): 104920. doi:10.1016/j.ebiom.2023.104920

REF7. Yin, Lei et al. "HOOK1 Inhibits the Progression of Renal Cell Carcinoma via TGF-β and TNFSF13B/VEGF-A Axis." Advanced science (Weinheim, Baden-Wurttemberg, Germany) vol. 10,17 (2023): e2206955. doi:10.1002/advs.202206955

*Lines 99-106:

1. Description of HOOK1 alteration is not clear. Specifically, is not clear if the % of alteration frequency is referred to the total variations types or the total of case analyzed. Also, the relative percentages shown are not consistent with KM curves reported in figure 1C as the total of altered case is really few.

We have answered questions 1 and 2 together.

2. In Figure 1C key details need to be added: for each cohort it must be shown the number of cases and the number of events. However, since the two groups appear to be extremely unbalanced with the altered group having so few events, it would not appropriate to perform this type of analysis.

The 3.5% alteration frequency refers to all alterations found in the analyzed cases. Specifically, the percentage of amplifications in the total cases is 2.23%. In the Kaplan-Meier analysis 18 individuals compose the group with alterations and 552 individuals compose the group without alterations. Therefore, the percentage shown in the graph of alterations is consistent with the data presented in the Kaplan-Meier analysis. However, as highlighted by the reviewer, the size of the group with alterations is quite reduced, so probably it is necessary to increase the group with HOOK1 alterations to extract more robust conclusions. Therefore, we have analyzed by mRNA expression. See below.

3. Since the authors explored both GEPIA and cBIO portal I would consider the prognostic impact of HOOK1 mRNA expression rather than gene alteration, also considering their working hypothesis and the experiments that they performed in vitro (CRISPR knockdown)

Thank you for the suggestion. We agree with the reviewer that a Kaplan-Meier analysis separating ovarian cancer patients by levels of HOOK1 expression would better align with the

content of the article compared to grouping by alterations in HOOK1. Considering also the limited number of patients in the group with alterations in the previous analysis, we have replaced ***Figure 1C*** *with a new plot displaying overall patient survival stratified by HOOK1 expression levels. We have also swapped the positions of Figures 1B and 1C to maintain a logical order in the text. Consequently,* ***Figure 1C*** *now becomes the new* ***Figure 1B****. The manuscript text has been updated to incorporate the new information:*

*In previous studies, HOOK1 has been suggested to be linked to platinum resistance in ovarian tumors [24]. To undercover its significance in this type of cancer, firstly, we studied it in multiple databases. ~~Using the online database GEPIA,~~ We observed that HOOK1 expression was significantly higher in ovarian tumors than in normal tissue (**Figure 1A**) and that the overall survival tends to be worse in tumors where this gene is highly expressed (**Figure 1B**). In addition~~,~~ ~~in the cBioPortal database,~~ we found that there was a high percentage of ovarian tumors with amplifications in this gene (**Figure 1C**). Altogether, these data show that there is a high percentage of ovarian tumors with HOOK1 alterations and expression changes and that these ~~alterations~~ modifications may be associated with a worse prognosis in patients.*

(page 6, lanes 103-110)

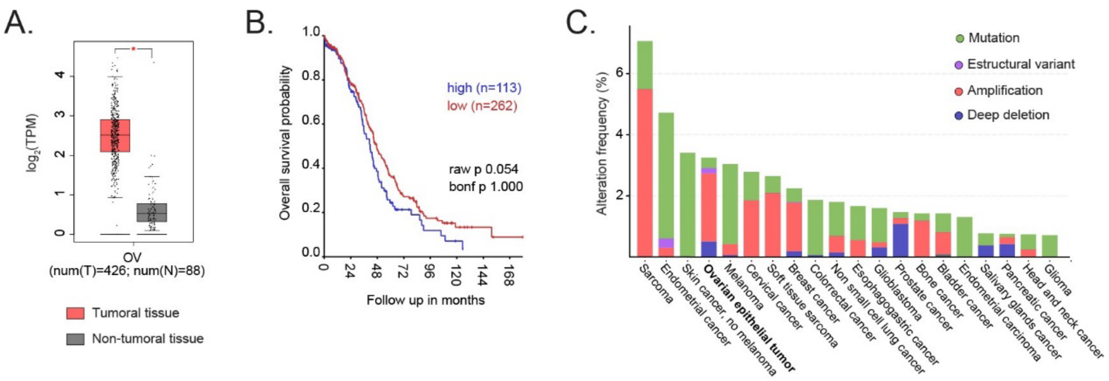

Figure 1. Hook1 is overexpressed in ovarian cancer patients, and its downregulation impairs the growth of ovarian cancer cells and abolishes the generation of tumors in vivo. (A) *Comparison of the expression of HOOK11 in ovarian tumors vs. normal tissue.* ***(B)*** *Overall survival of patients with high vs. low expression of HOOK1.* ***(C)*** *Analysis of Hook1 alterations in various human tumors.* ***(D)*** *Validation of HOOK1 downregulation in ovarian cancer cells by WB and RT–qPCR analyses.* ***(E)*** *Colony formation ability and* ***(F)*** *growth curve analysis of ovarian cancer cells with Hook1 downregulation. The mean of 3 independent experiments ± SEM is presented.* ***(G)*** *Evaluation of tumor growth of xenografts generated from Hook1-downregulated cells in comparison to parental cell lines. Graphs represent the tumor size (mean ± SEM). Representative images of the tumors are shown. Statistical analysis was performed with Student's t test (*p < 0.05; **p < 0.01; ***p < 0.001).*

4. Please add a reference for the GEPIA database.

A new section called "Public database analysis" has been incorporated to the Methods. In this section we include the references of all the databases used in this study.

Public Database Analysis

The following public databases were employed in this article: cBioPortal (http://www.cbioportal.org/) (REF1), GEPIA (http://gepia.cancer-pku.cn/) (REF2) and R2: Genomics Analysis and Visualization Platform (http://r2.amc.nl), which were employed to gather information on tumors from ovarian cancer patients.

(page 25, lanes 535-539)

*REF1. Cerami E, Gao J, Dogrusoz U, et al. The **cBio** cancer genomics portal: an open platform for exploring multidimensional cancer genomics data [published correction appears in Cancer Discov. 2012 Oct;2(10):960]. Cancer Discov. 2012;2(5):401-404. doi:10.1158/2159-8290.CD-12-0095*

*REF2. Tang Z, Li C, Kang B, Gao G, Li C, Zhang Z. **GEPIA**: a web server for cancer and normal gene expression profiling and interactive analyses. Nucleic Acids Res. 2017 Jul 3;45(W1):W98-W102. doi: 10.1093/nar/gkx247. PMID: 28407145; PMCID: PMC5570223.*

*Flow citometry assay to detect apoptosis and related data interpretation should be better described in both Materials and Methods and results section. Given the variability and in some cases the cells-specificity observed, at least in supplementary materials a representative FACS should be shown.

*We have improved the explanation of the flow cytometry conducted to detect apoptosis and the presence of CSC markers. The "Materials and Methods" section has been modified to better explain these experiments, and representative images of the flow cytometry charts have been included in **Supplementary Figure 11**. The text of the "Results" section has also been modified to enhance the interpretability of the experiments. Modifications in the text are underlined.*

MATERIALS AND METHODS

Cell death assay

*The cells were maintained in culture with or without treatment for 48-72 hours (Table S1). Then, both suspended cells and adherent cells were collected, and the 'Apoptosis Detection' kit (Immunostep) was used to measure cell death following the manufacturer's instructions. Briefly, cells were stained with Annexin V for 15 minutes at room temperature and in darkness. Subsequently, they were washed with Binding Buffer solution and centrifuged to remove the supernatant. Finally, they were incubated with propidium iodide for 5 minutes at room temperature. A FACSCanto II flow cytometer (BD Biosciences) was employed to detect the staining, and the results were analyzed using Diva software. Cells stained only with Annexin V were considered in early apoptosis, cells stained only with propidium iodide were considered in necrosis, and cells stained with both markers were considered in late apoptosis. All cells stained with propidium iodide were considered dead (**Supp. Fig. 11**).*

(page 20, lanes 429-439)

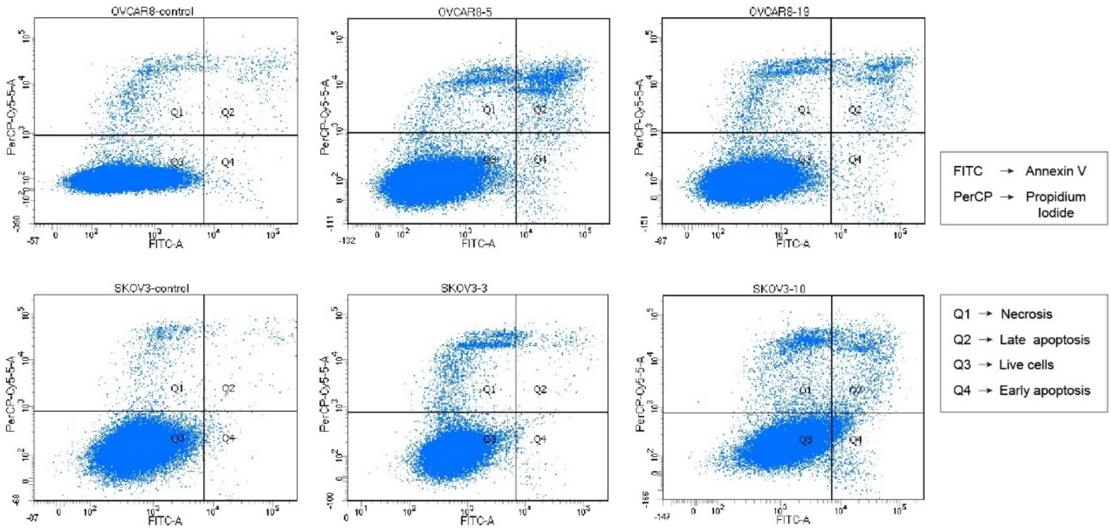

Supplementary Figure 11. Representative images of the cell death assay. *Flow cytometry charts of ovarian cancer cells with downregulated HOOK1 stained with Annexin V and propidium iodide.*

Cell death assay in cells labelled with EpCAM by flow cytometry

*The cells were maintained in culture with or without treatment for 48-72 hours (**Table S1**). Then, both suspended and adherent cells were collected. $1x10^6$ of those cells were resuspended in PBS with 2% FBS and 5 mM EDTA. Next, the cells were incubated with blocking agent (Miltenyi Biotec) for 10 minutes at 4 °C, and then, antibody labeling (Miltenyi Biotec) was performed for 15 minutes at room temperature and 15 minutes at 4 °C. Following this, two washes were performed using PBS with 2% FBS and 5 mM EDTA. After that, the previously described kit (Inmunostep) was used to label dead cells. Finally, the cells were examined using a FACSCanto II flow cytometer (BD Biosciences), and the results were analyzed using Diva software. The number of dead cells was measured in EpCAM+ cells and the whole population. All cells stained with propidium iodide were considered dead.*

(page 20-21, lanes 440-450)

RESULTS

1. HOOK1 affects the tumorigenic properties of ovarian cancer cells in vitro and impairs their ability to form tumors in vivo

*[…] For this purpose, we performed a flow cytometry assay using Annexin-V and a DNA intercalating agent. We observed that cells with reduced levels of HOOK1 showed an increase in the percentage of dead cells, both through apoptotic and nonapoptotic cell death (**Figure 2B, Supp. Fig. 2A, Supp. Fig. 11**). Then, we investigated the alteration of proteins related to apoptotic pathways, such as cleaved CASP3, CASP9, and PARP, and p-H2AX. Through WB analysis, we found an increase in the quantity of all the studied markers (Figure 2C). Thus, it appears that the reduction in HOOK1 increases the activation of the apoptotic pathway in ovarian cancer cells and leads to an increase in cell death, both through apoptosis-dependent and apoptosis-independent mechanisms. […]*

(page 7, lanes 128-136)

5. *Cell death induced by Hook1 downregulation is dependent on the ER stress response and autophagic flux.*

[…] As previously demonstrated, the reduction in HOOK1 levels is capable of activating apoptotic signaling pathways and causing an increase in cell death (Figure 4B, 4C). Therefore, we investigated whether the observed activation of the UPR in our model is responsible for this phenomenon. For that purpose, we used the drug tauroursodeoxycholic acid (TUDCA) to reduce ER stress levels (128). First, we confirmed that the drug effectively reduced ER stress in our model (Figure S4). Next, we studied cell death by flow cytometry and performed a WB of apoptosis-related markers. In the OVCAR-8 cell line, we observed that apoptotic markers did not change significantly in clone 19, while in clone 5, we observed an increase in cleaved PARP and p-H2AX. By flow cytometry, we found that TUDCA seemed to reduce cell death in clone 5; however, in clone 19, the changes in cell death were not as clear (Figure 6A). In the SKOV-3 cell line, there was a reduction in cleaved CASP3, CASP9, and PARP in CRISPR clone 10 but not in clone 3. Flow cytometry analysis showed that in both CRISPR clones, adding TUDCA to the medium reduced cell death. However, clone 10 exhibited a decrease in early-stage apoptosis, while clone 3 showed a reduction in necrosis, which may explain why we did not observe changes in apoptotic markers in this latter clone (Figure 6A). These results suggest that the inhibition of ER stress attenuates cell death in cells with reduced HOOK1 levels, although it does not completely rescue the phenotype.

*As previously demonstrated, reducing HOOK1 levels activates apoptotic signaling pathways, leading to increased cell death (**Figure 4B, 4C**). To investigate whether the activation of the UPR is responsible of this death, we used tauroursodeoxycholic acid (TUDCA) to reduce ER stress levels. Although TUDCA effectively reduced ER stress (**Supplementary Fig. 9**), its impact on cell death shows some clonal variability. In OVCAR-8 cells, apoptotic markers showed no significant changes, and TUDCA seemed to reduce cell death in clone 5 but had unclear effects in clone 19. In SKOV-3 cells, TUDCA reduced cleaved CASP3 and CASP9 and flow cytometry revealed reduced cell death in both clones, with clone 10 showing primarily a decrease in early-stage apoptosis and clone 3 displaying reduced necrosis (**Figure 6A**). Altogether, these findings suggest that inhibiting ER stress attenuates cell death in cells with reduced HOOK1 levels, albeit not completely rescuing the phenotype.*

*In contrast, activation of autophagic flux was reported to serve as a survival mechanism in cells with high ER stress. However, we found an impeded autophagic flux when we decreased HOOK1 levels (**Figure 5B, Supp. Fig. 2C**). Therefore, we considered it important in order to determine whether autophagy contributes protectively or inductively to cell death in our model. For this purpose, we used chloroquine as an inhibitor of autophagic flux [32]. As previously described, We first confirmed its effectiveness by WB and immunofluorescence analyses (Figure S5) **(Supp. Fig. 10).** Subsequently, we observed that when we added chloroquine to cells with reduced HOOK1 levels, some apoptotic markers, such as CASP3, CASP9 and PARP, were reduced in both cell lines (**Figure 6B**). However, by flow cytometry, we observed an increase in the percentage of dead cells after the treatment, which was significantly more pronounced in the cells with reduced HOOK1 levels. This increase was not due to the activation of apoptosis; instead, we found a very significant increase in the number of necrotic cells (Figure 6B). Therefore, these data suggest that cells with reduced HOOK1 levels are more sensitive to the blockage of autophagic flux, which*

*likely acts as a protective mechanism to restore cellular proteostasis. ~~Furthermore, when autophagy is blocked, the preferred route for cell death in HOOK1-depleted cells is apoptosis-independent.~~ This result, coupled with the increase of vesicle colocalization in CRIPSR clones following the addition of brefeldin A (**Figure 5B, Supp. Fig. 2C**), indicates that autophagic flux is not completely blocked in cells with reduced levels of HOOK1, retaining some degree of functionality.*

(page 12, lines 233-260)

6. CSCs might be particularly affected by the cell death induced by Hook1 downregulation

*Our data provide evidence that reducing HOOK1 leads to a decrease in cancer stem-like properties in ovarian cancer cells. However, this phenomenon was not dependent on genes traditionally associated with dedifferentiation. Therefore, we studied whether there could be a relationship between the reduction in stem-like capabilities and the occurrence of ER stress-associated cell death when HOOK1 was reduced. To investigate this, we analyzed the percentage of dead cells positive for EpCAM, a CSC marker. In both ovarian cancer cell lines, we observed that downregulating HOOK1 caused a decrease in the percentage of EpCAM-positive cells compared to that of parental cells. ~~This decrease occurred in all CRISPR clones but was particularly striking in clone 10 of the SKOV-3 cell line. In this clone, we found an approximately 10-fold increase in cell death compared to that of the parental line. In all other clones, the reduction in HOOK1 levels increased the death rate of EpCAM-positive cells by approximately 2-fold (**Figure 7A**).~~ The analysis of cell death specifically in EpCAM+ cells revealed an approximately 2-fold increase in cell death in most HOOK1 CRISPRs in comparison to parental cells. Strikingly, clone 10 of SKOV-3 cell line showed a 10-fold increase in cell death compared to that of the parental line. This result correlates with the very reduced percentage of EpCAM+ cells that we found in said clone (**Figure 7A**). These data support the hypothesis that the reduction in HOOK1 leads to a preferential decrease in CSCs in ovarian cancer due to an increase in their cell death, probably due to unsustainable ER stress. […]*

(page 13, lines 262-274)

*The authors did not observe variations in stem marker expression upon HOOK1 downregulation. These data should be better discussed. Would it be possible that HOOK1 acts downstream of the yamanaka factors? It would be interesting to evaluate if impairment of one on these factors may impact on HOOK1 expression.

The point raised by the reviewer is truly intriguing. In this study, our focus has been on elucidating the effects of HOOK1 on ovarian cancer cells rather than exploring its regulatory mechanisms. However, as the author suggests, it is possible that HOOK1 is downstream of one of the Yamanaka factors, which would be interesting to explore for future research. To date, no investigation has probed the interplay between Yamanaka factors and HOOK1. Due to the lack of time to conduct a more in-depth study, but given the interest generated by the question, we have performed a basic bioinformatic analysis using publicly available data.

To investigate the potential binding of Yamanaka factors to the promoter region of HOOK1, we extracted a 1 kb sequence upstream of the gene's transcription start site from the latest human

genome assembly (hg38) using the UCSC Genome Browser (http://genome.ucsc.edu/index.html). This sequence was then queried against the Jaspar database (http://jaspar.binf.ku.dk/) to identify putative transcription factor binding motifs, specifically for SOX2, OCT4, KLF4, and C-MYC. While some possible binding motifs were detected for SOX2 and OCT4, KLF4 emerged as the transcription factor with the most abundant and highest-scoring motifs. Consequently, our analysis suggests that if any Yamanaka factor were to interact with the HOOK1 promoter, KLF4 would be the principal candidate.

SOX2

Name	Score	Sequence ID	Start	End	Strand	Predicted sequence
MA0143.4.**SOX2**	9.6916895	hg38_dna	427	437	+	AGACAATGTGG

OCT4

Name	Score	Sequence ID	Start	End	Strand	Predicted sequence
MA1115.1.**POU5F1**	8.9693365	hg38_dna	276	286	-	AAATACAAAGA
MA1115.1.**POU5F1**	8.749028	hg38_dna	4	14	+	AAATGAAAACA

KLF4

Name	Score	Sequence ID	Start	End	Strand	Predicted sequence
MA0039.4.**KLF4**	12.931257	hg38_dna	781	792	+	CGCCCCGCCCCG
MA0039.4.**KLF4**	12.238994	hg38_dna	776	787	+	GCCCCCGCCCCG
MA0039.4.**KLF4**	12.191463	hg38_dna	619	630	+	ACCCCCGCCCTC
MA0039.4.**KLF4**	11.816491	hg38_dna	786	797	+	CGCCCCGCCCGC
MA0039.4.**KLF4**	11.493749	hg38_dna	613	624	+	CCTCCCACCCCC
MA0039.4.**KLF4**	11.089215	hg38_dna	894	905	-	CACCCCTCCCCT
MA0039.4.**KLF4**	11.081086	hg38_dna	656	667	+	CTTCCCACCCAC
MA0039.4.**KLF4**	11.079575	hg38_dna	953	964	-	GCCCCCTCCCCT
MA0039.4.**KLF4**	10.960708	hg38_dna	258	269	+	GCCACCACCCCC
MA0039.4.**KLF4**	10.236189	hg38_dna	899	910	-	TCTCCCACCCCT
MA0039.3.**KLF4**	9.981312	hg38_dna	440	450	-	CCACACTCTGC
MA0039.4.**KLF4**	9.646408	hg38_dna	151	162	-	CACCCCAGCCTG
MA0039.4.**KLF4**	9.466223	hg38_dna	986	997	-	CTCCTCACCCGC
MA0039.4.**KLF4**	9.315612	hg38_dna	882	893	-	CCTCCCACCCGT

*Nevertheless, the expression of HOOK1 may also be subject to modulation by Yamanaka factors, even in the absence of direct regulation of its promoter. To explore this possibility further, we analyzed RNA-seq data from OVCAR-3 ovarian cancer cells reprogrammed with the four Yamanaka factors. We observed a decrease in HOOK1 expression levels in the reprogrammed cell line compared to the parental counterpart (**REF**). This result suggests that, while Yamanaka factors may not directly induce HOOK1 expression, they could exert an indirect influence. However, further investigation across diverse cell lines and experimental verification are required to validate this observation. As proposed by the reviewer, investigating HOOK1 levels upon individual transcription factor elimination or reduction would provide additional insights into its regulatory mechanisms.*

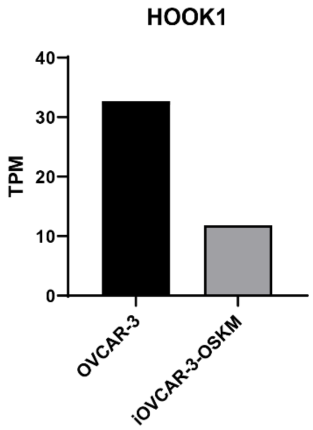

REF

Chen, Yu-An et al. "An experimental model for ovarian cancer: propagation of ovarian cancer initiating cells and generation of ovarian cancer organoids." BMC cancer vol. 22,1 967. 10 Sep. 2022, doi:10.1186/s12885-022-10042-3

*Concerning the proteome analysis a full list of the DE protein should be provided.

***Supplementary Figure 5** includes the full list of DE protein in our study. Additionally, the full list of proteins detected has been uploaded to ProteomXchange (https://www.proteomexchange.org/)*

- ***Submission details:***
  ***Project Name**: Protein homeostasis maintained by HOOK1 levels promotes the tumorigenic and stemness properties of ovarian cancer cells through reticulum stress and autophagy*
  ***Project accession**: PXD050782*
- ***Reviewer account details:***
  ***Username**: reviewer_pxd050782@ebi.ac.uk*
  ***Password**: GnLXzadr*

The section "Materials and Methods" has been updated to include this information and the citation of the software used to analyse the data:

Proteomic analysis

*[…] **Protein identification and label-free quantitation**. MS/MS spectra were searched against the reference proteome FASTA file (42161 entries; swissprot_2017_03_human_canonical_and_isoform). Enzyme specificity was set to trypsin, and up to two missed cleavages were allowed. Cysteine carboxamidomethylation (Cys, +57.021464 Da) was treated as fixed modification and methionine oxidation (Met, +15.994915 Da) and N-terminal acetylation (N-terminal, +42.010565 Da) as variable modifications. Peptide precursor ions were searched with a maximum mass deviation of 4.5 ppm and fragment ions with a maximum mass deviation of 20 ppm. Peptide and protein identifications were filtered at an FDR of 1% using the decoy database strategy. The minimal peptide length was seven amino acids. Proteins that could not be differentiated based on MS/MS spectra alone were grouped into protein groups (default MaxQuant settings). Searches were performed with the label-free quantification option selected. Proteins were quantified by spectral counting, that is, the number of identified MS/MS spectra for a given protein (Liu et al., 2004) combining the five fractions per sample. Raw counts were normalized on the sum of spectral counts for all identified proteins in a particular sample, relative to the average sample sum determined with all samples. To find statistically significant differences in normalized counts between sample groups, we applied the beta-binomial test (Pham et al., 2010), which takes into account within-sample and between-sample variation using an alpha level of 0.05. The mass spectrometry proteomics data have been deposited to the ProteomeXchange Consortium via the PRIDE [1] partner repository with the dataset identifier PXD050782. The generated data were filtered using the R platform [2] to obtain proteins whose expression increased (FC > 1) or decreased (FC < -1) significantly ($p < 0.05$) and analyzed using the ShinyGO 0.77 [3] (http://bioinformatics.sdstate.edu/go/) and STRING [4] (https://string-db.org/) platforms.*

(pages 23-24, lines 502-523)

REF

[1] Perez-Riverol Y, Bai J, Bandla C, García-Seisdedos D, Hewapathirana S, Kamatchinathan S, Kundu DJ, Prakash A, Frericks-Zipper A, Eisenacher M, Walzer M, Wang S, Brazma A, Vizcaíno JA. The PRIDE database resources in 2022: a hub for mass spectrometry-based proteomics evidences. Nucleic Acids Res. 2022 Jan 7;50(D1):D543-D552. doi: 10.1093/nar/gkab1038. PMID: 34723319; PMCID: PMC8728295.

[2] R Core Team (2021). R: A language and environment for statistical computing. R Foundation for Statistical Computing, Vienna, Austria. URL https://www.R-project.org/.

[3] Steven Xijin Ge, Dongmin Jung, Runan Yao, ShinyGO: a graphical gene-set enrichment tool for animals and plants, Bioinformatics, Volume 36, Issue 8, April 2020, Pages 2628–2629, https://doi.org/10.1093/bioinformatics/btz931

[4] Szklarczyk D, Franceschini A, Wyder S, et al. STRING v10: protein-protein interaction networks, integrated over the tree of life. Nucleic Acids Res. 2015;43(Database issue):D447-D452. doi:10.1093/nar/gku1003

*Given that the DE proteome is consistently different among the two cell lines with very few commonly modulated proteins, it is not clearly explained the rationale of choice to analyze and

show the expression of proteins reported in Figure 4E, which apparently are not among those commonly DE and have a high variable trend among clones also within a single cell line. Since this high variability, a true validation of the DE protein should be performed before exploring their possible functional impact.

*There is variability in the differentially expressed proteins between the two cell lines, although certain proteins exhibit similar alterations in both, as demonstrated in **Supplementary Figure 6C and D**. In fact, one of the proteins appearing in **Supp. Fig. 6C** (HSPA5/GRP78) has been analyzed by WB in **Figure 4E** to confirm the results of the proteomics. However, it is worth noting that in both cell lines, we found an alteration in the expression of proteins involved in protein folding in the ER. This prompted us to investigate whether there was an increase in ER stress due to an accumulation of misfolded proteins. To this end, we analyzed the expression of different proteins belonging to the ATF6 and PERK pathways, both activated in response to ER protein accumulation. We also assessed the sensitivity of cells with HOOK1 reduction to various ER stress-inducing compounds to confirm these results and ensure the increase in ER stress.*

We have not continued to explore other potentially dysregulated proteins from the proteomic analysis associated to different processes, although it would certainly be of interest for future research.

*Please improve the clarity of the description of figure 5, adding also some quantitative data to help to interpret the possible effect of the different treatments.

*We have assumed that the clarity issue arises in new **Figure 6**, which involves various treatments without accompanying WB quantification. We provide the quantification data for these WB analyses conducted on cells treated with TUDCA and chloroquine. Additionally, we have modified the manuscript text relating to this figure to improve readability and comprehension of the results.*

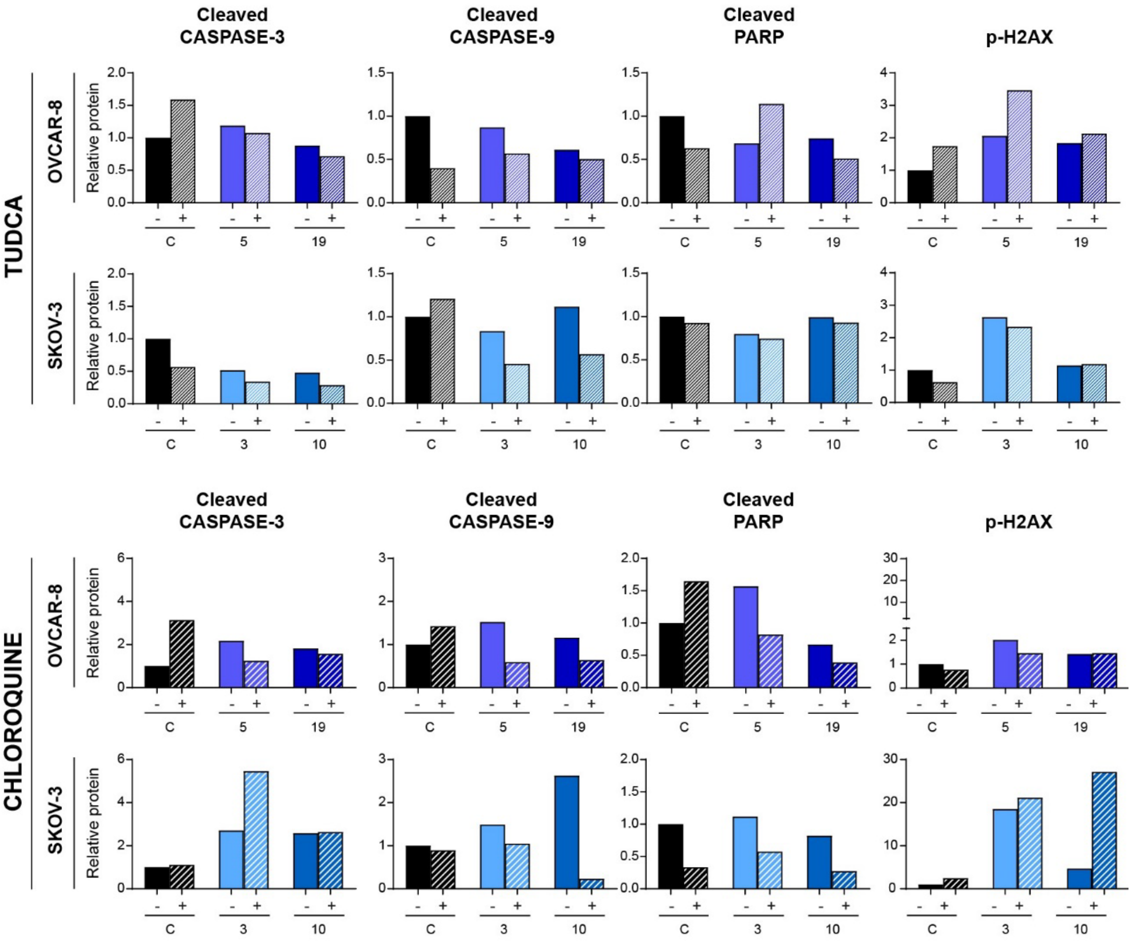

MANUSCRIPT

5. Cell death induced by Hook1 downregulation is dependent on the ER stress response and autophagic flux.

[…] As previously demonstrated, the reduction in HOOK1 levels is capable of activating apoptotic signaling pathways and causing an increase in cell death (Figure 4B, 4C). Therefore, we investigated whether the observed activation of the UPR in our model is responsible for this phenomenon. For that purpose, we used the drug tauroursodeoxycholic acid (TUDCA) to reduce ER stress levels (128). First, we confirmed that the drug effectively reduced ER stress in our model (Figure S4). Next, we studied cell death by flow cytometry and performed a WB of apoptosis-related markers. In the OVCAR-8 cell line, we observed that apoptotic markers did not change significantly in clone 19, while in clone 5, we observed an increase in cleaved PARP and p-H2AX. By flow cytometry, we found that TUDCA seemed to reduce cell death in clone 5; however, in clone 19, the changes in cell death were not as clear (Figure 6A). In the SKOV-3 cell line, there was a reduction in cleaved CASP3, CASP9, and PARP in CRISPR clone 10 but not in clone 3. Flow cytometry analysis showed that in both CRISPR clones, adding TUDCA to the medium reduced cell death. However, clone 10 exhibited a decrease in early-stage apoptosis, while clone 3 showed a reduction in necrosis, which may explain why we did not observe changes in apoptotic markers in this latter clone (Figure 6A). These results suggest that the inhibition of ER stress attenuates cell death in cells with reduced HOOK1 levels, although it does not completely rescue the phenotype.

*As previously demonstrated, reducing HOOK1 levels activates apoptotic signaling pathways, leading to increased cell death (**Figure 4B, 4C**). To investigate whether the activation of the UPR is responsible of this death, we used tauroursodeoxycholic acid (TUDCA) to reduce (ER) stress levels. Although TUDCA effectively reduced ER stress (**Figure S4**), its impact on cell death varied. In OVCAR-8 cells, apoptotic markers showed no significant changes, and TUDCA seemed to reduce cell death in clone 5 but had unclear effects in clone 19. In SKOV-3 cells, TUDCA reduced cleaved CASP3 and CASP9 and flow cytometry revealed reduced cell death in both clones, with clone 10 showing primarily a decrease in early-stage apoptosis and clone 3 displaying reduced necrosis (**Figure 6A**). Altogether, these findings suggest that inhibiting ER stress attenuates cell death in cells with reduced HOOK1 levels, albeit not completely rescuing the phenotype.*

*In contrast, activation of autophagic flux was reported to serve as a survival mechanism in cells with high ER stress. However, we found an impeded autophagic flux when we decreased HOOK1 levels (**Figure 5B, Supp. Fig. 2C**). Therefore, ~~we considered it important~~ in order to determine whether autophagy contributes protectively or inductively to cell death ~~in our model~~. ~~For this purpose,~~ we used chloroquine as an inhibitor of autophagic flux [32]. ~~As previously described,~~ We first confirmed its effectiveness by WB and immunofluorescence analyses ~~(Figure S5)~~ **(Supp. Fig. 10).** Subsequently, we observed that when we added chloroquine to cells with reduced HOOK1 levels, some apoptotic markers, such as ~~CASP3~~, CASP9 and PARP, were reduced in both cell lines (**Figure 6B**). However, by flow cytometry, we observed an increase in the percentage of dead cells after the treatment, which was significantly more pronounced in the cells with reduced HOOK1 levels. This increase was not due to the activation of apoptosis; instead, we found a very significant increase in the number of necrotic cells (Figure 6B). Therefore, these data suggest that cells with reduced HOOK1 levels are more sensitive to the blockage of autophagic flux, which likely acts as a protective mechanism to restore cellular proteostasis. ~~Furthermore, when autophagy is blocked, the preferred route for cell death in HOOK1-depleted cells is apoptosis-independent.~~ This result, coupled with the increase of vesicle colocalization in CRIPSR clones following the addition of brefeldin A (**Figure 5B, Supp. Fig. 2C**), indicates that autophagic flux is not completely blocked in cells with reduced levels of HOOK1, retaining some degree of functionality.*

(page 11-12, lines 233-260)

*Line 252 please clarify the statement about 'alteration in the autophagic flux'

In line 252, the mention of alterations in the autophagic flux specifically refers to the variations observed in autophagy discussed in the preceding section ("4. Hook1 downregulation causes changes in autophagic flux that are cell context dependent."). We have revised the sentence in line 252 to offer clearer context. (See previous answer).

*Discussion should be revised. It has not to be a mere summary of the results obtained. Figures' citation should be avoided and critical discussion has to be enhanced.

The discussion has been thoroughly revised to enhance its comprehensibility and critical component, as well as to avoid referencing figures from the results. Below are the changes made to the text:

Discussion

The downregulation of HOOK1 in ovarian cancer cell lines has resulted in a decrease in their tumorigenic and stemness properties while significantly increasing cell death. However, we have not found alterations in the expression of genes traditionally related to the stem cell phenotype that could explain this phenomenon. In some cases, we even found an increase of the expression of those genes, as in the case of KLF4. This could be explained by the increased ER stress observed when HOOK1 levels decrease. A recent study linked increased ER stress to the induction of KLF4 expression in melanoma cells, as an adaptive mechanism inhibiting UPR-induced apoptosis and promoting metastasis [33]. Furthermore, other studies have observed that the increase in GRP78 can promote the expression of OCT4 in head and neck and breast cancer cells [34, 35]. Hence, it is worth considering that the increase in ER stress may account for the lack of reduction in these genes. ~~HOOK1 was found to be overexpressed in ovarian cancer tumors (**Figure 1A**), and its downregulation in cell lines of this tumor resulted in a decrease in cell proliferative capacity (**Figure 1C, 1D**) and migration (**Figure 2A**) while significantly increasing cell death (**Figure 2B**). Moreover, xenografting these cells into mice failed to form tumors (**Figure 1E**). These data suggest a clear role for HOOK1 in the generation or maintenance of ovarian tumors. Additionally, some properties related to CSCs were reduced when HOOK1 levels decreased (**Figure 3A, 3B**). However, we did not observe changes in genes traditionally related to the stem cell phenotype. In some cases, we found an increase in some genes, such as *KLF4* (**Figure 3C, 3D**). Although this increase seems to contradict the decrease in CSC properties, it could be explained by the increased endoplasmic reticulum (ER) stress observed when HOOK1 levels decrease (**Figure 4**). A recent study linked increased ER stress to the induction of *KLF4* expression in melanoma cells. *KLF4* expression acts as an adaptive mechanism against ER stress, inhibiting UPR-induced apoptosis and promoting metastasis [25].~~

Tumor cells commonly experience ER stress due to various stress sources [36]. While no established link between HOOK1 and ER stress exists, we can speculate on potential causes. ~~The occurrence of ER stress is common in tumor cells due to the numerous sources of stress that they experience [26]. To date, no relationship has been described between the HOOK1 protein and the occurrence of ER stress. However, we can speculate about the possible causes of this phenomenon.~~ The protein HOOK2, from the same family as HOOK1, is involved in the formation and maintenance of aggresomes [37], structures whose inhibition has been associated with accumulation of misfolded proteins and ER stress [38, 39]. HOOK1 might play a similar role in the formation of this structure, possibly through the transport of ~~HOOK1 could be involved in transporting~~ misfolded proteins to this structure due to its role in retrograde ~~protein~~ transport. Notably, there are structural and functional similarities between the HDAC6 protein, which is essential for aggresome formation, and the HOOK protein family [11, 38]. Additionally, the FHF complex (HOOK, FTS, and FHIP) has been associated with the motility of tubular intermediaries that allow ER transport to the Golgi apparatus [14]. Hindered transport between these two organelles could also lead to increased ER stress.

~~In addition to UPR activation, cells with reduced HOOK1 levels show changes in autophagic flux (**Figure 5**). In recent years, a strong interconnection between the UPR and autophagy has been described [29]. The role of autophagy in cancer is complex and context dependent. Its function in maintaining homeostasis plays an important role in preventing tumorigenesis. However,~~

~~autophagic induction in tumors exposed to hypoxia and/or nutrient deprivation can provide the necessary energy to promote cell survival and chemoresistance [26]. In contrast, excessive autophagic activation can also trigger cell death through different mechanisms [30]. Therefore, the exact role of autophagy in cancer is dependent on tumor type, stage, and tumor microenvironmental conditions. In cells with reduced HOOK1 levels, UPR activation would be expected to increase autophagic flux. However, the results suggest that in the OVCAR-8 cell line, decreased HOOK1 levels indeed lead to an increase in autophagy, while in the SKOV-3 cell line, HOOK1 reduction seems to cause some kind of blockage in autophagic flux (**Figure 5**). This blockage would prevent autophagosome-lysosome fusion, leading to an accumulation of these structures.~~

~~This partial blockage of autophagy could be related to the interaction of the FHF complex, including HOOK proteins, with the HOPS complex. When one of the FHF complex proteins is eliminated, the ability of Vps18 to promote lysosome clustering is compromised. These data suggest that the FHF complex may help coordinate the movement or interaction of vesicles through the HOPS complex [13]. The HOPS complex is involved in the fusion of late endosomes and autophagosomes with lysosomes in multiple organisms. Therefore, the loss of any of its subunits leads to the accumulation of late endosomes and autophagosomes [31-33]. Thus, it is possible that HOOK1 loss blocks autophagic flux by interfering with the proper functioning of the HOPS complex. Therefore, studying the interaction of HOOK1 with the HOPS complex in the future could help elucidate why autophagic flux might be blocked in our model and why it seems to affect the two studied cell lines differently.~~

Independently from its origin, ~~Both the increase in~~ increased ER stress ~~and the partial blockage of autophagic flux~~ could be responsible for the ~~increase~~ elevation in cell death observed in cells with downregulated HOOK1 ~~(**Figure 2B**)~~. Sustained ER stress is cytotoxic to cells, leading to the activation of the apoptotic pathway and other cell death mechanisms [40, 41]. We found that reducing ER stress in our model partially reverses cell death, indicating that ER stress may be responsible, at least in part, for this death. ~~To corroborate the existence of a cytotoxic effect of ER stress in our model, we used TUDCA to reduce it. We found that the use of TUDCA partially reverses cell death, indicating that ER stress may be responsible, at least in part, for this death.~~ However, we did not observe a complete reversal of the phenotype ~~when adding the drug (**Figure 6A**)~~. This could be explained because the inhibition of ER stress was not sufficiently effective, although we did observe a reduction in ER stress markers when using TUDCA ~~(**Figure S4**)~~. Therefore, it is possible that cells with reduced HOOK1 levels experience increased cell death due to more than one factor.

In addition to UPR activation, cells with reduced HOOK1 levels show changes in autophagic flux. In recent years, a strong interconnection between the UPR and autophagy has been described [31]. The role of autophagy in cancer is complex and context dependent. Its function in maintaining homeostasis plays an important role in preventing tumorigenesis. However, autophagic induction in tumors exposed to hypoxia and/or nutrient deprivation can provide the necessary energy to promote cell survival and chemoresistance [36]. In contrast, excessive autophagic activation can also trigger cell death through different mechanisms [42]. Therefore, the exact role of autophagy in cancer is dependent on tumor type, stage, and tumor microenvironmental conditions. In cells with reduced HOOK1 levels, UPR activation would be

expected to increase autophagic flux. However, our results suggest that the decrease in HOOK1 could cause some kind of blockage in this process, that would prevent autophagosome-lysosome fusion, leading to an accumulation of these structures.

This partial blockage of autophagy could be related to the interaction of the FHF complex, including HOOK proteins, with the HOPS complex. It has been suggested that the FHF complex may help coordinate the movement or interaction of vesicles through the HOPS complex [13], which is involved in the fusion of late endosomes and autophagosomes with lysosomes in multiple organisms. Therefore, the loss of any of its subunits leads to the accumulation of late endosomes and autophagosomes [43-45]. Thus, it is possible that HOOK1 loss blocks autophagic flux by interfering with the proper functioning of the HOPS complex. Studying the interaction of HOOK1 with the HOPS complex in the future could help elucidate why autophagic flux might be partially impeded in our model.

Both excessive activation and blockade of autophagy can lead to cell death through apoptotic signaling in tumor cells [46, 47]. However, the ability of autophagy to suppress necrotic death is considered one of its most important prosurvival mechanisms [48, 49], although other studies have shown that autophagy can promote necroptosis [50, 51]. Therefore, the involvement of autophagy in cell survival or death is complex and highly dependent on the cellular context. Generally, an increase in ER stress induces a protective increase in autophagic flux to reduce proteotoxic stress and avoid cell death. Our results show that blocking autophagic flux affect cells with reduced HOOK1 levels to a greater extent than parental cells, leading to a significant increase in cell death, especially necrosis. This finding correlates with the role of autophagy as a suppressor of necrotic death and suggests that the autophagic pathway may act as a protective mechanism against ER stress in our model. However, this prosurvival mechanism might not function perfectly, as the decrease in HOOK1 also appears to cause a partial blockade of autophagic flux. Therefore, it may not be as effective as expected in preventing cell death.

~~For instance, it could be due to alterations in autophagic flux. Both excessive activation and blockade of autophagy can lead to cell death through apoptotic signaling in tumor cells [36,37]. However, the ability of autophagy to suppress necrotic death is considered one of its most important prosurvival mechanisms [38,39], although other studies have shown that autophagy can promote necroptosis [40,41]. Therefore, the involvement of autophagy in cell survival or death is complex and highly dependent on the cellular context. Generally, an increase in ER stress induces a protective increase in autophagic flux to reduce proteotoxic stress and avoid cell death. To verify whether autophagy plays this protective role when HOOK1 is reduced, we used chloroquine to completely block autophagic flux. We observed that the use of this drug affected cells with reduced HOOK1 levels to a greater extent than parental cells, leading to a significant increase in cell death (**Figure 6B**). Additionally, when autophagic flux is blocked, the preferred mode of cell death is necrosis. This finding correlates with the role of autophagy as a suppressor of necrotic death. These data suggest that the autophagic pathway may act as a protective mechanism against ER stress in our model. However, the previous data indicate that this prosurvival mechanism may not function perfectly, as the decrease in HOOK1 also appears to cause a partial blockade of autophagic flux. Therefore, it may not be as effective as expected in preventing cell death (**Figure 8**).~~

The occurrence of ER stress and the problems in autophagic flux could also explain the changes observed in terms of CSC properties when HOOK1 is reduced. We observed that the downregulation of this gene led to an increase in the death of EpCAM+-positive cells in the ovarian cancer two cell lines studied (**Figure 7A**). This result suggests that the reduction in HOOK1, not only causes a decrease in the survival of ovarian cancer cells in general, but also specifically affects CSCs. Numerous studies have shown that maintaining ER proteostasis and autophagy flux intact is crucial for maintaining CSC integrity. For example, UPR induction can decrease CSC properties in colorectal carcinoma [42,43], breast cancer [44], head and neck cancer [45], glioblastoma [46,47], and prostate carcinoma [48]. Moreover, autophagy has been linked to the maintenance of CSCs in different tumors, such as breast cancer [49,50], pancreatic and liver carcinoma [51], osteosarcoma [52], ovarian cancer [53], head and neck cancer [54], and glioblastoma [55]. We observed in our model that increased ER stress or blockade of autophagy can reproduce the cell death of CSCs observed when HOOK1 is downregulated (**Figure 7B**). This finding further supports the hypothesis that the decrease in stem cell properties when HOOK1 is reduced may be due to this phenomenon these two phenomena. The partial blockade of autophagy could also impact the survival of CSCs. Autophagy has been linked to the maintenance of CSCs in different tumors, such as breast cancer [59, 60], pancreatic and liver carcinoma [61], osteosarcoma [62], ovarian cancer [63], head and neck cancer [64], and glioblastoma [65]. Consistent with these studies, we have observed that blocking autophagic flux with chloroquine also increases CSC death and reduces their proportion within the tumor cell culture.

Given that reducing HOOK1 levels leads to the occurrence of proteotoxic stress and compromises the survival of CSCs, inhibiting this protein could be a promising therapeutic strategy in ovarian tumors. In fact, we observed that the decrease in HOOK1 sensitizes cells to compounds inducing ER stress and to proteasome inhibitors and that inhibiting HOOK1 prevented tumor formation in mouse models (**Figure 4F**). Drugs capable of inducing ER stress or blocking autophagy have been widely used in studies to treat different types of tumors. For ER stress, preclinical studies suggest the utility of potent activation of this mechanism to kill tumor cells [56-59]. In the case of autophagy, numerous studies have described that blocking this pathway can produce a cytotoxic effect in tumor cells. Two drugs that block the autophagic flux have been approved for clinical use, chloroquine and its derivative hydroxychloroquine (HCQ) , and they have been used in clinical trials of adenocarcinoma, melanoma, colorectal carcinoma, myeloma, lymphoma, and renal carcinoma [60]. Hence, autophagy blockade is currently considered a promising potential strategy in the treatment of tumors refractory to conventional therapies. Based on the described use of drugs affecting proteostasis as therapeutic strategies in cancer, HOOK1 could be a promising therapeutic target, since reducing this protein affects multiple cellular processes capable of triggering proteotoxic stress in tumor cells. Moreover, we observed that inhibiting HOOK1 prevented tumor formation in mouse models (**Figure 1E**). Therefore, it would be interesting to study HOOK1 inhibition as a potential treatment for ovarian cancer, either as monotherapy or in combination with chemotherapeutic agents or other drugs inducing proteotoxic stress, such as inhibitors of the proteasome or enhancers promoters of the UPR response.

(pages 14-18, lines 285-382)

*References must be entirely revised and reformatted. Reffs #1 to #3 appear to be unrelated to the text, as a consequence the whole indexing is shifted and it is not possible to check the appropriateness of all the other ones.

We have reviewed the references to ensure that all of them have been correctly inserted into the text.
